# Supplementary material for: Relationship between journal impact factor and the thoroughness and helpfulness of peer reviews
Source: PLoS Biol. 2023 Aug 29;21(8):e3002238. doi: 10.1371/journal.pbio.3002238 (PMC10464996; doi:10.1371/journal.pbio.3002238)

Supporting information

Severin et al.: Relationship between Journal Impact Factor and the Thoroughness and Helpfulness of Peer Reviews

S2 File. Further details on classification and validation

Further information on the hand-coded set of sentences, the classification approach and performance, provide metrics on the classification performance, and show that aggregating the classification closely mirrors human coding of the same set of sentences. All results are out-of-sample predictions, meaning that the data in the held-out test set are not used for training the classifier during validation steps.

Figure S2.1 shows the number of sentences in the set of annotated sentences allocated to each of the content categories. As described in the main paper, 1,160 sentences were allocated to more than one category.

Figure S2.2. shows the correlation between the number of sentences in the set of annotated sentences and the prevalence of the same content categories in the full text corpus.

Figure S2.3 reports the performance metrics for the out-of-sample classification based on the eight classifiers. We report precision, recall, and the F1 score separately for the "presence" of a content category and the "absence" of a content category.

Figure S2.4 reports the balanced accuracy for the eight classifiers. For binary classifiers, the balanced accuracy is calculated as:

$$Balanced\ Accuracy\ = \frac{Recall + Precision}{2}\ .$$

Due to limited computational resources and satisfactory results, we used the default parameters when fine-tuning the eight DistilBERT language models.[1] In the replication archive, we provide the eight fine-tuned DistilBERT models along with a short tutorial on how to classify sentences: https://doi.org/10.5281/zenodo.8006829

[1] These paramaters are as follows: _name_or_path: "distilbert-base-uncased"; activation: "gelu"; architectures: "DistilBertForSequenceClassification"; attention_dropout: 0.1; dim: 768; dropout: 0.1; hidden_dim: 3072; initializer_range: 0.02; max_position_embeddings: 512; model_type: "distilbert"; n_heads: 12; n_layers: 6; pad_token_id: 0; problem_type: "single_label_classification"; qa_dropout: 0.1; seq_classif_dropout: 0.2; sinusoidal_pos_embds: false; tie_weights_: true; torch_dtype: "float32"; transformers_version: "4.20.1"; vocab_size: 30522.

Figure S2.1: The number of sentences in the set of annotated sentences allocated to each of the content categories.
Note: sentences could be allocated to more than one category. The data underlying this figure can be found in S7 Data.

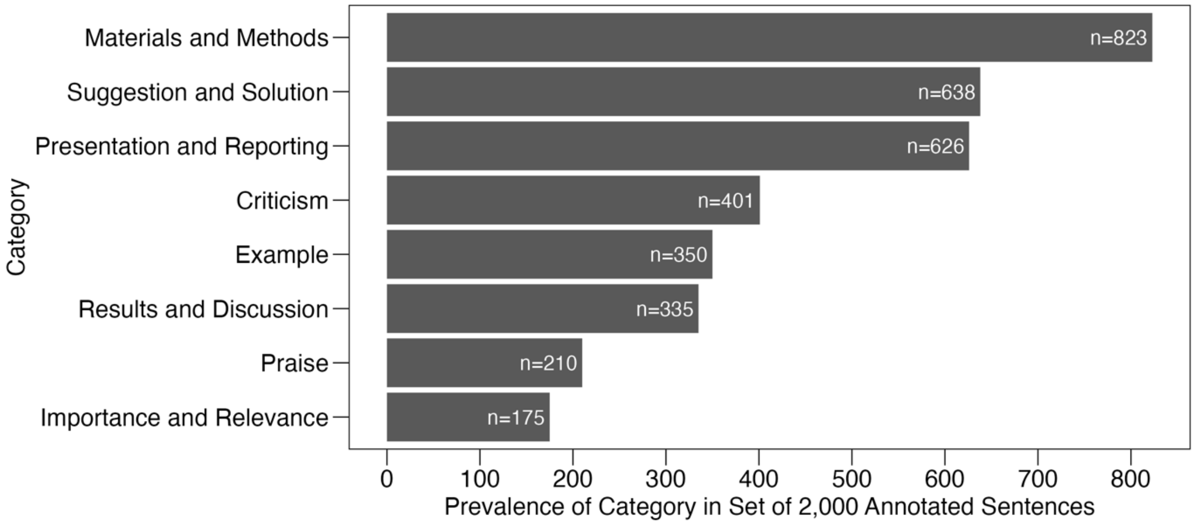

Figure S2.2: The correlation between the number of sentences in the annotated set of sentences and prevalence of content categories in the full text corpus.
The data underlying this figure can be found in S8 Data.

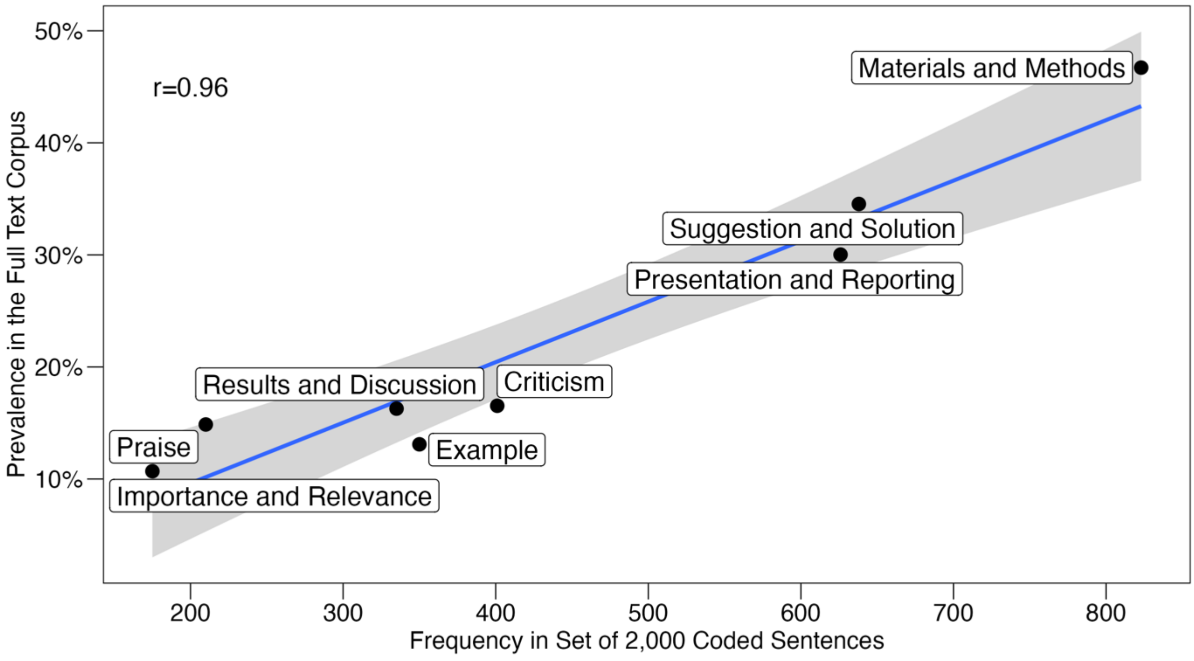

Figure S2.3: Precision, Recall, and F1 scores for out-of-sample predictions based on DistilBERT-based classifiers.
The data underlying this figure can be found in S9 Data.

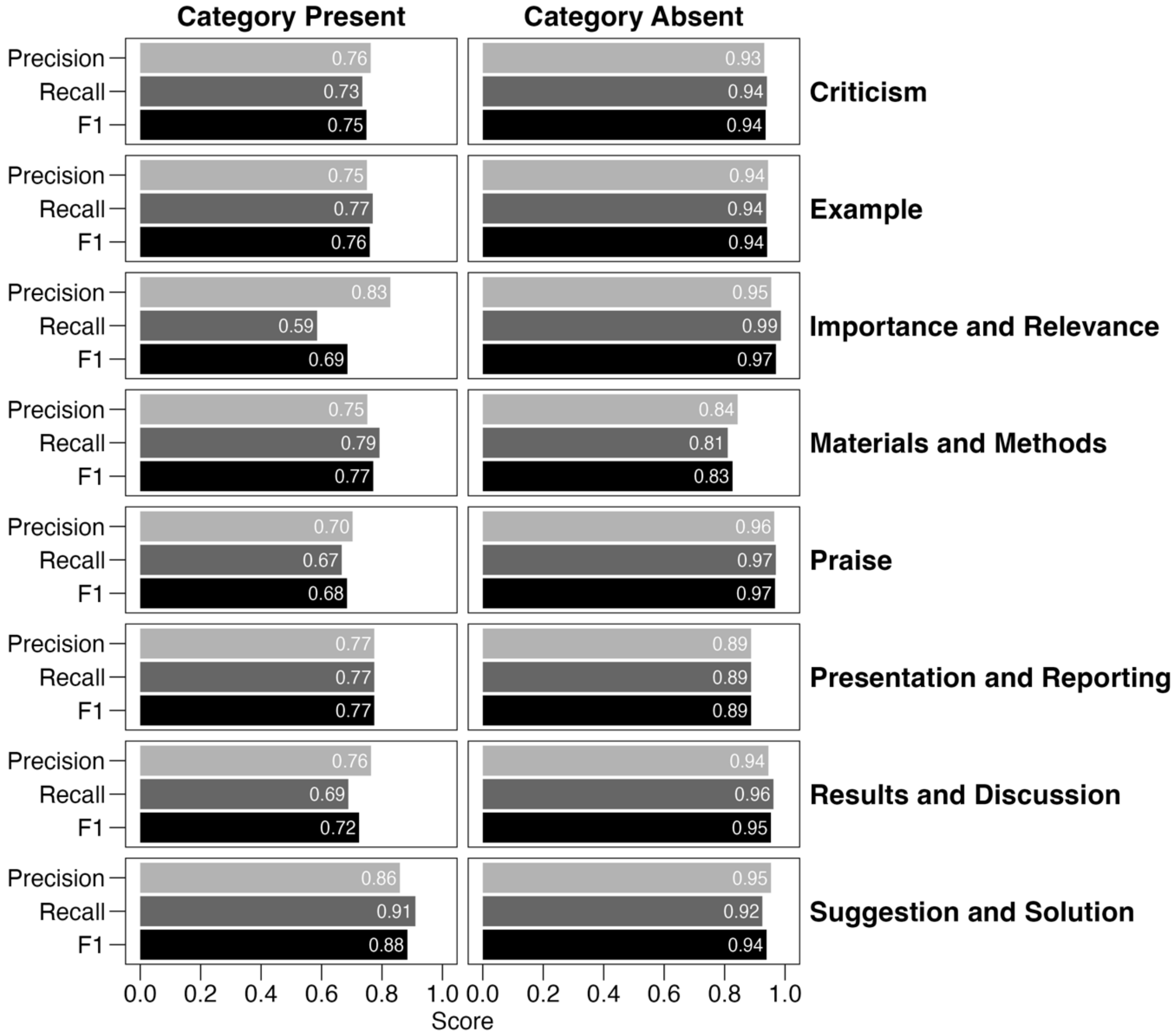

Figure S2.4: Balanced accuracy for out-of-sample predictions based on DistilBERT-based classifiers.
The data underlying this figure can be found in S10 Data.

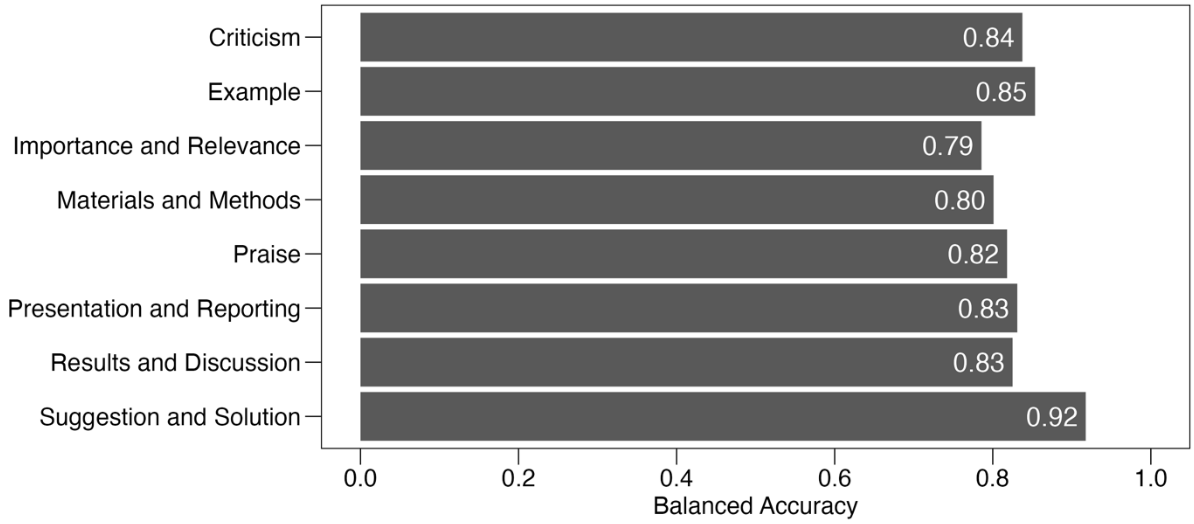

Supplement: S2 File — Further information on the hand-coded set of sentences, the classification approach, and performance provide metrics on the classification performance and show that aggregating the classification closely mirrors human coding of the same set of sentences. All results are out-of-sample predictions, meaning that the data in the held-out test set are not used for training the classifier during validation steps. (PDF) [file pbio.3002238.s002.pdf]
