# Supplementary material for: Relationship between journal impact factor and the thoroughness and helpfulness of peer reviews
Source: PLoS Biol. 2023 Aug 29;21(8):e3002238. doi: 10.1371/journal.pbio.3002238 (PMC10464996; doi:10.1371/journal.pbio.3002238)
Supplement: S3 File — All regression tables for the analysis reported in the paper, and plots and regression tables relating to the 5 sensitivity analyses. All sensitivity analyses are conducted for the prevalence-based and sentence-based models. (PDF) [file pbio.3002238.s003.pdf]

Supporting information

Severin et al.: Relationship between Journal Impact Factor and the Thoroughness and Helpfulness of Peer Reviews

S3 File. Additional details on regression analyses and sensitivity analyses

All regression tables for the analysis reported in the paper, and plots and regression tables relating to the five sensitivity analyses. All sensitivity analyses are conducted for the prevalence-based and sentence-based models.

While the regression results closely mirror the descriptive plots, the negative binomial regression models had conversion issues due to the low number of reviews for some of the journals and reviewers and the highly skewed distributions. of counts Results are virtually identical when rerunning the models when running negative binomial regression models without random intercepts for journals and reviewers.

Regression tables for the results reported in the main paper

Fig 4 paper relies on the coefficients in Tables S3.1 and S3.2. Tables S3.3 and S3.4 contain the coefficients visualised in Fig 5.

Table S3.1: Predicting the prevalence of categories related to 'thoroughness'. Table shows coefficients from negative binomial regression models. Standard errors in parentheses.

	**Materials and Methods**	**Presentation and Reporting**	**Results and Discussion**	**Importance and Relevance**
(Intercept)	6.07 (0.39)***	4.78 (0.28)***	2.25 (0.17)***	1.39 (0.09)***
JIF group: 2 (ref.: JIF group: 1)	1.99 (0.56)***	1.37 (0.41)***	0.77 (0.25)**	0.23 (0.13)
JIF group: 3	1.77 (0.60)**	0.61 (0.43)	0.68 (0.27)*	0.17 (0.14)
JIF group: 4	3.06 (0.61)***	1.29 (0.44)**	1.08 (0.27)***	0.27 (0.15)
JIF group: 5	3.65 (0.58)***	1.69 (0.42)***	1.24 (0.26)***	0.26 (0.14)
JIF group: 6	3.21 (0.60)***	1.02 (0.43)*	0.74 (0.27)**	0.09 (0.14)
JIF group: 7	4.57 (0.60)***	1.60 (0.43)***	1.48 (0.27)***	0.38 (0.14)**
JIF group: 8	4.19 (0.62)***	1.29 (0.45)**	1.37 (0.28)***	0.47 (0.15)**
JIF group: 9	4.86 (0.66)***	1.55 (0.47)**	1.54 (0.29)***	0.48 (0.16)**
JIF group: 10	6.47 (0.63)***	1.53 (0.46)***	1.68 (0.28)***	0.80 (0.15)***
AIC	72257.46	66401.31	55458.71	41065.90
Log Likelihood	-36115.73	-33187.66	-27716.36	-20519.95
N	10000	10000	10000	10000
N groups: Journal ID	1644	1644	1644	1644
N groups: Reviewer ID	9259	9259	9259	9259

***$p < 0.001$; **$p < 0.01$; *$p < 0.05$

Table S3.2: Predicting the prevalence of categories related to 'helpfulness'. Table shows coefficients from negative binomial regression models. Standard errors in parentheses.

	**Suggestion and Solution**	**Example**	**Criticism**	**Praise**
(Intercept)	5.17 (0.29)***	2.12 (0.24)***	2.33 (0.16)***	1.46 (0.07)***
JIF group: 2 (ref.: JIF group: 1)	1.43 (0.41)***	1.01 (0.34)**	0.78 (0.23)***	0.22 (0.11)*
JIF group: 3	0.61 (0.44)	0.60 (0.36)	0.50 (0.25)*	0.16 (0.12)
JIF group: 4	1.55 (0.45)***	1.08 (0.37)**	1.11 (0.25)***	0.08 (0.12)
JIF group: 5	1.86 (0.43)***	1.45 (0.35)***	1.02 (0.24)***	0.25 (0.11)*
JIF group: 6	1.13 (0.44)*	1.25 (0.36)***	0.67 (0.25)**	0.16 (0.12)
JIF group: 7	2.01 (0.44)***	1.32 (0.36)***	1.25 (0.25)***	0.37 (0.11)**
JIF group: 8	1.93 (0.46)***	1.19 (0.38)**	1.21 (0.26)***	0.43 (0.12)***
JIF group: 9	1.86 (0.49)***	1.75 (0.40)***	1.76 (0.27)***	0.43 (0.13)***
JIF group: 10	2.09 (0.47)***	1.79 (0.38)***	1.94 (0.26)***	0.68 (0.12)***
AIC	66332.47	62548.88	54783.34	37834.29
Log Likelihood	-33153.24	-31261.44	-27378.67	-18904.15
N	10000	10000	10000	10000
N groups: Journal ID	1644	1644	1644	1644
N groups: Reviewer ID	9259	9259	9259	9259

$^{***}p < 0.001$; $^{**}p < 0.01$; $^{*}p < 0.05$

Table S3.3: Predicting the prevalence of categories related to 'thoroughness'. Table shows coefficients from mixed effects linear regression models. Standard errors in parentheses.

	**Materials and Methods**	**Presentation and Reporting**	**Results and Discussion**	**Importance and Relevance**
(Intercept)	39.66 (0.99)***	33.02 (0.77)***	15.00 (0.55)***	12.46 (0.51)***
JIF group: 2 (ref.: JIF group: 1)	3.53 (1.41)*	-1.68 (1.10)	1.48 (0.78)	-1.03 (0.73)
JIF group: 3	3.93 (1.50)**	-0.54 (1.15)	1.18 (0.81)	-1.85 (0.75)*
JIF group: 4	5.97 (1.52)***	-1.98 (1.17)	2.28 (0.83)**	-1.94 (0.77)*
JIF group: 5	7.13 (1.46)***	-1.57 (1.13)	1.22 (0.80)	-1.74 (0.74)*
JIF group: 6	7.69 (1.49)***	-3.90 (1.14)***	-0.63 (0.81)	-2.77 (0.75)***
JIF group: 7	8.68 (1.49)***	-2.48 (1.14)*	1.49 (0.81)	-2.38 (0.75)**
JIF group: 8	8.42 (1.54)***	-4.36 (1.17)***	1.95 (0.83)*	-1.77 (0.76)*
JIF group: 9	9.67 (1.61)***	-4.70 (1.21)***	1.88 (0.85)*	-1.88 (0.79)*
JIF group: 10	11.02 (1.57)***	-7.69 (1.20)***	1.12 (0.85)	-1.94 (0.78)*
AIC	92429.26	89594.15	83250.79	82207.16
BIC	92523.00	89687.88	83344.52	82300.89
Log Likelihood	-46201.63	-44784.07	-41612.39	-41090.58
N	10000	10000	10000	10000
N groups: Reviewer ID	9259	9259	9259	9259
N groups: Journal ID	1644	1644	1644	1644

$^{***}p < 0.001$; $^{**}p < 0.01$; $^{*}p < 0.05$

Table S3.4: Predicting the prevalence of categories related to 'helpfulness'. Table shows coefficients from mixed effects linear regression models. Standard errors in parentheses.

	**Suggestion and Solution**	**Example**	**Criticism**	**Praise**
(Intercept)	36.80 (0.76)***	11.35 (0.65)***	15.92 (0.51)***	16.60 (0.70)***
JIF group: 2 (ref.: JIF group: 1)	-1.61 (1.08)	2.60 (0.93)**	0.15 (0.73)	-0.72 (0.99)
JIF group: 3	-0.80 (1.13)	2.55 (0.99)*	-0.03 (0.74)	-1.06 (1.02)
JIF group: 4	-0.85 (1.15)	2.06 (1.01)*	1.83 (0.75)*	-2.24 (1.04)*
JIF group: 5	-0.96 (1.11)	2.72 (0.97)**	-0.21 (0.73)	-1.79 (1.01)
JIF group: 6	-3.60 (1.13)**	2.25 (0.99)*	0.51 (0.74)	-1.99 (1.02)
JIF group: 7	-2.44 (1.12)*	2.53 (0.99)*	0.40 (0.74)	-3.06 (1.02)**
JIF group: 8	-2.19 (1.15)	1.63 (1.02)	0.21 (0.74)	-2.67 (1.04)*
JIF group: 9	-4.56 (1.19)***	3.38 (1.07)**	2.50 (0.75)***	-2.85 (1.07)**
JIF group: 10	-6.34 (1.18)***	2.43 (1.04)*	1.03 (0.75)	-2.40 (1.06)*
AIC	89173.62	83840.77	83345.33	88328.21
BIC	89267.36	83934.50	83439.07	88421.95
Log Likelihood	-44573.81	-41907.38	-41659.67	-44151.11
N	10000	10000	10000	10000
N groups: Reviewer ID	9259	9259	9259	9259
N groups: Journal ID	1644	1644	1644	1644

$^{***}p < 0.001$; $^{**}p < 0.01$; $^{*}p < 0.05$

Sensitivity analysis 1: Remove reviews with a prevalence of 0% in the respective content category

In the first sensitivity analysis, reviews with a prevalence of 0% or 0 sentences in a respective content category were removed before running the regression models. For this reason, the number of observations differ across the regression models. Figure S3.1 shows the predicted counts for this subset of reviews. Figure S3.2 shows the percentage point changes in sentences addressing content categories across the JIF Groups (with JIF Group = 1 as the reference category) for these subsets of the sample. Tables S3.5–S3.8 report the full regression models used to create these plots.

Fig S3.1. Predicted number of sentences addressing thoroughness and helpfulness categories across the ten Journal Impact Factor groups.
Predicted values and 95% confidence intervals are shown. Models exclude reviews without mentions of a content category. All negative binomial mixed-effects models include random intercepts for the journal name and reviewer ID. The data underlying this figure can be found in S11 Data.

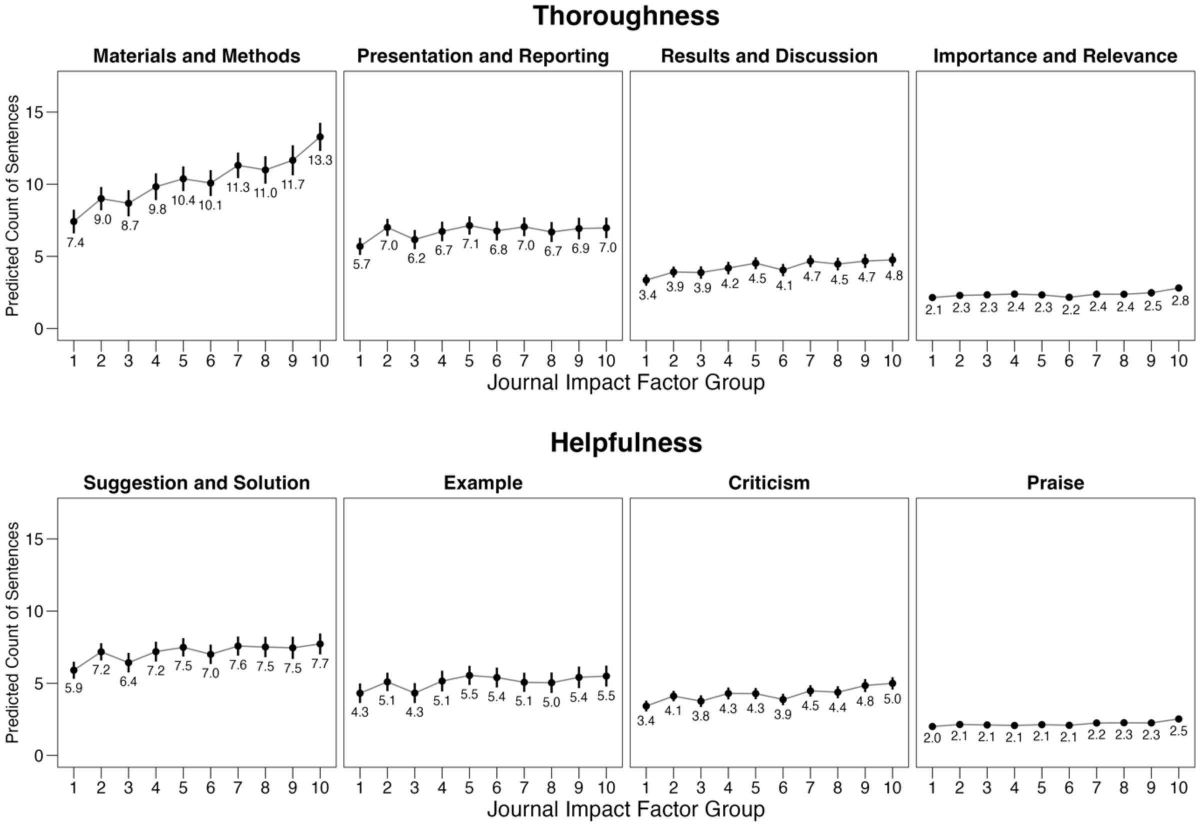

Fig S3.2. Percentage point change in the proportion of sentences addressing thoroughness and helpfulness categories relative to the lowest Journal Impact Factor group.

Regression coefficients and 95% confidence intervals are shown. Models exclude reviews without mentions of a content category. All linear mixed-effects models include random intercepts for the journal name and reviewer ID. The data underlying this figure can be found in S12 Data.

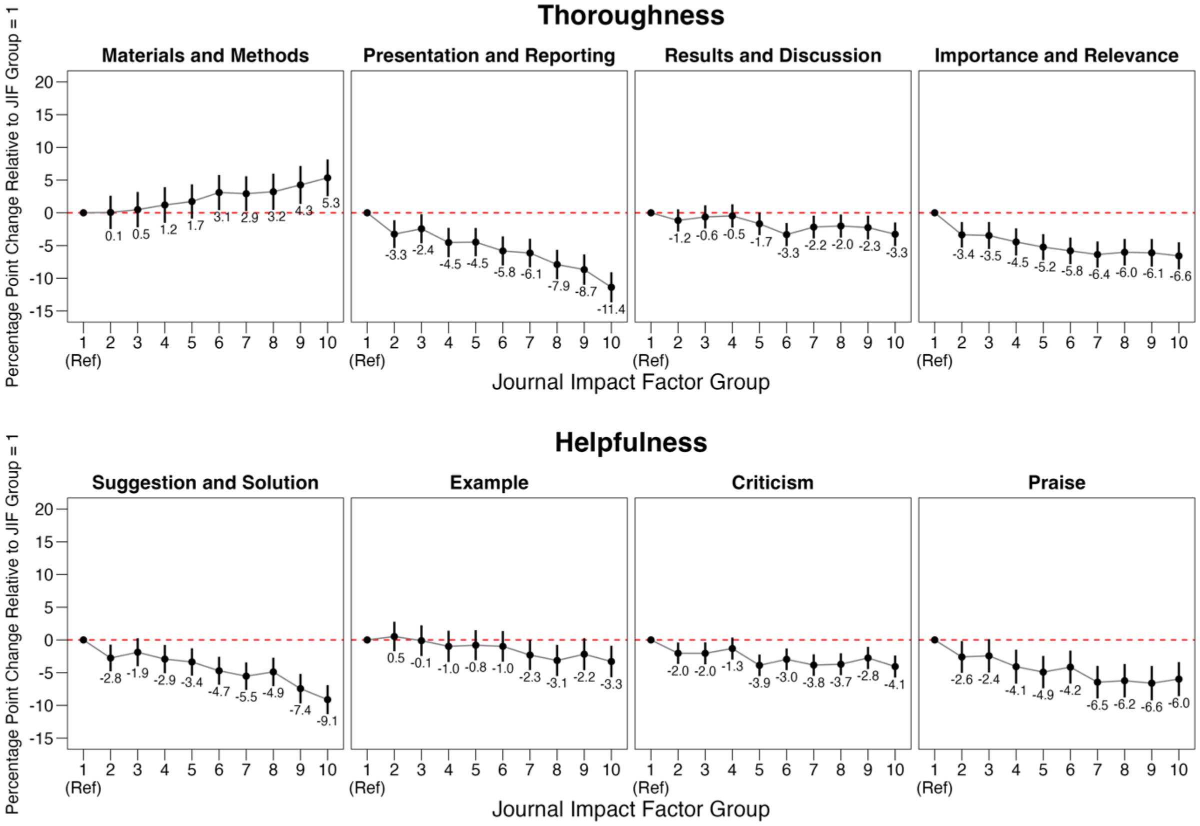

Table S3.5: Predicting the prevalence of categories related to 'thoroughness'. Models exclude reviews without mentions of a content category. Table shows coefficients from negative binomial regression models. Standard errors in parentheses.

	**Materials and Methods**	**Presentation and Reporting**	**Results and Discussion**	**Importance and Relevance**
(Intercept)	7.41 (0.42)***	5.69 (0.30)***	3.35 (0.20)***	2.15 (0.11)***
JIF group: 2 (ref.: JIF group: 1)	1.59 (0.59)**	1.31 (0.43)**	0.56 (0.28)*	0.15 (0.15)
JIF group: 3	1.26 (0.62)*	0.47 (0.45)	0.53 (0.30)	0.19 (0.16)
JIF group: 4	2.41 (0.63)***	1.03 (0.46)*	0.83 (0.30)**	0.24 (0.16)
JIF group: 5	2.96 (0.60)***	1.45 (0.44)**	1.17 (0.29)***	0.18 (0.15)
JIF group: 6	2.67 (0.62)***	1.07 (0.45)*	0.70 (0.29)*	0.02 (0.16)
JIF group: 7	3.89 (0.62)***	1.36 (0.45)**	1.31 (0.29)***	0.24 (0.16)
JIF group: 8	3.57 (0.64)***	0.99 (0.46)*	1.11 (0.30)***	0.23 (0.16)
JIF group: 9	4.24 (0.68)***	1.24 (0.49)*	1.32 (0.32)***	0.33 (0.17)
JIF group: 10	5.87 (0.65)***	1.28 (0.47)**	1.40 (0.30)***	0.66 (0.16)***
AIC	65595.05	58254.03	42347.80	27937.02
Log Likelihood	-32784.52	-29114.01	-21160.90	-13955.51
N	9070	8717	7528	6693
N groups: Journal ID	1582	1582	1496	1475
N groups: Reviewer ID	8446	8139	7073	6350

$^{***}p < 0.001$; $^{**}p < 0.01$; $^{*}p < 0.05$

Table S3.6: Predicting the prevalence of categories related to 'helpfulness'. Models exclude reviews without mentions of a content category. Table shows coefficients from negative binomial regression models. Standard errors in parentheses.

	**Suggestion and Solution**	**Example**	**Criticism**	**Praise**
(Intercept)	5.90 (0.30)***	4.31 (0.35)***	3.42 (0.19)***	2.00 (0.08)***
JIF group: 2 (ref.: JIF group: 1)	1.27 (0.43)**	0.78 (0.48)	0.69 (0.26)**	0.14 (0.11)
JIF group: 3	0.52 (0.46)	0.01 (0.49)	0.34 (0.28)	0.11 (0.12)
JIF group: 4	1.29 (0.46)**	0.84 (0.50)	0.87 (0.28)**	0.07 (0.12)
JIF group: 5	1.59 (0.44)***	1.23 (0.48)*	0.86 (0.27)**	0.13 (0.11)
JIF group: 6	1.10 (0.46)*	1.09 (0.49)*	0.45 (0.28)	0.09 (0.12)
JIF group: 7	1.67 (0.45)***	0.76 (0.49)	1.05 (0.28)***	0.24 (0.12)*
JIF group: 8	1.61 (0.47)***	0.73 (0.50)	0.96 (0.29)***	0.26 (0.12)*
JIF group: 9	1.55 (0.50)**	1.10 (0.51)*	1.42 (0.30)***	0.25 (0.13)*
JIF group: 10	1.82 (0.48)***	1.19 (0.51)*	1.57 (0.29)***	0.52 (0.12)***
AIC	60688.92	39066.26	42205.72	28749.61
Log Likelihood	-30331.46	-19520.13	-21089.86	-14361.80
N	9137	5938	7622	7708
N groups: Journal ID	1594	1435	1524	1515
N groups: Reviewer ID	8519	5666	7160	7262

***p < 0.001; **p < 0.01; *p < 0.05

Table S3.7: Predicting the prevalence of categories related to 'thoroughness'. Models exclude reviews without mentions of a content category. Table shows coefficients from mixed effects linear regression models. Standard errors in parentheses.

	**Materials and Methods**	**Presentation and Reporting**	**Results and Discussion**	**Importance and Relevance**
(Intercept)	48.49 (0.93)***	39.93 (0.77)***	23.17 (0.63)***	20.69 (0.71)***
JIF group: 2 (ref.: JIF group: 1)	0.06 (1.30)	-3.26 (1.08)**	-1.16 (0.86)	-3.36 (0.99)***
JIF group: 3	0.48 (1.38)	-2.44 (1.13)*	-0.63 (0.90)	-3.47 (1.05)***
JIF group: 4	1.19 (1.39)	-4.54 (1.14)***	-0.47 (0.91)	-4.45 (1.06)***
JIF group: 5	1.72 (1.34)	-4.48 (1.10)***	-1.68 (0.88)	-5.24 (1.01)***
JIF group: 6	3.10 (1.37)*	-5.83 (1.13)***	-3.33 (0.90)***	-5.81 (1.04)***
JIF group: 7	2.92 (1.36)*	-6.15 (1.11)***	-2.18 (0.88)*	-6.37 (1.02)***
JIF group: 8	3.22 (1.41)*	-7.90 (1.15)***	-2.02 (0.90)*	-6.02 (1.04)***
JIF group: 9	4.26 (1.48)**	-8.68 (1.18)***	-2.25 (0.93)*	-6.12 (1.08)***
JIF group: 10	5.35 (1.43)***	-11.38 (1.17)***	-3.27 (0.92)***	-6.58 (1.06)***
AIC	80827.65	76181.64	61421.28	55556.82
BIC	80920.12	76273.59	61511.33	55645.33
Log Likelihood	-40400.83	-38077.82	-30697.64	-27765.41
N	9070	8717	7528	6693
N groups: Reviewer ID	8446	8139	7073	6350
N groups: Journal ID	1582	1582	1496	1475

***$p < 0.001$; **$p < 0.01$; *$p < 0.05$

Table S3.8: Predicting the prevalence of categories related to 'helpfulness'. Models exclude reviews without mentions of a content category. Table shows coefficients from mixed effects linear regression models. Standard errors in parentheses.

	**Suggestion and Solution**	**Example**	**Criticism**	**Praise**
(Intercept)	41.93 (0.74)***	23.20 (0.84)***	24.39 (0.60)***	23.45 (0.88)***
JIF group: 2 (ref.: JIF group: 1)	-2.76 (1.04)**	0.53 (1.15)	-2.04 (0.84)*	-2.60 (1.24)*
JIF group: 3	-1.88 (1.09)	-0.11 (1.20)	-2.05 (0.86)*	-2.43 (1.30)
JIF group: 4	-2.93 (1.10)**	-0.98 (1.22)	-1.32 (0.86)	-4.09 (1.32)**
JIF group: 5	-3.36 (1.07)**	-0.80 (1.17)	-3.88 (0.84)***	-4.92 (1.26)***
JIF group: 6	-4.71 (1.09)***	-0.98 (1.20)	-2.96 (0.85)***	-4.15 (1.29)**
JIF group: 7	-5.53 (1.08)***	-2.31 (1.18)	-3.84 (0.84)***	-6.46 (1.27)***
JIF group: 8	-4.89 (1.11)***	-3.14 (1.22)**	-3.72 (0.85)***	-6.23 (1.30)***
JIF group: 9	-7.44 (1.15)***	-2.18 (1.25)	-2.75 (0.87)**	-6.61 (1.34)***
JIF group: 10	-9.12 (1.13)***	-3.30 (1.23)**	-4.06 (0.86)***	-5.99 (1.33)***
AIC	79480.90	49555.20	62265.66	68584.00
BIC	79573.46	49642.16	62355.86	68674.35
Log Likelihood	-39727.45	-24764.60	-31119.83	-34279.00
N	9137	5938	7622	7708
N groups: Reviewer ID	8519	5666	7160	7262
N groups: Journal ID	1594	1435	1524	1515

***$p < 0.001$; **$p < 0.01$; *$p < 0.05$

Sensitivity analysis 2: Limit sample to reviews with at least 10 sentences (sentence models) or at least 200 words (prevalence models)

In the second sensitivity analysis, only reviews consisting of at least 10 sentences (sentence models) or 200 words (prevalence models) are considered. Figure S3.4 shows the predicted counts of sentences. Figure S3.5 shows percentage point changes in sentences addressing content categories across the JIF Groups (with JIF Group = 1 as the reference category). Tables S3.9–S3.12 show the full regression tables for all models.

Fig S3.3. Predicted number of sentences addressing thoroughness and helpfulness categories across the ten Journal Impact Factor groups.
Predicted values and 95% confidence intervals are shown. Models only include reviews consisting of at least 10 sentences. All negative binomial mixed-effects models include random intercepts for the journal name and reviewer ID. The data underlying this figure can be found in S13 Data.

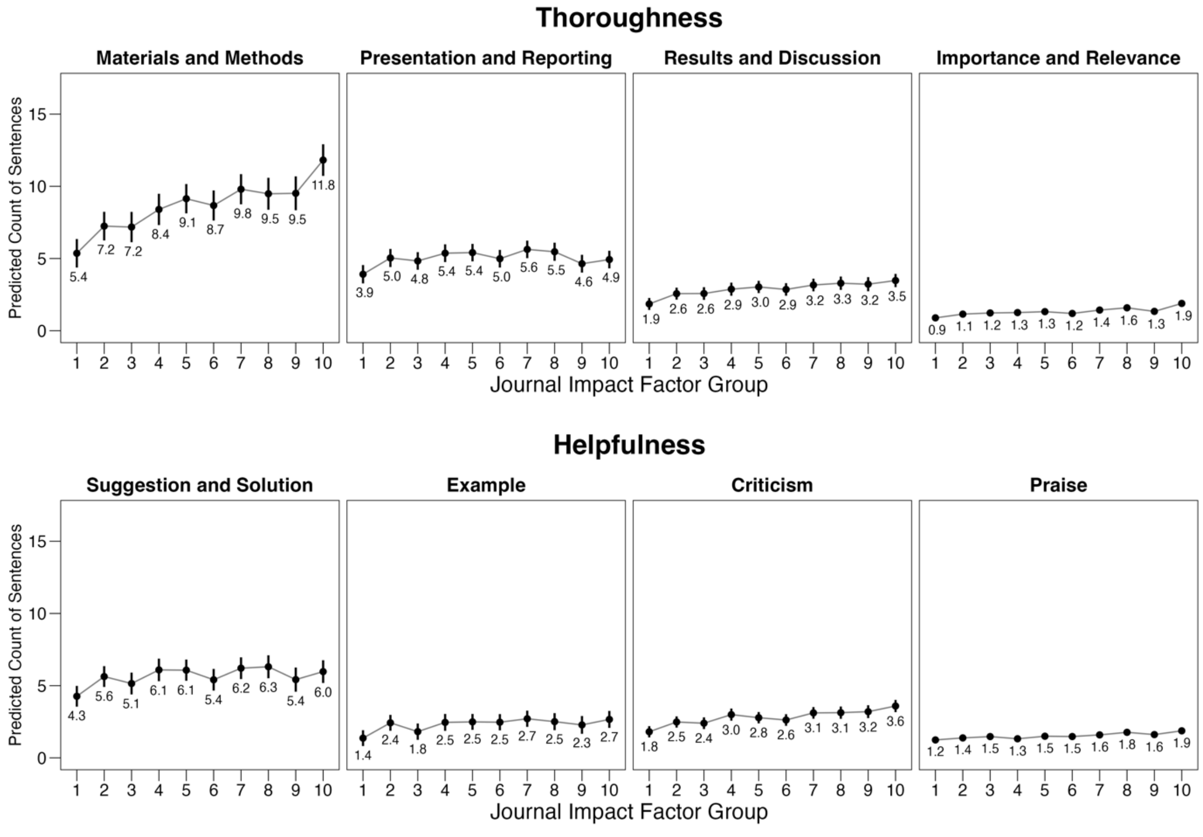

Fig S3.4. Percentage point change in the proportion of sentences addressing thoroughness and helpfulness categories relative to the lowest Journal Impact Factor group.

Regression coefficients and 95% confidence intervals are shown. Models only include reviews consisting of at least 10 sentences. All linear mixed-effects models include random intercepts for the journal name and reviewer ID. The data underlying this figure can be found in S14 Data.

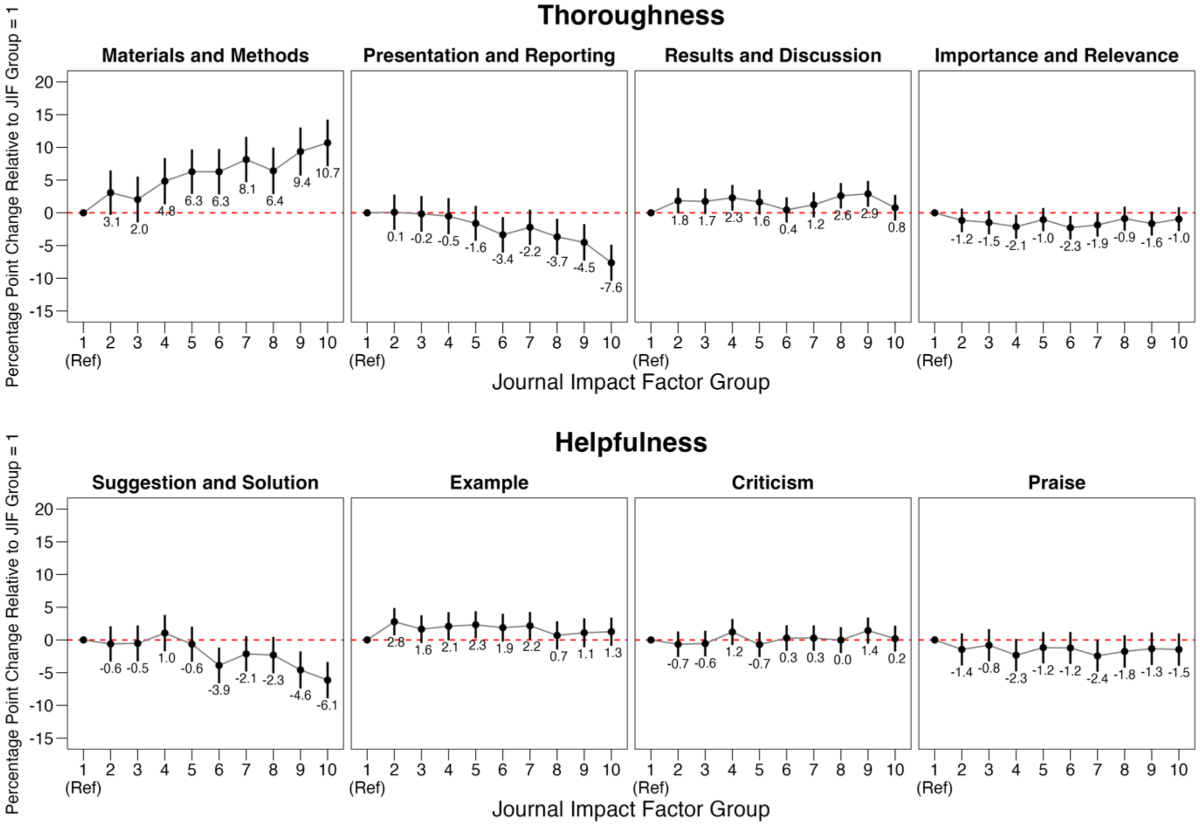

Table S3.9: Predicting the prevalence of categories related to 'thoroughness'. The sample is limited to reviews consisting of at least 10 sentences. Table shows coefficients from negative binomial regression models. Standard errors in parentheses.

	**Materials and Methods**	**Presentation and Reporting**	**Results and Discussion**	**Importance and Relevance**
(Intercept)	$9.88\ (0.49)^{***}$	$7.64\ (0.38)^{***}$	$3.62\ (0.23)^{***}$	$1.92\ (0.12)^{***}$
JIF group: 2 (ref.: JIF group: 1)	$1.47\ (0.67)^{*}$	0.91 (0.52)	0.54 (0.31)	0.10 (0.17)
JIF group: 3	1.31 (0.71)	0.17 (0.55)	0.50 (0.33)	0.17 (0.18)
JIF group: 4	$2.65\ (0.71)^{***}$	0.55 (0.56)	$0.89\ (0.33)^{**}$	0.15 (0.18)
JIF group: 5	$3.09\ (0.69)^{***}$	0.88 (0.53)	$1.05\ (0.32)^{**}$	0.11 (0.18)
JIF group: 6	$2.74\ (0.70)^{***}$	0.28 (0.54)	0.47 (0.33)	-0.10 (0.18)
JIF group: 7	$3.99\ (0.69)^{***}$	0.66 (0.54)	$1.20\ (0.32)^{***}$	0.20 (0.18)
JIF group: 8	$3.11\ (0.70)^{***}$	-0.08 (0.55)	$0.93\ (0.33)^{**}$	0.22 (0.18)
JIF group: 9	$3.72\ (0.73)^{***}$	0.08 (0.57)	$1.05\ (0.34)^{**}$	0.22 (0.19)
JIF group: 10	$5.22\ (0.71)^{***}$	-0.10 (0.55)	$1.08\ (0.33)^{**}$	$0.56\ (0.18)^{**}$
AIC	47758.44	44617.36	37566.60	28476.72
Log Likelihood	-23866.22	-22295.68	-18770.30	-14225.36
N	6510	6510	6510	6510
N groups: Journal ID	1457	1457	1457	1457
N groups: Reviewer ID	6157	6157	6157	6157

$^{***}p < 0.001$; $^{**}p < 0.01$; $^{*}p < 0.05$

Table S3.10: Predicting the prevalence of categories related to 'helpfulness'. The sample is limited to reviews consisting of at least 10 sentences. Table shows coefficients from negative binomial regression models. Standard errors in parentheses.

	**Suggestion and Solution**	**Example**	**Criticism**	**Praise**
(Intercept)	8.18 (0.38)***	3.58 (0.34)***	3.62 (0.22)***	1.88 (0.10)***
JIF group: 2 (ref.: JIF group: 1)	0.91 (0.52)	0.86 (0.47)	0.66 (0.30)*	0.09 (0.14)
JIF group: 3	0.13 (0.55)	0.43 (0.50)	0.43 (0.31)	0.09 (0.15)
JIF group: 4	0.88 (0.55)	0.87 (0.50)	1.02 (0.32)**	-0.10 (0.15)
JIF group: 5	1.03 (0.53)	1.26 (0.48)**	0.84 (0.30)**	0.09 (0.14)
JIF group: 6	0.37 (0.54)	1.11 (0.49)*	0.46 (0.31)	-0.01 (0.14)
JIF group: 7	1.06 (0.53)*	1.00 (0.48)*	1.03 (0.30)***	0.17 (0.14)
JIF group: 8	0.60 (0.54)	0.72 (0.49)	0.79 (0.31)*	0.19 (0.15)
JIF group: 9	0.30 (0.56)	1.19 (0.51)*	1.45 (0.32)***	0.15 (0.15)
JIF group: 10	0.46 (0.55)	1.14 (0.50)*	1.48 (0.31)***	0.41 (0.15)**
AIC	44312.27	42799.77	37043.51	26199.62
Log Likelihood	-22143.13	-21386.88	-18508.76	-13086.81
N	6510	6510	6510	6510
N groups: Journal ID	1457	1457	1457	1457
N groups: Reviewer ID	6157	6157	6157	6157

***$p < 0.001$; **$p < 0.01$; *$p < 0.05$

Table S3.11: Predicting the prevalence of categories related to 'thoroughness'. The sample is limited to reviews longer than 200 words. Table shows coefficients from mixed effects linear regression models. Standard errors in parentheses.

	**Materials and Methods**	**Presentation and Reporting**	**Results and Discussion**	**Importance and Relevance**
(Intercept)	45.04 (1.08)***	32.72 (0.86)***	16.47 (0.60)***	8.75 (0.45)***
JIF group: 2 (ref.: JIF group: 1)	1.80 (1.49)	-0.55 (1.17)	0.48 (0.82)	0.10 (0.62)
JIF group: 3	2.85 (1.60)	-0.56 (1.25)	0.67 (0.86)	0.12 (0.65)
JIF group: 4	4.51 (1.60)**	-1.13 (1.24)	1.52 (0.85)	-0.10 (0.65)
JIF group: 5	4.70 (1.52)**	-1.28 (1.18)	1.26 (0.82)	0.44 (0.62)
JIF group: 6	5.66 (1.56)***	-2.06 (1.21)	-0.03 (0.83)	-0.59 (0.63)
JIF group: 7	5.70 (1.54)***	-2.66 (1.19)*	1.14 (0.82)	-0.09 (0.62)
JIF group: 8	6.31 (1.58)***	-4.68 (1.21)***	1.18 (0.83)	0.30 (0.63)
JIF group: 9	7.14 (1.65)***	-4.29 (1.25)***	1.27 (0.85)	0.45 (0.65)
JIF group: 10	8.94 (1.58)***	-7.35 (1.22)***	0.44 (0.83)	0.91 (0.64)
AIC	55991.03	54031.21	49793.09	45944.96
BIC	56078.95	54119.13	49881.01	46032.88
Log Likelihood	-27982.52	-27002.61	-24883.55	-22959.48
N	6392	6392	6392	6392
N groups: Reviewer ID	6048	6048	6048	6048
N groups: Journal ID	1449	1449	1449	1449

***p < 0.001; **p < 0.01; *p < 0.05

Table S3.12: Predicting the prevalence of categories related to 'helpfulness'. The sample is limited to reviews longer than 200 words. Table shows coefficients from mixed effects linear regression models. Standard errors in parentheses.

	**Suggestion and Solution**	**Example**	**Criticism**	**Praise**
(Intercept)	35.92 (0.77)***	14.12 (0.82)***	15.77 (0.56)***	9.21 (0.41)***
JIF group: 2 (ref.: JIF group: 1)	-0.34 (1.06)	1.65 (1.12)	1.52 (0.76)*	0.00 (0.56)
JIF group: 3	-0.74 (1.11)	1.38 (1.20)	1.50 (0.78)	-0.07 (0.58)
JIF group: 4	-0.75 (1.10)	1.50 (1.19)	2.16 (0.77)**	-1.10 (0.57)
JIF group: 5	-0.86 (1.05)	1.51 (1.14)	0.79 (0.75)	0.08 (0.55)
JIF group: 6	-1.47 (1.07)	2.26 (1.17)	0.73 (0.76)	-0.48 (0.55)
JIF group: 7	-1.93 (1.05)	1.65 (1.15)	1.57 (0.74)*	-0.44 (0.54)
JIF group: 8	-2.15 (1.07)*	0.53 (1.18)	0.55 (0.75)	-0.04 (0.55)
JIF group: 9	-3.92 (1.10)***	2.22 (1.23)	3.17 (0.76)***	0.03 (0.56)
JIF group: 10	-5.34 (1.08)***	0.79 (1.18)	1.77 (0.75)*	0.02 (0.55)
AIC	53136.08	52622.08	49422.42	45443.43
BIC	53224.00	52710.00	49510.34	45531.34
Log Likelihood	-26555.04	-26298.04	-24698.21	-22708.71
N	6392	6392	6392	6392
N groups: Reviewer ID	6048	6048	6048	6048
N groups: Journal ID	1449	1449	1449	1449

***$p < 0.001$; **$p < 0.01$; *$p < 0.05$

Sensitivity analysis 3: Adjust regression models for additional variables (discipline, career stage of reviewers, logged number of reviews submitted)

In the third sensitivity analysis, the regression models were adjusted for additional variables: the discipline, career stage of reviewers, and the logged number of reviews submitted by a review. Note that these reviewer-level metadata are missing for many reviewers, which reduces the number of observations in all regression models. Figure S3.5 shows the predicted counts of sentences. Figure S3.6 shows percentage point changes in sentences addressing content categories across the JIF Groups (with JIF Group = 1 as the reference category). Tables S3.13–S3.16 show the full regression tables for all models. Due to the higher number of coefficients, these tables span two pages.

Fig S3.5. Predicted number of sentences addressing thoroughness and helpfulness categories across the ten Journal Impact Factor groups.
Predicted values and 95% confidence intervals are shown. Models adjust for discipline, career stage of reviewers, logged number of reviews submitted. All negative binomial mixed-effects models include random intercepts for the journal name and reviewer ID. The data underlying this figure can be found in S15 Data.

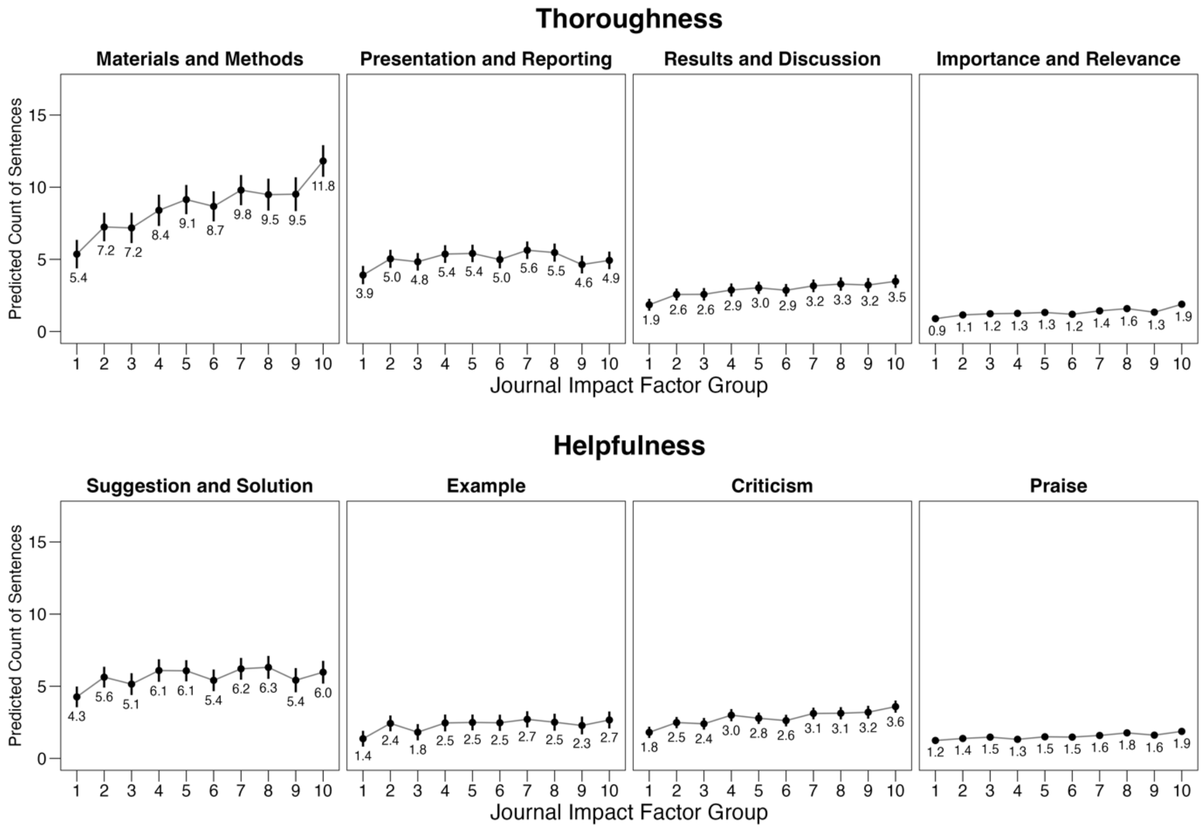

Fig S3.6. Percentage point change in the proportion of sentences addressing thoroughness and helpfulness categories relative to the lowest Journal Impact Factor group.

Regression coefficients and 95% confidence intervals are shown. Models adjust for discipline, career stage of reviewers, logged number of reviews submitted. All linear mixed-effects models include random intercepts for the journal name and reviewer ID. The data underlying this figure can be found in S16 Data.

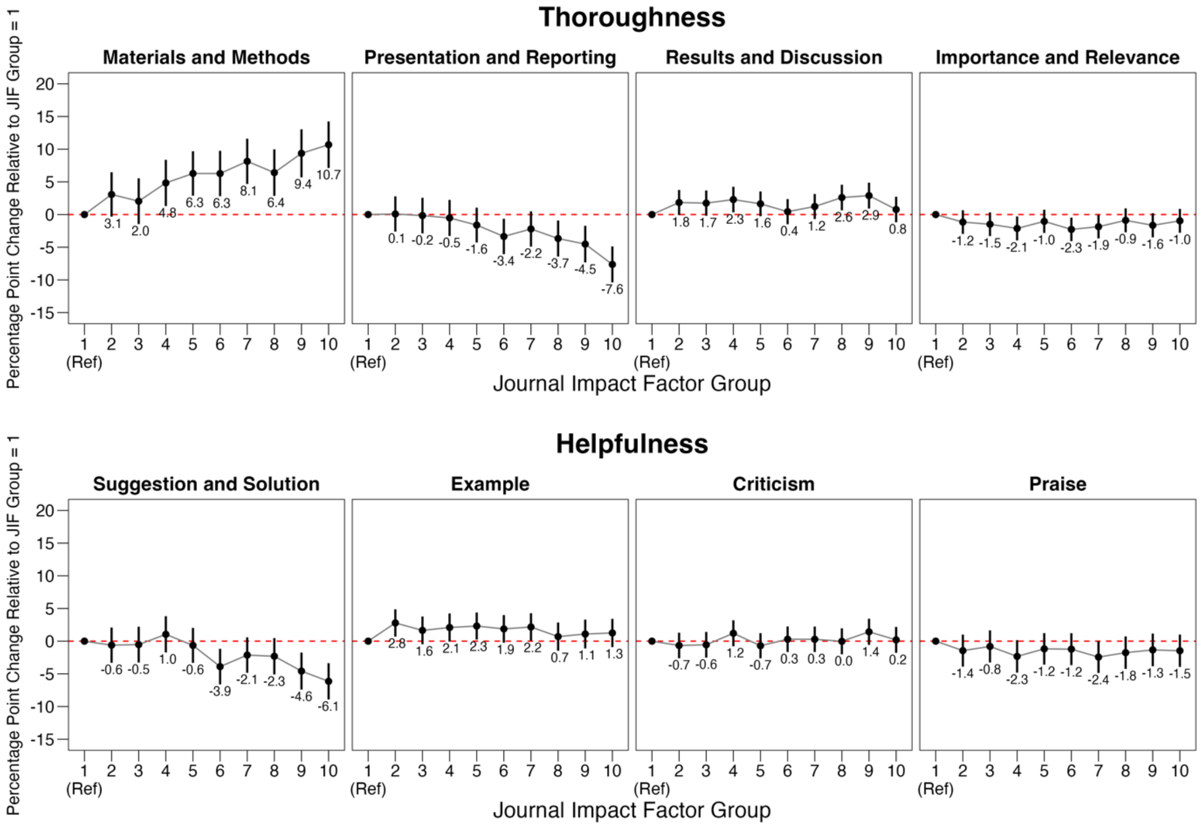

Table S3.13: Predicting the prevalence of categories related to 'thoroughness'. Models adjust for additional variables. Table shows coefficients from negative binomial regression models. Standard errors in parentheses.

	**Materials and Methods**	**Presentation and Reporting**	**Results and Discussion**	**Importance and Relevance**
(Intercept)	6.71 (0.78)***	5.30 (0.49)***	1.91 (0.33)***	1.24 (0.16)***
JIF group: 2 (ref.: JIF group: 1)	1.88 (0.68)**	1.13 (0.44)*	0.71 (0.29)*	0.26 (0.14)
JIF group: 3	1.82 (0.71)*	0.92 (0.43)*	0.72 (0.30)*	0.34 (0.15)*
JIF group: 4	3.04 (0.71)***	1.46 (0.44)***	1.02 (0.30)***	0.37 (0.15)*
JIF group: 5	3.78 (0.69)***	1.50 (0.43)***	1.18 (0.29)***	0.43 (0.14)**
JIF group: 6	3.31 (0.70)***	1.07 (0.43)*	1.00 (0.29)***	0.30 (0.14)*
JIF group: 7	4.44 (0.70)***	1.72 (0.43)***	1.31 (0.29)***	0.54 (0.14)***
JIF group: 8	4.12 (0.72)***	1.56 (0.44)***	1.44 (0.30)***	0.70 (0.15)***
JIF group: 9	4.15 (0.75)***	0.73 (0.43)	1.37 (0.31)***	0.45 (0.15)**
JIF group: 10	6.46 (0.72)***	1.02 (0.43)*	1.62 (0.30)***	1.00 (0.15)***
Reviewer's academic age	-0.00 (0.00)	-0.00 (0.00)	-0.00 (0.00)	-0.00 (0.00)
N reviews by reviewer (log)	-0.19 (0.09)*	-0.11 (0.07)	-0.02 (0.04)	-0.03 (0.02)
Clinical Medicine (ref.: Biology and Biochemistry)	-0.56 (0.56)	-0.96 (0.32)**	0.03 (0.23)	-0.22 (0.12)
Environment and Ecology	2.70 (0.72)***	3.15 (0.39)***	1.93 (0.30)***	1.05 (0.15)***
Immunology	0.39 (1.02)	0.02 (0.59)	0.06 (0.43)	-0.19 (0.21)
Microbiology	1.79 (1.08)	2.63 (0.63)***	0.16 (0.45)	0.25 (0.22)
Mol. Biology and Genetics	0.86 (0.80)	0.82 (0.45)	-0.02 (0.33)	-0.09 (0.16)
Neuroscience and Behavior	3.74 (0.81)***	1.09 (0.47)*	2.28 (0.34)***	0.29 (0.17)
Pharmacology and Toxicology	-0.75 (0.79)	-0.39 (0.45)	-0.30 (0.33)	-0.32 (0.16)*
Psychiatry and Psychology	2.73 (0.74)***	1.87 (0.45)***	2.37 (0.31)***	1.63 (0.15)***
AIC	42312.35		32297.64	23937.17

Table S3.13: Predicting the prevalence of categories related to 'thoroughness'. Models adjust for additional variables. Table shows coefficients from negative binomial regression models. Standard errors in parentheses.

	**Materials and Methods**	**Presentation and Reporting**	**Results and Discussion**	**Importance and Relevance**
Log Likelihood	-21133.17		-16125.82	-11945.58
N	5806	5806	5806	5806
N groups: Journal ID	1458	1458	1458	1458
N groups: Reviewer ID	5329	5329	5329	5329

$^{***}p < 0.001$; $^{**}p < 0.01$; $^{*}p < 0.05$

Table S3.14: Predicting the prevalence of categories related to 'helpfulness'. Models adjust for additional variables. Table shows coefficients from negative binomial regression models. Standard errors in parentheses.

	**Suggestion and Solution**	**Example**	**Criticism**	**Praise**
(Intercept)	5.49 (0.57)***	3.22 (0.43)***	2.30 (0.31)***	1.50 (0.14)***
JIF group: 2 (ref.: JIF group: 1)	1.37 (0.50)**	1.06 (0.38)**	0.68 (0.27)*	0.14 (0.12)
JIF group: 3	0.88 (0.51)	0.45 (0.39)	0.59 (0.28)*	0.23 (0.13)
JIF group: 4	1.83 (0.52)***	1.09 (0.39)**	1.19 (0.28)***	0.08 (0.13)
JIF group: 5	1.81 (0.50)***	1.13 (0.38)**	0.97 (0.27)***	0.26 (0.12)*
JIF group: 6	1.14 (0.51)*	1.10 (0.38)**	0.81 (0.27)**	0.24 (0.13)
JIF group: 7	1.95 (0.51)***	1.34 (0.38)***	1.31 (0.27)***	0.35 (0.13)**
JIF group: 8	2.04 (0.52)***	1.14 (0.39)**	1.32 (0.28)***	0.53 (0.13)***
JIF group: 9	1.16 (0.54)*	0.92 (0.40)*	1.39 (0.29)***	0.37 (0.14)**
JIF group: 10	1.71 (0.52)**	1.30 (0.39)***	1.78 (0.28)***	0.63 (0.13)***
Reviewer's academic age	-0.00 (0.00)	-0.00 (0.00)	-0.00 (0.00)	-0.00 (0.00)
N reviews by reviewer (log)	-0.11 (0.07)	-0.21 (0.06)***	-0.03 (0.04)	-0.02 (0.02)
Clinical Medicine (ref.: Biology and Biochemistry)	-0.79 (0.40)	-1.02 (0.30)***	-0.38 (0.22)	-0.17 (0.10)
Environment and Ecology	4.13 (0.52)***	3.85 (0.39)***	2.16 (0.28)***	0.71 (0.13)***
Immunology	-0.41 (0.74)	0.14 (0.55)	-0.05 (0.40)	-0.17 (0.18)
Microbiology	2.55 (0.78)**	2.47 (0.59)***	0.49 (0.42)	0.39 (0.19)*
Mol. Biology and Genetics	0.67 (0.58)	0.69 (0.43)	0.52 (0.31)	0.06 (0.14)
Neuroscience and Behavior	1.73 (0.58)**	0.25 (0.44)	0.72 (0.31)*	0.26 (0.15)
Pharmacology and Toxicology	-0.48 (0.57)	-0.51 (0.43)	-0.27 (0.31)	-0.33 (0.14)*
Psychiatry and Psychology	2.50 (0.54)***	-0.43 (0.41)	1.50 (0.29)***	0.84 (0.13)***
AIC	39039.62	36231.62	32007.67	22164.70
Log Likelihood	-19496.81	-18092.81	-15980.84	-11059.35

Table S3.14: Predicting the prevalence of categories related to 'helpfulness'. Models adjust for additional variables. Table shows coefficients from negative binomial regression models. Standard errors in parentheses.

	**Suggestion and Solution**	**Example**	**Criticism**	**Praise**
N	5806	5806	5806	5806
N groups: Journal ID	1458	1458	1458	1458
N groups: Reviewer ID	5329	5329	5329	5329

$^{***}p < 0.001$; $^{**}p < 0.01$; $^{*}p < 0.05$

Table S3.15: Predicting the prevalence of categories related to 'thoroughness'. Models adjust for additional variables. Table shows coefficients from mixed effects linear regression models. Standard errors in parentheses.

	**Materials and Methods**	**Presentation and Reporting**	**Results and Discussion**	**Importance and Relevance**
(Intercept)	41.92 (1.98)***	34.98 (1.56)***	11.81 (1.11)***	12.29 (1.04)***
JIF group: 2 (ref.: JIF group: 1)	3.07 (1.73)	0.09 (1.38)	1.84 (0.98)	-1.15 (0.92)
JIF group: 3	2.04 (1.79)	-0.16 (1.39)	1.75 (0.99)	-1.48 (0.92)
JIF group: 4	4.84 (1.81)**	-0.52 (1.41)	2.29 (1.00)*	-2.13 (0.94)*
JIF group: 5	6.28 (1.73)***	-1.61 (1.36)	1.64 (0.97)	-1.04 (0.91)
JIF group: 6	6.28 (1.77)***	-3.36 (1.38)*	0.44 (0.98)	-2.27 (0.92)*
JIF group: 7	8.14 (1.77)***	-2.21 (1.38)	1.23 (0.98)	-1.86 (0.92)*
JIF group: 8	6.42 (1.82)***	-3.67 (1.41)**	2.60 (1.00)**	-0.89 (0.93)
JIF group: 9	9.37 (1.87)***	-4.52 (1.43)**	2.89 (1.01)**	-1.65 (0.94)
JIF group: 10	10.69 (1.82)***	-7.64 (1.40)***	0.77 (1.00)	-0.96 (0.93)
Reviewer's academic age	0.00 (0.00)	-0.00 (0.00)	0.01 (0.00)**	-0.00 (0.00)
N reviews by reviewer (log)	-0.14 (0.24)	-0.49 (0.20)*	0.26 (0.15)	-0.22 (0.14)
Clinical Medicine (ref.: Biology and Biochemistry)	1.45 (1.41)	-1.45 (1.07)	1.93 (0.76)*	0.58 (0.70)
Environment and Ecology	-7.91 (1.80)***	1.66 (1.36)	1.86 (0.95)	1.47 (0.89)
Immunology	2.05 (2.56)	0.83 (1.97)	1.11 (1.39)	-0.59 (1.30)
Microbiology	-5.86 (2.71)*	4.38 (2.09)*	-2.91 (1.48)*	-1.81 (1.38)
Mol. Biology and Genetics	-0.59 (2.00)	1.98 (1.52)	-1.06 (1.08)	-1.75 (1.00)
Neuroscience and Behavior	2.87 (2.03)	-1.08 (1.55)	6.00 (1.10)***	0.15 (1.03)
Pharmacology and Toxicology	-0.64 (1.99)	4.55 (1.51)**	-0.16 (1.07)	-1.36 (1.00)
Psychiatry and Psychology	-4.74 (1.88)*	-3.31 (1.46)*	4.40 (1.04)***	3.94 (0.97)***
AIC	53318.74	51470.09	47708.60	47084.78

Table S3.15: Predicting the prevalence of categories related to 'thoroughness'. Models adjust for additional variables. Table shows coefficients from mixed effects linear regression models. Standard errors in parentheses.

	**Materials and Methods**	**Presentation and Reporting**	**Results and Discussion**	**Importance and Relevance**
BIC	53472.07	51623.42	47861.93	47238.11
Log Likelihood	-26636.37	-25712.05	-23831.30	-23519.39
N	5806	5806	5806	5806
N groups: Reviewer ID	5329	5329	5329	5329
N groups: Journal ID	1458	1458	1458	1458

$^{***}p < 0.001$; $^{**}p < 0.01$; $^{*}p < 0.05$

Table S3.16: Predicting the prevalence of categories related to 'helpfulness'. Models adjust for additional variables. Table shows coefficients from mixed effects linear regression models. Standard errors in parentheses.

	**Suggestion and Solution**	**Example**	**Criticism**	**Praise**
(Intercept)	38.12 (1.57)***	16.85 (1.22)***	15.88 (1.13)***	15.63 (1.41)***
JIF group: 2 (ref.: JIF group: 1)	-0.61 (1.38)	2.79 (1.07)**	-0.66 (1.00)	-1.45 (1.25)
JIF group: 3	-0.53 (1.40)	1.65 (1.08)	-0.55 (1.00)	-0.81 (1.25)
JIF group: 4	1.05 (1.42)	2.09 (1.10)	1.21 (1.02)	-2.33 (1.27)
JIF group: 5	-0.65 (1.36)	2.31 (1.06)*	-0.70 (0.98)	-1.18 (1.23)
JIF group: 6	-3.90 (1.39)**	1.88 (1.08)	0.29 (1.00)	-1.22 (1.25)
JIF group: 7	-2.13 (1.38)	2.17 (1.07)*	0.29 (0.99)	-2.43 (1.24)
JIF group: 8	-2.31 (1.42)	0.70 (1.10)	-0.02 (1.01)	-1.76 (1.27)
JIF group: 9	-4.58 (1.44)**	1.09 (1.13)	1.43 (1.02)	-1.34 (1.28)
JIF group: 10	-6.14 (1.42)***	1.26 (1.10)	0.20 (1.01)	-1.46 (1.26)
Reviewer's academic age	0.00 (0.00)	-0.00 (0.00)	-0.00 (0.00)	-0.00 (0.00)
N reviews by reviewer (log)	-0.32 (0.20)	-0.80 (0.16)***	0.20 (0.15)	-0.21 (0.19)
Clinical Medicine (ref.: Biology and Biochemistry)	-0.90 (1.08)	-4.46 (0.85)***	-0.32 (0.76)	1.42 (0.95)
Environment and Ecology	1.78 (1.38)	4.85 (1.08)***	1.48 (0.96)	0.28 (1.20)
Immunology	-0.31 (1.99)	-0.86 (1.55)	-0.70 (1.41)	0.25 (1.76)
Microbiology	2.88 (2.11)	6.53 (1.65)***	-2.47 (1.50)	-1.09 (1.87)
Mol. Biology and Genetics	0.61 (1.54)	1.05 (1.20)	0.09 (1.09)	-0.22 (1.36)
Neuroscience and Behavior	-1.07 (1.57)	-2.35 (1.22)	-1.21 (1.12)	-0.69 (1.39)
Pharmacology and Toxicology	2.43 (1.53)	0.20 (1.19)	0.45 (1.08)	-0.84 (1.35)
Psychiatry and Psychology	-1.51 (1.47)	-6.24 (1.15)***	0.39 (1.05)	2.63 (1.31)*
AIC	51295.63	48149.04	47987.42	50637.66

Table S3.16: Predicting the prevalence of categories related to 'helpfulness'. Models adjust for additional variables. Table shows coefficients from mixed effects linear regression models. Standard errors in parentheses.

	**Suggestion and Solution**	**Example**	**Criticism**	**Praise**
BIC	51448.97	48302.37	48140.75	50791.00
Log Likelihood	-25624.82	-24051.52	-23970.71	-25295.83
N	5806	5806	5806	5806
N groups: Reviewer ID	5329	5329	5329	5329
N groups: Journal ID	1458	1458	1458	1458

$^{***}p < 0.001$; $^{**}p < 0.01$; $^{*}p < 0.05$

Sensitivity analysis 4: Compare coefficients for models based on male and female reviewers

In the fourth sensitivity analysis, separate models for male and female reviewers were run. Note that the gender was detected automatically, based on the first name of reviewers. The categories `female` and `mostly_female` were recoded into “female reviewer”. The categories `male` and `mostly_male` were recoded into “male reviewer”. The category `unknown` was recoded as missing and is not considered in the regression models. This automated approach is highly problematic from ethical reasons and error prone. Therefore, the results need to be interpreted with caution. Figure S3.7 shows the predicted counts of sentences for reviewers classified as female. Figure 3.8 shows the predicted counts for reviewers classified as male. Figure 3.9 shows percentage point changes in sentences addressing content categories across the JIF Groups (with JIF Group = 1 as the reference category) classified as female. Figure 3.10 shows percentage point changes in sentences addressing content categories across the JIF Groups for reviewers classified as male. Tables S3.17–S3.24 show the corresponding regression tables.

Fig S3.7. Predicted number of sentences addressing thoroughness and helpfulness categories across the ten Journal Impact Factor groups.
Predicted values and 95% confidence intervals are shown. Models are limited to reviews submitted by scholars classified as “female”. All negative binomial mixed-effects models include random intercepts for the journal name and reviewer ID. The data underlying this figure can be found in S17 Data.

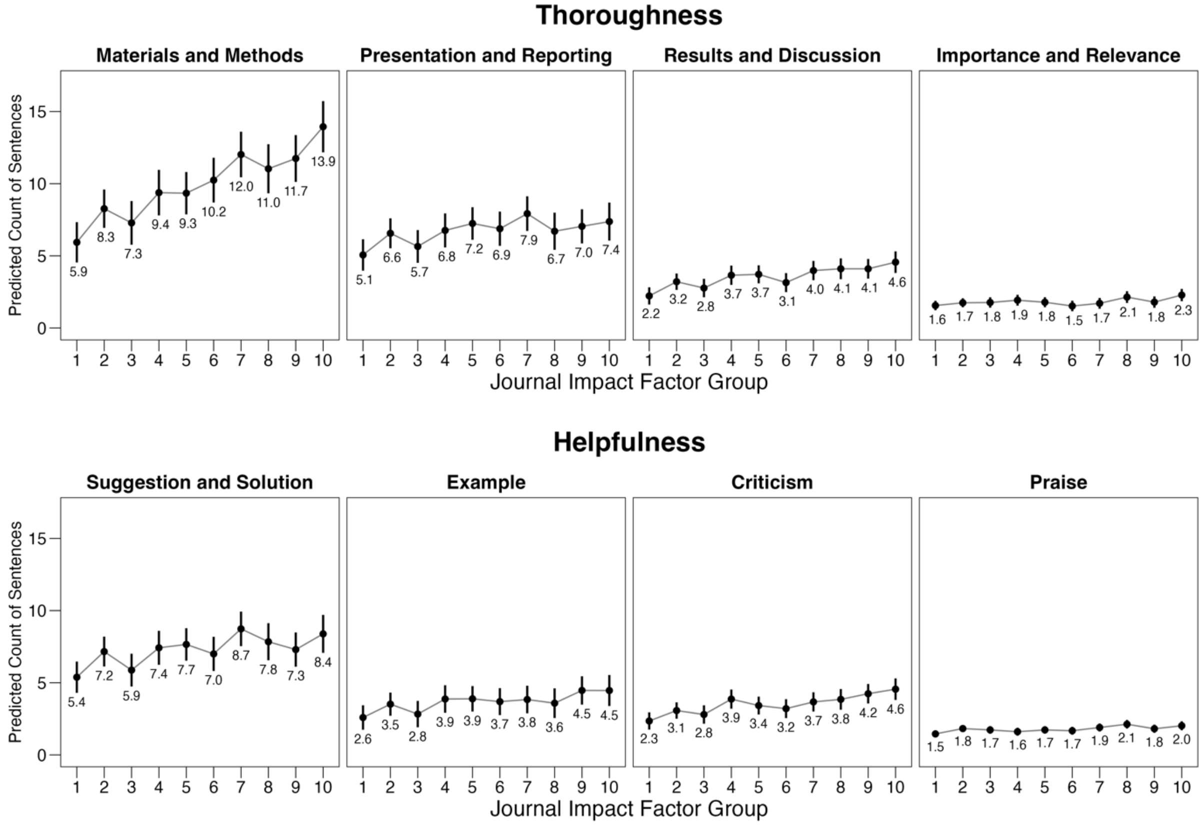

Fig S3.8. Predicted number of sentences addressing thoroughness and helpfulness categories across the ten Journal Impact Factor groups.
Predicted values and 95% confidence intervals are shown. Models are limited to reviews submitted by scholars classified as “male”. All negative binomial mixed-effects models include random intercepts for the journal name and reviewer ID. The data underlying this figure can be found in S18 Data.

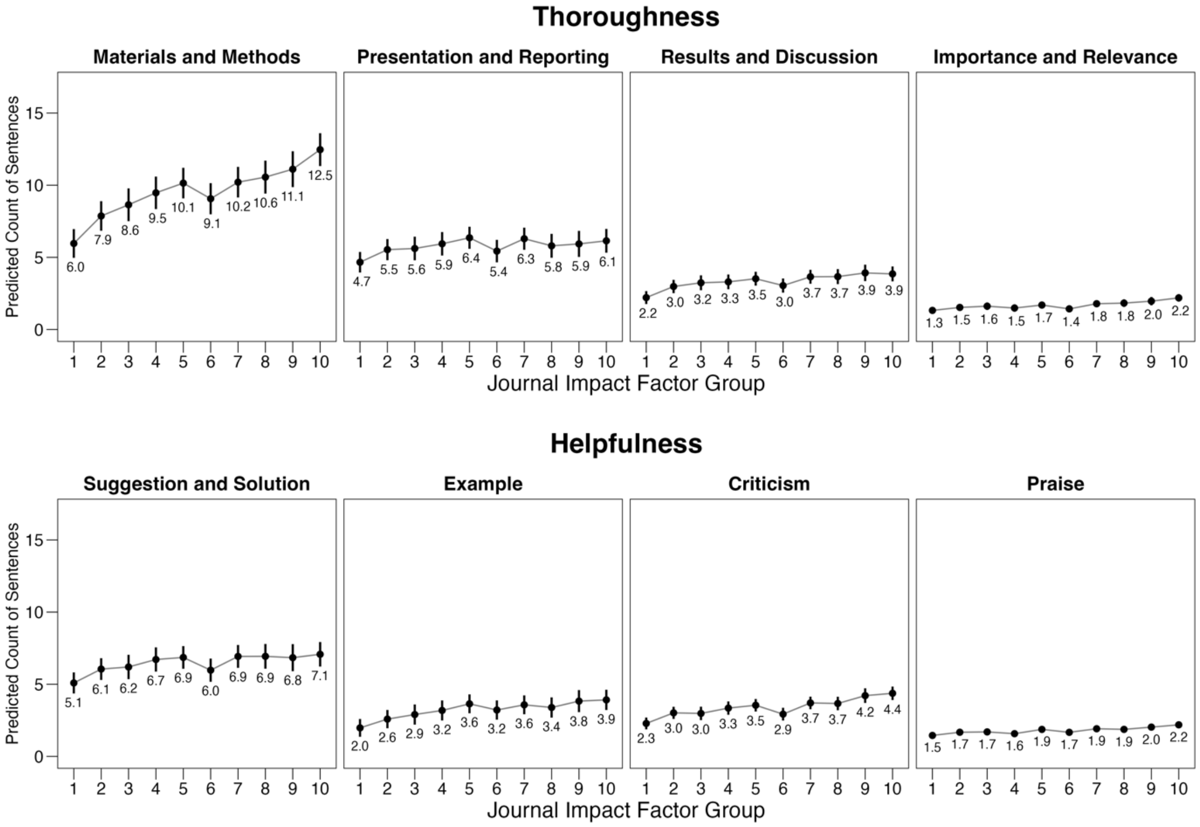

Fig S3.9. Percentage point change in the proportion of sentences addressing thoroughness and helpfulness categories relative to the lowest Journal Impact Factor group.

Regression coefficients and 95% confidence intervals are shown. Models are limited to reviews submitted by scholars classified as “female”. All linear mixed-effects models include random intercepts for the journal name and reviewer ID. The data underlying this figure can be found in S19 Data.

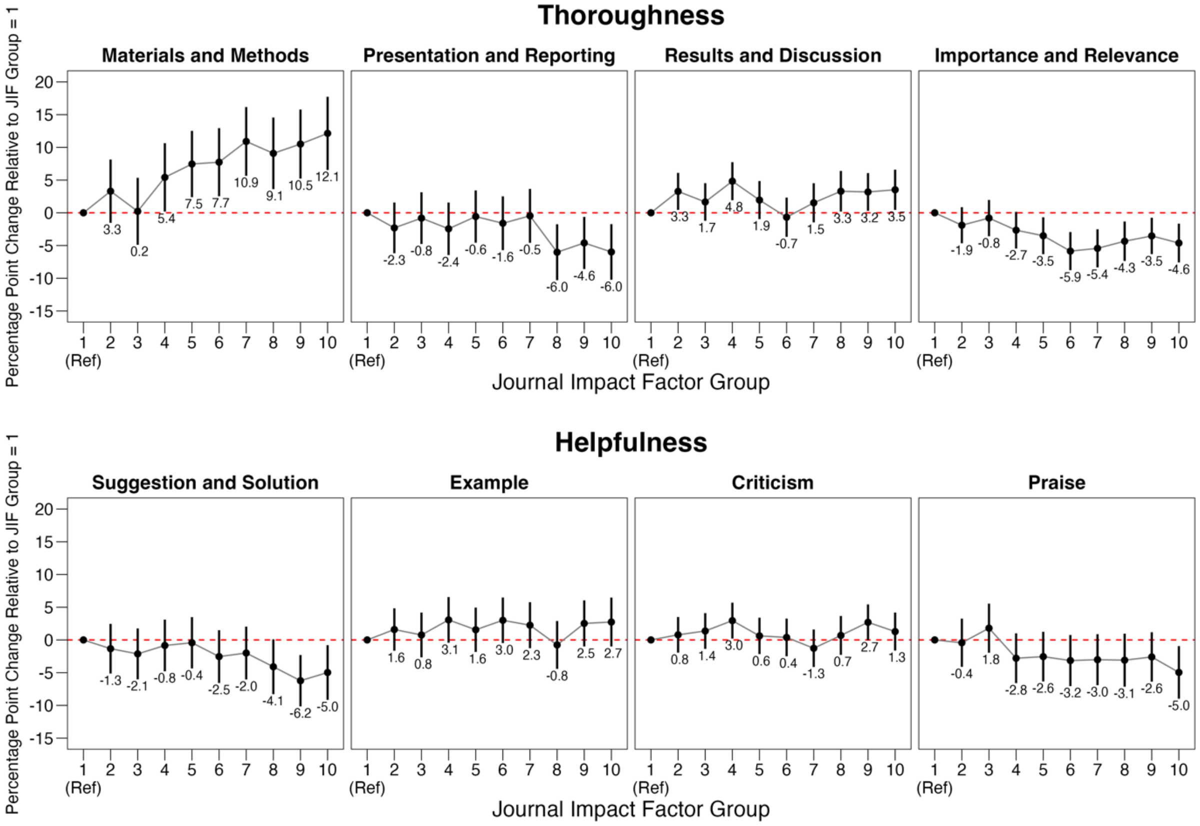

Fig S3.10. Percentage point change in the proportion of sentences addressing thoroughness and helpfulness categories relative to the lowest Journal Impact Factor group.

Regression coefficients and 95% confidence intervals are shown. Models are limited to reviews submitted by scholars classified as "male". All linear mixed-effects models include random intercepts for the journal name and reviewer ID. The data underlying this figure can be found in S20 Data.

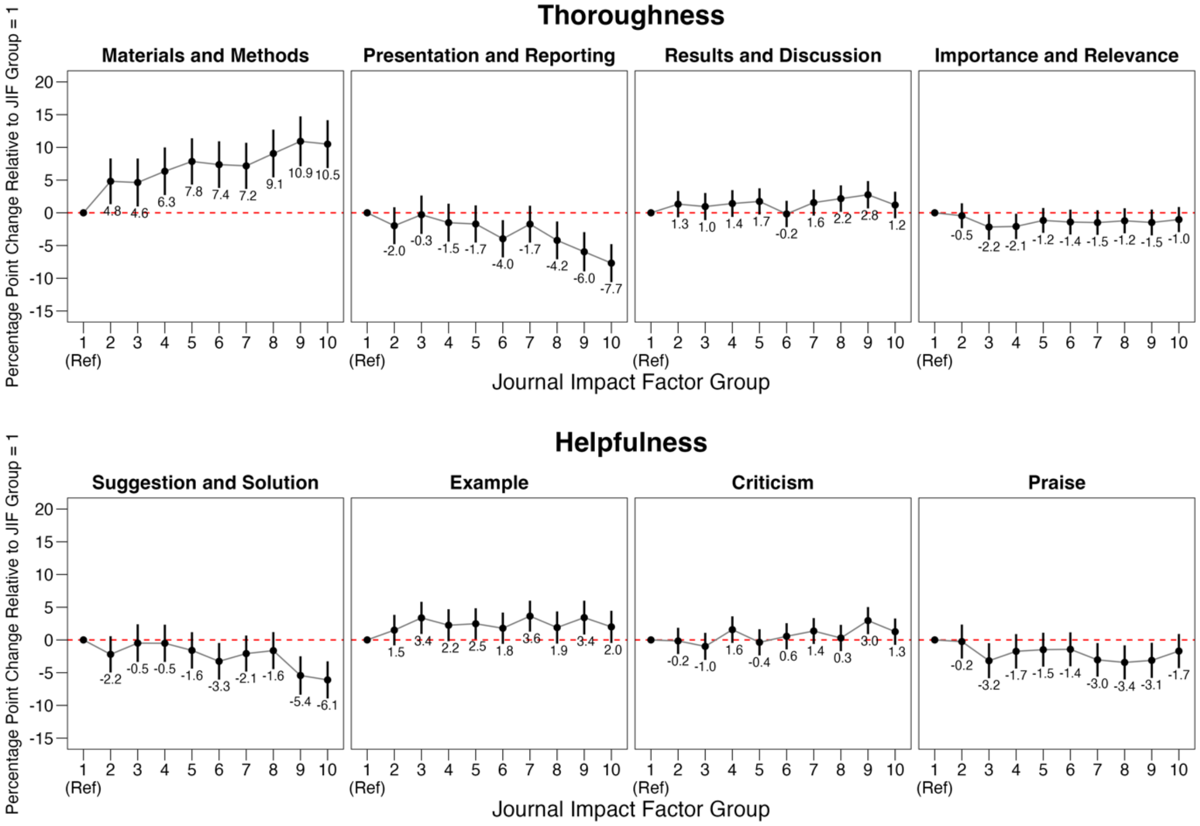

Table S3.17: Predicting the prevalence of categories related to 'thoroughness'. Sample is limited to reviewers classified as 'female'. Table shows coefficients from negative binomial regression models. Standard errors in parentheses.

	**Materials and Methods**	**Presentation and Reporting**	**Results and Discussion**	**Importance and Relevance**
(Intercept)	5.94 (0.71)***	5.06 (0.55)***	2.22 (0.30)***	1.56 (0.17)***
JIF group: 2 (ref.: JIF group: 1)	2.33 (0.98)*	1.50 (0.77)	0.98 (0.42)*	0.19 (0.23)
JIF group: 3	1.35 (1.05)	0.59 (0.80)	0.55 (0.45)	0.21 (0.25)
JIF group: 4	3.44 (1.08)**	1.70 (0.82)*	1.43 (0.46)**	0.37 (0.25)
JIF group: 5	3.40 (1.03)***	2.18 (0.80)**	1.49 (0.44)***	0.23 (0.24)
JIF group: 6	4.31 (1.06)***	1.82 (0.82)*	0.92 (0.45)*	-0.03 (0.25)
JIF group: 7	6.09 (1.08)***	2.86 (0.83)***	1.76 (0.46)***	0.15 (0.25)
JIF group: 8	5.10 (1.12)***	1.64 (0.86)	1.88 (0.48)***	0.59 (0.27)*
JIF group: 9	5.81 (1.09)***	1.98 (0.82)*	1.88 (0.46)***	0.24 (0.26)
JIF group: 10	8.01 (1.15)***	2.31 (0.87)**	2.34 (0.49)***	0.73 (0.27)**
AIC	17223.70	16353.82	13320.72	10183.30
Log Likelihood	-8598.85	-8163.91	-6647.36	-5078.65
N	2342	2342	2342	2342
N groups: Journal ID	858	858	858	858
N groups: Reviewer ID	2213	2213	2213	2213

***$p < 0.001$; **$p < 0.01$; *$p < 0.05$

Table S3.18: Predicting the prevalence of categories related to 'thoroughness'. Sample is limited to reviewers classified as 'male'. Table shows coefficients from negative binomial regression models. Standard errors in parentheses.

	**Materials and Methods**	**Presentation and Reporting**	**Results and Discussion**	**Importance and Relevance**
(Intercept)	5.96 (0.51)***	4.66 (0.36)***	2.21 (0.23)***	1.32 (0.11)***
JIF group: 2 (ref.: JIF group: 1)	1.91 (0.73)**	0.87 (0.52)	0.77 (0.33)*	0.21 (0.16)
JIF group: 3	2.68 (0.77)***	0.95 (0.55)	1.03 (0.35)**	0.29 (0.17)
JIF group: 4	3.51 (0.77)***	1.28 (0.55)*	1.09 (0.35)**	0.16 (0.17)
JIF group: 5	4.18 (0.74)***	1.69 (0.53)**	1.31 (0.33)***	0.37 (0.17)*
JIF group: 6	3.10 (0.75)***	0.77 (0.54)	0.83 (0.34)*	0.11 (0.17)
JIF group: 7	4.25 (0.74)***	1.63 (0.53)**	1.44 (0.34)***	0.46 (0.17)**
JIF group: 8	4.60 (0.77)***	1.14 (0.56)*	1.45 (0.35)***	0.51 (0.18)**
JIF group: 9	5.15 (0.81)***	1.27 (0.59)*	1.71 (0.37)***	0.64 (0.19)***
JIF group: 10	6.50 (0.77)***	1.48 (0.55)**	1.64 (0.35)***	0.87 (0.17)***
AIC	39984.74	36018.57	30885.38	22569.44
Log Likelihood	-19979.37	-17996.28	-15429.69	-11271.72
N	5461	5461	5461	5461
N groups: Journal ID	1324	1324	1324	1324
N groups: Reviewer ID	4972	4972	4972	4972

***$p < 0.001$; **$p < 0.01$; *$p < 0.05$

Table S3.19: Predicting the prevalence of categories related to 'helpfulness'. Sample is limited to reviewers classified as 'female'. Table shows coefficients from negative binomial regression models. Standard errors in parentheses.

	**Suggestion and Solution**	**Example**	**Criticism**	**Praise**
(Intercept)	5.38 (0.55)***	2.59 (0.43)***	2.35 (0.30)***	1.45 (0.13)***
JIF group: 2 (ref.: JIF group: 1)	1.78 (0.76)*	0.93 (0.59)	0.72 (0.42)	0.37 (0.17)*
JIF group: 3	0.49 (0.80)	0.24 (0.63)	0.45 (0.44)	0.27 (0.18)
JIF group: 4	2.04 (0.82)*	1.28 (0.65)*	1.52 (0.45)***	0.15 (0.19)
JIF group: 5	2.27 (0.80)**	1.30 (0.62)*	1.07 (0.44)*	0.27 (0.18)
JIF group: 6	1.61 (0.82)*	1.10 (0.64)	0.85 (0.45)	0.22 (0.19)
JIF group: 7	3.35 (0.82)***	1.25 (0.65)	1.32 (0.46)**	0.44 (0.19)*
JIF group: 8	2.46 (0.86)**	0.99 (0.68)	1.50 (0.47)**	0.67 (0.20)***
JIF group: 9	1.92 (0.82)*	1.88 (0.66)**	1.89 (0.46)***	0.36 (0.19)
JIF group: 10	3.00 (0.87)***	1.87 (0.70)**	2.20 (0.49)***	0.56 (0.20)**
AIC	16329.10	14825.02	13321.69	9124.71
Log Likelihood	-8151.55	-7399.51	-6647.84	-4549.35
N	2342	2342	2342	2342
N groups: Journal ID	858	858	858	858
N groups: Reviewer ID	2213	2213	2213	2213

***$p < 0.001$; **$p < 0.01$; *$p < 0.05$

Table S3.20: Predicting the prevalence of categories related to 'helpfulness'. Sample is limited to reviewers classified as 'male'. Table shows coefficients from negative binomial regression models. Standard errors in parentheses.

	**Suggestion and Solution**	**Example**	**Criticism**	**Praise**
(Intercept)	5.09 (0.37)***	1.98 (0.31)***	2.28 (0.21)***	1.45 (0.09)***
JIF group: 2 (ref.: JIF group: 1)	0.96 (0.53)	0.61 (0.45)	0.73 (0.30)*	0.22 (0.14)
JIF group: 3	1.11 (0.57)	0.92 (0.48)	0.69 (0.31)*	0.24 (0.14)
JIF group: 4	1.63 (0.57)**	1.21 (0.47)*	1.06 (0.31)***	0.12 (0.14)
JIF group: 5	1.77 (0.55)**	1.67 (0.46)***	1.26 (0.30)***	0.42 (0.14)**
JIF group: 6	0.88 (0.55)	1.24 (0.46)**	0.64 (0.31)*	0.22 (0.14)
JIF group: 7	1.84 (0.55)***	1.60 (0.46)***	1.42 (0.30)***	0.46 (0.14)***
JIF group: 8	1.84 (0.57)**	1.41 (0.48)**	1.38 (0.32)***	0.42 (0.15)**
JIF group: 9	1.75 (0.61)**	1.86 (0.50)***	1.93 (0.33)***	0.58 (0.15)***
JIF group: 10	1.99 (0.57)***	1.94 (0.48)***	2.09 (0.32)***	0.74 (0.15)***
AIC	36037.58	34902.79	30230.31	21076.87
Log Likelihood	-18005.79	-17438.40	-15102.16	-10525.43
N	5461	5461	5461	5461
N groups: Journal ID	1324	1324	1324	1324
N groups: Reviewer ID	4972	4972	4972	4972

***$p < 0.001$; **$p < 0.01$; *$p < 0.05$

Table S3.21: Predicting the prevalence of categories related to 'thoroughness'. Sample is limited to reviewers classified as 'female'. Table shows coefficients from mixed effects linear regression models. Standard errors in parentheses.

	**Materials and Methods**	**Presentation and Reporting**	**Results and Discussion**	**Importance and Relevance**
(Intercept)	39.37 (1.79)***	34.95 (1.43)***	14.37 (1.04)***	14.65 (1.02)***
JIF group: 2 (ref.: JIF group: 1)	3.30 (2.47)	-2.31 (1.98)	3.27 (1.44)*	-1.90 (1.41)
JIF group: 3	0.23 (2.61)	-0.81 (2.02)	1.65 (1.47)	-0.81 (1.41)
JIF group: 4	5.41 (2.67)*	-2.44 (2.05)	4.83 (1.48)**	-2.66 (1.43)
JIF group: 5	7.46 (2.58)**	-0.57 (2.04)	1.94 (1.48)	-3.51 (1.44)*
JIF group: 6	7.73 (2.65)**	-1.60 (2.10)	-0.68 (1.53)	-5.86 (1.49)***
JIF group: 7	10.91 (2.68)***	-0.46 (2.10)	1.53 (1.53)	-5.43 (1.49)***
JIF group: 8	9.09 (2.79)**	-6.02 (2.18)**	3.29 (1.58)*	-4.34 (1.54)**
JIF group: 9	10.51 (2.69)***	-4.61 (2.03)*	3.19 (1.47)*	-3.53 (1.42)*
JIF group: 10	12.14 (2.85)***	-5.98 (2.17)**	3.52 (1.57)*	-4.62 (1.51)**
AIC	21642.06	21016.95	19593.41	19526.28
BIC	21716.93	21091.81	19668.28	19601.15
Log Likelihood	-10808.03	-10495.48	-9783.71	-9750.14
N	2342	2342	2342	2342
N groups: Reviewer ID	2213	2213	2213	2213
N groups: Journal ID	858	858	858	858

***p < 0.001; **p < 0.01; *p < 0.05

Table S3.22: Predicting the prevalence of categories related to 'thoroughness'. Sample is limited to reviewers classified as 'male'. Table shows coefficients from mixed effects linear regression models. Standard errors in parentheses.

	**Materials and Methods**	**Presentation and Reporting**	**Results and Discussion**	**Importance and Relevance**
(Intercept)	39.68 (1.25)***	31.69 (1.01)***	14.77 (0.72)***	12.06 (0.68)***
JIF group: 2 (ref.: JIF group: 1)	4.81 (1.78)**	-1.97 (1.44)	1.32 (1.02)	-0.45 (0.97)
JIF group: 3	4.63 (1.87)*	-0.29 (1.50)	0.96 (1.06)	-2.17 (1.00)*
JIF group: 4	6.35 (1.86)***	-1.51 (1.48)	1.42 (1.04)	-2.08 (0.99)*
JIF group: 5	7.85 (1.81)***	-1.71 (1.45)	1.74 (1.02)	-1.17 (0.97)
JIF group: 6	7.36 (1.82)***	-3.97 (1.45)**	-0.17 (1.03)	-1.41 (0.97)
JIF group: 7	7.17 (1.80)***	-1.74 (1.44)	1.56 (1.01)	-1.49 (0.96)
JIF group: 8	9.06 (1.86)***	-4.21 (1.47)**	2.17 (1.03)*	-1.22 (0.98)
JIF group: 9	10.92 (1.95)***	-5.96 (1.53)***	2.76 (1.07)**	-1.48 (1.01)
JIF group: 10	10.50 (1.87)***	-7.69 (1.48)***	1.19 (1.04)	-1.03 (0.98)
AIC	50442.69	48724.81	45354.26	44857.20
BIC	50528.56	48810.68	45440.13	44943.07
Log Likelihood	-25208.34	-24349.40	-22664.13	-22415.60
N	5461	5461	5461	5461
N groups: Reviewer ID	4972	4972	4972	4972
N groups: Journal ID	1324	1324	1324	1324

***p < 0.001; **p < 0.01; *p < 0.05

Table S3.23: Predicting the prevalence of categories related to 'helpfulness'. Sample is limited to reviewers classified as 'female'. Table shows coefficients from mixed effects linear regression models. Standard errors in parentheses.

	**Suggestion and Solution**	**Example**	**Criticism**	**Praise**
(Intercept)	38.74 (1.40)***	12.92 (1.20)***	15.37 (1.00)***	17.39 (1.36)***
JIF group: 2 (ref.: JIF group: 1)	-1.34 (1.94)	1.59 (1.65)	0.78 (1.39)	-0.43 (1.88)
JIF group: 3	-2.13 (1.98)	0.76 (1.75)	1.37 (1.39)	1.80 (1.91)
JIF group: 4	-0.85 (2.01)	3.06 (1.78)	2.95 (1.40)*	-2.80 (1.94)
JIF group: 5	-0.43 (2.00)	1.56 (1.73)	0.62 (1.42)	-2.55 (1.94)
JIF group: 6	-2.54 (2.06)	3.00 (1.78)	0.38 (1.47)	-3.15 (1.99)
JIF group: 7	-2.00 (2.06)	2.25 (1.79)	-1.27 (1.46)	-3.02 (1.99)
JIF group: 8	-4.10 (2.14)	-0.76 (1.87)	0.69 (1.52)	-3.09 (2.07)
JIF group: 9	-6.23 (2.00)**	2.53 (1.80)	2.70 (1.39)	-2.60 (1.93)
JIF group: 10	-4.98 (2.13)*	2.74 (1.91)	1.28 (1.48)	-4.96 (2.05)*
AIC	20891.48	19799.35	19501.45	20804.02
BIC	20966.35	19874.22	19576.31	20878.88
Log Likelihood	-10432.74	-9886.68	-9737.73	-10389.01
N	2342	2342	2342	2342
N groups: Reviewer ID	2213	2213	2213	2213
N groups: Journal ID	858	858	858	858

***p < 0.001; **p < 0.01; *p < 0.05

Table S3.24: Predicting the prevalence of categories related to 'helpfulness'. Sample is limited to reviewers classified as 'male'. Table shows coefficients from mixed effects linear regression models. Standard errors in parentheses.

	**Suggestion and Solution**	**Example**	**Criticism**	**Praise**
(Intercept)	35.75 (0.99)***	10.92 (0.83)***	16.23 (0.72)***	17.02 (0.93)***
JIF group: 2 (ref.: JIF group: 1)	-2.20 (1.41)	1.50 (1.19)	-0.15 (1.02)	-0.25 (1.32)
JIF group: 3	-0.49 (1.47)	3.37 (1.25)**	-0.98 (1.06)	-3.18 (1.37)*
JIF group: 4	-0.51 (1.45)	2.25 (1.25)	1.57 (1.04)	-1.74 (1.34)
JIF group: 5	-1.60 (1.42)	2.47 (1.21)*	-0.36 (1.02)	-1.49 (1.32)
JIF group: 6	-3.27 (1.42)*	1.79 (1.22)	0.56 (1.02)	-1.43 (1.32)
JIF group: 7	-2.09 (1.41)	3.64 (1.21)**	1.36 (1.01)	-3.05 (1.31)*
JIF group: 8	-1.63 (1.44)	1.89 (1.26)	0.30 (1.03)	-3.43 (1.33)**
JIF group: 9	-5.44 (1.50)***	3.42 (1.32)**	2.96 (1.06)**	-3.12 (1.37)*
JIF group: 10	-6.11 (1.45)***	1.99 (1.26)	1.26 (1.03)	-1.70 (1.33)
AIC	48540.33	45582.85	45530.65	48298.89
BIC	48626.20	45668.72	45616.52	48384.76
Log Likelihood	-24257.17	-22778.42	-22752.33	-24136.45
N	5461	5461	5461	5461
N groups: Reviewer ID	4972	4972	4972	4972
N groups: Journal ID	1324	1324	1324	1324

***$p < 0.001$; **$p < 0.01$; *$p < 0.05$

Sensitivity analysis 5: Replacing the JIF Groups (ranging from 1 to 10) with the raw JIF

In the fifth sensitivity analysis, we replaced the categorical variable JIF Group with the raw journal impact factor (ranging from 0.210 to 74.699, with a mean of 3.726 and a median of 3.009). The coefficient *JIF (raw)* reports the predicted percentage point change in the prevalence of a content category for a one-unit increase of the raw journal impact factor. Tables S3.25 and S3.26 show the regression results for negative binomial regression models with the count of sentences as the dependent variable. Tables S3.27 and S3.28 show the regression results for linear mixed effects models with the prevalence as the dependent variable.

Table S3.25: Predicting the prevalence of categories related to 'thoroughness'. Models use the raw journal impact factor. Table shows coefficients from negative binomial regression models. Standard errors in parentheses.

	**Materials and Methods**	**Presentation and Reporting**	**Results and Discussion**	**Importance and Relevance**
(Intercept)	8.13 (0.21)***	5.70 (0.15)***	2.96 (0.09)***	1.47 (0.05)***
JIF (raw)	0.31 (0.04)***	0.07 (0.03)*	0.08 (0.02)***	0.06 (0.01)***
AIC	72334.52	66407.20	55486.54	41052.95
Log Likelihood	-36162.26	-33198.60	-27738.27	-20521.48
N	10000	10000	10000	10000
N groups: Journal ID	1644	1644	1644	1644
N groups: Reviewer ID	9259	9259	9259	9259

$^{***}p < 0.001$; $^{**}p < 0.01$; $^{*}p < 0.05$

Table S3.26: Predicting the prevalence of categories related to 'helpfulness'. Models use the raw journal impact factor. Table shows coefficients from negative binomial regression models. Standard errors in parentheses.

	**Suggestion and Solution**	**Example**	**Criticism**	**Praise**
(Intercept)	6.22 (0.15)***	2.92 (0.13)***	2.93 (0.09)***	1.55 (0.04)***
JIF (raw)	0.09 (0.03)**	0.08 (0.03)**	0.10 (0.02)***	0.05 (0.01)***
AIC	66348.73	62559.92	54815.53	37824.04
Log Likelihood	-33169.37	-31274.96	-27402.77	-18907.02
N	10000	10000	10000	10000
N groups: Journal ID	1644	1644	1644	1644
N groups: Reviewer ID	9259	9259	9259	9259

$^{***}p < 0.001$; $^{**}p < 0.01$; $^{*}p < 0.05$

Table S3.27: Predicting the prevalence of categories related to 'thoroughness'. Models use the raw journal impact factor. Table shows coefficients from mixed effects linear regression models. Standard errors in parentheses.

	**Materials and Methods**	**Presentation and Reporting**	**Results and Discussion**	**Importance and Relevance**
(Intercept)	43.88 (0.52)***	31.97 (0.40)***	16.05 (0.29)***	10.97 (0.27)***
JIF (raw)	0.55 (0.11)***	-0.46 (0.08)***	0.03 (0.06)	-0.06 (0.06)
AIC	92492.73	89629.58	83268.17	82220.75
BIC	92528.78	89665.63	83304.23	82256.80
Log Likelihood	-46241.36	-44809.79	-41629.09	-41105.38
N	10000	10000	10000	10000
N groups: Reviewer ID	9259	9259	9259	9259
N groups: Journal ID	1644	1644	1644	1644

$^{***}p < 0.001$; $^{**}p < 0.01$; $^{*}p < 0.05$

Table S3.28: Predicting the prevalence of categories related to 'helpfulness'. Models use the raw journal impact factor. Table shows coefficients from mixed effects linear regression models. Standard errors in parentheses.

	**Suggestion and Solution**	**Example**	**Criticism**	**Praise**
(Intercept)	36.04 (0.40)***	13.30 (0.34)***	16.26 (0.27)***	15.41 (0.36)***
JIF (raw)	-0.40 (0.08)***	0.05 (0.07)	0.07 (0.06)	-0.17 (0.08)*
AIC	89199.50	83856.36	83362.25	88340.90
BIC	89235.55	83892.41	83398.30	88376.95
Log Likelihood	-44594.75	-41923.18	-41676.12	-44165.45
N	10000	10000	10000	10000
N groups: Reviewer ID	9259	9259	9259	9259
N groups: Journal ID	1644	1644	1644	1644

***p < 0.001; **p < 0.01; *p < 0.05
