# Supplementary material for: Relationship between journal impact factor and the thoroughness and helpfulness of peer reviews
Source: PLoS Biol. 2023 Aug 29;21(8):e3002238. doi: 10.1371/journal.pbio.3002238 (PMC10464996; doi:10.1371/journal.pbio.3002238)

Supporting information

Severin et al.: Relationship between Journal Impact Factor and the Thoroughness and Helpfulness of Peer Reviews

S1 File. Journals and disciplines included in the study

The ten journals from each journal impact factor group that provided the largest number of peer review reports and all 1664 journals included in the analysis listed in alphabetical order. The numbers in parentheses represent the JIF and the number of reviews included in the sample.

Table S1.1: The ten journals from each journal impact factor group that provided most peer review reports.

Journal impact factor group									
1	2	3	4	5	6	7	8	9	10
J of International Medical Research (31)	European J of Ophthalmology (35)	International J of Dermatology (58)	BMJ Open (103)	Physics in Medicine and Biology (64)	Environmental Science and Pollution Research (59)	Magnetic Resonance in Medicine (40)	Plastic and Reconstructive Surgery (65)	Bioinformatics (71)	Nucleic Acids Research (86)
Echocardiography (29)	J of Cardiac Surgery (32)	Frontiers in Psychology (45)	The Laryngoscope (60)	J of Advanced Nursing (36)	Cancer Medicine (48)	J of Magnetic Resonance Imaging (38)	Human Brain Mapping (51)	British J of Surgery (62)	American J of Transplantation (66)
International J of Environmental Analytical Chemistry (24)	J of Cosmetic Dermatology (26)	Human & Experimental Toxicology (43)	Dermatologic Therapy (51)	Transfusion (31)	J of Biomolecular Structure and Dynamics (37)	J of Neural Engineering (29)	Frontiers in Microbiology (35)	Molecular Ecology (60)	Allergy (62)
ANZ J of Surgery (23)	Clinical Transplantation (23)	J of Clinical Nursing (38)	International J of Clinical Practice (41)	International Forum of Allergy & Rhinology (25)	The J of Dermatology (29)	J of Thrombosis and Haemostasis (29)	International J of Cancer (33)	Diabetes, Obesity and Metabolism (57)	eLife (55)
J of Orthopaedic Surgery (23)	J of Clinical Laboratory Analysis (22)	Natural Product Research (34)	Pediatric Blood and Cancer (41)	Head & Neck (23)	Transplant International (28)	Phytotherapy Research (29)	Frontiers in Immunology (30)	Environmental Research Letters (55)	Ecology Letters (42)
J of Obstetrics and Gynaecology Research (19)	The J of Maternal-Fetal & Neonatal Medicine (22)	The British J of Radiology (29)	Pediatric Pulmonology (32)	Oral Diseases (23)	Oikos (26)	BMC Genomics (28)	Cancer Science (29)	J of Cellular Physiology (37)	Hepatology (38)
Pediatrics International (19)	Acta Radiologica (21)	Environmental Technology (26)	Artificial Organs (24)	J of Pharmacy and Pharmacology (22)	J of Gastroenterology and Hepatology (25)	British J of Clinical Pharmacology (27)	Transplantation (28)	Rheumatology (33)	Global Change Biology (35)
Pediatric Dermatology (17)	American J of Perinatology (20)	Technology in Cancer Research & Treatment (21)	J of Cardiovascular Electrophysiology (24)	Frontiers in Neurology (21)	European J of Neuroscience (24)	Applied and Environmental Microbiology (25)	Frontiers in Oncology (26)	J of Bone and Mineral Research (31)	British J of Dermatology (34)
International J of Ophthalmology (16)	Current Eye Research (15)	Andrologia (20)	Physiological Measurement (24)	Colorectal Disease (20)	Reproduction (24)	Diseases of the Colon & Rectum (25)	Antimicrobial Agents and Chemotherapy (24)	Liver International (24)	IEEE Transactions on Medical Imaging (30)
Pacing and Clinical Electrophysiology (16)	Australasian J of Dermatology (14)	Brain and Behavior (20)	Annals of Pharmacotherapy (20)	The J of Clinical Hypertension (18)	Scandinavian J of Medicine & Science in Sports (24)	J of Biogeography (23)	BJU International (24)	J of Antimicrobial Chemotherapy (23)	Alimentary Pharmacology & Therapeutics (27)

The journal (J) and number of review reports (in brackets) are listed. Journal impact factor groups were defined by deciles (1=lowest, 10=highest).

All 1664 journals included in the analysis are listed in alphabetical order below. The numbers in parentheses represent the 2019 Journal Impact Factor and the number of reviews included in the sample.

AAPS PharmSciTech (JIF: 2.401; n=1); Academic Emergency Medicine (JIF: 3.064; n=18); Academic Medicine (JIF: 5.354; n=9); Accountability in Research (JIF: 1.458; n=1); Acta Amazonica (JIF: 0.768; n=1); Acta Anaesthesiologica Scandinavica (JIF: 2.05; n=11); Acta Biochimica et Biophysica Sinica (JIF: 2.836; n=1); Acta Cardiologica (JIF: 1.208; n=1); Acta Cirurgica Brasileira (JIF: 0.974; n=2); Acta Clinica Belgica: International Journal of Clinical and Laboratory Medicine (JIF: 1.111; n=1); Acta Crystallographica Section D Structural Biology (JIF: 5.266; n=1); Acta Haematologica (JIF: 1.196; n=1); Acta Neurologica Scandinavica (JIF: 2.684; n=13); Acta Obstetricia et Gynecologica Scandinavica (JIF: 2.77; n=11); Acta Odontologica Scandinavica (JIF: 1.573; n=4); Acta Oecologica (JIF: 1.22; n=1); Acta Oncologica (JIF: 3.701; n=4); Acta Ophthalmologica (JIF: 3.362; n=12); Acta Oto-Laryngologica (JIF: 1.157; n=7); Acta Paediatrica (JIF: 2.111; n=14); Acta Paulista de Enfermagem (JIF: 0.512; n=5); Acta Physiologica (JIF: 5.542; n=3); Acta Psychiatrica Scandinavica (JIF: 5.362; n=15); Acta Radiologica (JIF: 1.635; n=21); Adapted Physical Activity Quarterly (JIF: 1.462; n=1); Adaptive Behavior (JIF: 0.929; n=2); Addiction (JIF: 6.343; n=16); Addiction Biology (JIF: 4.121; n=9); Advanced Drug Delivery Reviews (JIF: 13.3; n=1); Advances in Medical Sciences (JIF: 2.57; n=1); Advances in Rheumatology (JIF: 0.854; n=1); Advances in Therapy (JIF: 3.871; n=1); Aesthetic Surgery Journal (JIF: 3.799; n=3); African Health Sciences (JIF: 0.69; n=8); African Journal of Ecology (JIF: 0.713; n=10); Age and Ageing (JIF: 4.902; n=1); Aging (JIF: 4.831; n=19); Aging & Mental Health (JIF: 2.478; n=13); Aging and Disease (JIF: 5.402; n=1); Aging Cell (JIF: 7.238; n=8); Aging Male (JIF: 2.5; n=3); Aging, Neuropsychology and Cognition (JIF: 1.75; n=4); Agriculture, Ecosystems and Environment (JIF: 4.241; n=1); AIDS (JIF: 4.511; n=5); Aktuelle Rheumatologie (JIF: 0.316; n=1); Alimentary Pharmacology & Therapeutics (JIF: 7.515; n=27); Allergy (JIF: 8.706; n=62); Alzheimer's & Dementia: The Journal of the Alzheimer's Association (JIF: 17.127; n=5); Ambio (JIF: 4.778; n=3); American Journal of Alzheimer's Disease & Other Dementias (JIF: 1.544; n=1); American Journal of Audiology (JIF: 1.558; n=2); American Journal of Cardiology (JIF: 2.57; n=1); American Journal of Clinical Oncology (JIF: 1.907; n=1); American Journal of Hematology (JIF: 6.973; n=7); American Journal of Hospice and Palliative Medicine (JIF: 1.638; n=9); American Journal of Human Biology (JIF: 1.558; n=1); American Journal of Hypertension (JIF: 2.669; n=10); American Journal of Kidney Diseases (JIF: 6.618; n=1); American Journal of Medical Genetics Part A (JIF: 2.125; n=16); American Journal of Medical Genetics Part B: Neuropsychiatric Genetics (JIF: 3.387; n=3); American Journal of Medical Quality (JIF: 1.426; n=1); American Journal of Neuroradiology (JIF: 3.381; n=9); American Journal of Orthodontics and Dentofacial Orthopedics (JIF: 1.96; n=1); American Journal of Perinatology (JIF: 1.474; n=20); American Journal of Physical Medicine & Rehabilitation (JIF: 1.838; n=5); American Journal of Physiology-Cell Physiology (JIF: 3.485; n=3); American Journal of Physiology-Endocrinology and Metabolism (JIF: 3.469; n=1); American Journal of Physiology-Gastrointestinal and Liver Physiology (JIF: 3.725; n=5); American Journal of Physiology-Heart and Circulatory Physiology (JIF: 3.864; n=2); American Journal of Physiology-Lung Cellular and Molecular Physiology (JIF: 4.406; n=1); American Journal of Physiology-Regulatory, Integrative and Comparative Physiology (JIF: 2.992; n=3); American Journal of Physiology-Renal Physiology (JIF: 3.144; n=3); American Journal of Psychiatry (JIF: 14.119; n=3); American Journal of Reproductive Immunology (JIF: 2.739; n=8); American Journal of Rhinology & Allergy (JIF: 1.943; n=6); American Journal of Transplantation (JIF: 7.338; n=66); Amino Acids (JIF: 3.063; n=1); Amyloid: the International Journal of Experimental and Clinical Investigation: the Official Journal of the International Society of Amyloidosis (JIF: 4.323; n=3); Amyotrophic Lateral Sclerosis and Frontotemporal Degeneration (JIF: 3.286; n=2); Anaesthesia and Intensive Care (JIF: 1.539; n=2); Analytical Biochemistry (JIF: 2.877; n=1); Anatomical Science International (JIF: 1.512; n=1); Andrologia (JIF: 1.951; n=20); Andrology (JIF: 2.86; n=15); Anesthesia & Analgesia (JIF: 4.305; n=9); Anesthesiology (JIF: 7.067; n=10); Angiology (JIF: 2.255; n=6); Annals of Clinical Biochemistry (JIF: 2.044; n=1); Annals of Clinical Microbiology and Antimicrobials (JIF: 2.705; n=2); Annals of General Psychiatry (JIF: 2.157; n=1); Annals of Human Biology (JIF: 1.535; n=1); Annals of Human Genetics (JIF: 1.368; n=3); Annals of Medicine (JIF: 3.243; n=10); Annals of Microbiology (JIF: 1.528; n=1); Annals of Noninvasive Electrocardiology (JIF: 1.131; n=3); Annals of Otology, Rhinology & Laryngology (JIF: 1.284; n=12); Annals of Palliative Medicine (JIF: 1.681; n=4); Annals of Pharmacotherapy (JIF: 2.406; n=20); Annals of Surgery (JIF: 10.13; n=9); Annals of Surgical Oncology (JIF: 4.061; n=3); Annals of Translational Medicine (JIF: 3.297; n=9); Anti-Cancer Agents in Medicinal Chemistry (JIF: 2.049; n=1); Antimicrobial Agents and Chemotherapy (JIF: 4.904; n=24); Antimicrobial Resistance & Infection Control (JIF: 3.594; n=1); Antioxidants (JIF: 5.014; n=3); Anxiety, Stress & Coping (JIF: 2.25; n=1); ANZ Journal of Surgery (JIF: 1.355; n=23); Aphasiology (JIF: 1.72; n=5); Appetite (JIF: 3.608; n=2); Applied and Environmental Microbiology (JIF: 4.016; n=25); Applied Developmental Science (JIF: 2.75; n=1); Applied Neuropsychology: Adult (JIF: 1.488; n=1); Applied Neuropsychology: Child (JIF: 1.293; n=4); Applied Physiology, Nutrition, and Metabolism (JIF: 2.522; n=11); Applied Psychological Measurement (JIF: 1.326; n=2); Applied Psychology: An International Review (JIF: 2.808; n=6); Applied Psychology: Health and Well-Being (JIF: 2.357; n=2); Aquatic Conservation: Marine and Freshwater Ecosystems (JIF: 2.572; n=1); Aquatic Ecosystem Health & Management (JIF: 0.761; n=1); Archiv der Pharmazie (JIF: 2.59; n=5); Archive for the Psychology of Religion (JIF: 0.517; n=1); Archives of Endocrinology and Metabolism (JIF: 1.802; n=3); Archives of Gerontology and Geriatrics (JIF: 2.128; n=1); Archives of Gynecology and Obstetrics (JIF: 2.283; n=1); Archives of Oral Biology (JIF: 1.931; n=1); Archives of Physiology and Biochemistry (JIF: 2.575; n=3); Archives of Suicide Research (JIF: 2.274; n=2); Archives of Virology (JIF: 2.243; n=7); Archivum Immunologiae et Therapiae Experimentalis (JIF: 3.2; n=1); Arctic, Antarctic, and Alpine Research (JIF: 1.784; n=1); Arquivos Brasileiros de Cardiologia (JIF: 1.45; n=7); Arquivos Brasileiros de Oftalmologia (JIF: 0.617; n=7); Arquivos de Neuro-Psiquiatria (JIF: 1.008; n=7); Arteriosclerosis, Thrombosis, and Vascular Biology (JIF: 6.604; n=2); Arthritis & Rheumatology (JIF: 9.586; n=7); Arthritis Care & Research (JIF: 4.056; n=12); Arthritis Research & Therapy (JIF: 4.103; n=1); Artificial Cells, Nanomedicine, and Biotechnology (JIF: 3.343; n=9); Artificial Intelligence in Medicine (JIF: 4.383; n=1); Artificial Organs (JIF: 2.259; n=24); Asia-Pacific Journal of Clinical Oncology (JIF: 2.012; n=4); Asian Journal of Social Psychology (JIF: 1.063; n=2); Asian Pacific Journal of Allergy and Immunology (JIF: 1.247; n=1); ASN Neuro (JIF: 4.167; n=1); Assessment (JIF: 3.706; n=10); Assistive Technology (JIF: 1.197; n=3); Atherosclerosis (JIF: 3.919; n=2); Atmospheric Pollution Research (JIF: 3.527; n=1); Austral Ecology (JIF: 1.551; n=5); Australasian Journal of Dermatology (JIF: 1.789; n=14); Australasian Psychiatry (JIF: 1.029; n=6); Australian and New Zealand Journal of Obstetrics and Gynaecology (JIF: 1.644; n=5); Australian Dental Journal (JIF: 1.401; n=6); Australian Endodontic Journal (JIF: 1.12; n=3); Australian Health Review (JIF: 1.32; n=13); Australian Journal of General Practice (JIF: 0.723; n=4); Australian Journal of Psychology (JIF: 1.486; n=2); Australian Psychologist (JIF: 1.458; n=3); Autism (JIF: 4.609; n=8); Autism Research (JIF: 3.727; n=1); Autoimmunity (JIF: 2.125; n=2); Autophagy (JIF: 9.77; n=2); Basic and Applied Social Psychology (JIF: 1.577; n=4); Behavioral Interventions (JIF: 0.731; n=4); Behavioral Medicine (JIF: 1.721; n=1); Behavioral Sleep Medicine (JIF: 2.39; n=5); Behaviour & Information Technology (JIF: 1.781; n=7); Behaviour Research and Therapy (JIF: 4.5; n=1); Bioanalysis (JIF: 2.371; n=4); Biocatalysis and Biotransformation (JIF: 1.863; n=2); Biochemical Journal (JIF: 4.097; n=2); Biochemical Pharmacology (JIF: 4.96; n=2); Biochemistry (JIF: 2.865; n=1); Biochemistry and Cell Biology (JIF: 2.46; n=3); Biochemistry and Molecular Biology Education (JIF: 0.924; n=1); Biochimie (JIF: 3.413; n=1); Bioelectrochemistry (JIF: 4.722; n=1); Bioelectromagnetics (JIF: 2.278; n=8); Bioengineered (JIF: 2.205; n=11); BioFactors (JIF: 4.734; n=4); Biofuels, Bioproducts and Biorefining (JIF: 4.528; n=3); Biogeosciences (JIF: 3.48; n=1); Bioinformatics (JIF: 5.61; n=71); Biological and Pharmaceutical Bulletin (JIF: 1.863; n=1); Biological Conservation (JIF: 4.711; n=2); Biological Psychiatry (JIF: 12.095; n=1); Biological Psychology (JIF: 2.763; n=2); Biological Research for Nursing (JIF: 1.789;

n=1); Biological Reviews (JIF: 10.701; n=1); Biological Rhythm Research (JIF: 0.826; n=1); Biology Direct (JIF: 2.193; n=3); Biomacromolecules (JIF: 6.092; n=1); Biomarkers (JIF: 2.07; n=5); Biomarkers in Medicine (JIF: 2.479; n=9); Biomass and Bioenergy (JIF: 3.551; n=2); BioMed Research International (JIF: 2.276; n=3); BioMedical Engineering Online (JIF: 2.059; n=1); Biomedical Microdevices (JIF: 2.176; n=1); Biomedical Optics Express (JIF: 3.921; n=3); Biomedicine & Pharmacotherapy (JIF: 4.545; n=2); Biopharmaceutics & Drug Disposition (JIF: 1.663; n=3); Biophysical Journal (JIF: 3.854; n=2); Biopolymers (JIF: 1.854; n=3); Biopreservation and Biobanking (JIF: 1.906; n=2); Bioremediation Journal (JIF: 1.724; n=3); Bioscience Reports (JIF: 2.942; n=10); Bioscience, Biotechnology, and Biochemistry (JIF: 1.516; n=6); BioSystems (JIF: 1.808; n=1); Biota Neotropica (JIF: 0.922; n=1); Biotechnic and Histochemistry (JIF: 1.203; n=1); BioTechniques (JIF: 1.541; n=5); Biotechnology Advances (JIF: 10.744; n=2); Biotechnology and Applied Biochemistry (JIF: 1.638; n=7); Biotechnology and Biotechnological Equipment (JIF: 1.186; n=1); Biotechnology Letters (JIF: 1.977; n=2); Biotechnology Progress (JIF: 2.334; n=8); Biotropica (JIF: 2.09; n=10); Bipolar Disorders (JIF: 5.41; n=7); Birth (JIF: 2.705; n=3); Birth Defects Research (JIF: 1.896; n=6); BJOG: An International Journal of Obstetrics & Gynaecology (JIF: 4.663; n=5); BJU International (JIF: 4.806; n=24); Blood (JIF: 17.543; n=3); BMC Anesthesiology (JIF: 1.695; n=2); BMC Biology (JIF: 6.765; n=1); BMC Biotechnology (JIF: 2.312; n=4); BMC Cancer (JIF: 3.15; n=6); BMC Cardiovascular Disorders (JIF: 2.078; n=4); BMC Complementary and Alternative Medicine (JIF: 2.833; n=3); BMC Developmental Biology (JIF: 2; n=1); BMC Ecology (JIF: 2.029; n=1); BMC Endocrine Disorders (JIF: 1.994; n=4); BMC Gastroenterology (JIF: 2.489; n=3); BMC Genomics (JIF: 3.594; n=28); BMC Geriatrics (JIF: 3.077; n=5); BMC Health Services Research (JIF: 1.987; n=14); BMC Immunology (JIF: 2.495; n=1); BMC Infectious Diseases (JIF: 2.688; n=5); BMC Medical Genomics (JIF: 2.57; n=4); BMC Medical Imaging (JIF: 1.792; n=3); BMC Medical Informatics and Decision Making (JIF: 2.317; n=1); BMC Microbiology (JIF: 2.989; n=3); BMC Musculoskeletal Disorders (JIF: 1.879; n=14); BMC Neurology (JIF: 2.356; n=2); BMC Ophthalmology (JIF: 1.413; n=2); BMC Oral Health (JIF: 1.911; n=1); BMC Pediatrics (JIF: 1.909; n=2); BMC Pregnancy and Childbirth (JIF: 2.239; n=6); BMC Psychiatry (JIF: 2.704; n=7); BMC Pulmonary Medicine (JIF: 2.813; n=1); BMC Surgery (JIF: 1.912; n=2); BMC Systems Biology (JIF: 2.048; n=1); BMC Urology (JIF: 1.592; n=2); BMC Women's Health (JIF: 1.544; n=3); BMJ (JIF: 30.223; n=1); BMJ Open (JIF: 2.496; n=103); BMJ Supportive & Palliative Care (JIF: 2.681; n=1); Bone (JIF: 4.147; n=2); Bone and Joint Research (JIF: 3.532; n=4); Bone Marrow Transplantation (JIF: 4.725; n=1); Brain and Behavior (JIF: 2.091; n=20); Brain Connectivity (JIF: 5.263; n=1); Brain Injury (JIF: 1.69; n=10); Brain Pathology (JIF: 5.568; n=4); Brain Research (JIF: 2.733; n=1); Brain Sciences (JIF: 3.332; n=1); Brain Topography (JIF: 2.759; n=1); Brazilian Archives of Biology and Technology (JIF: 0.579; n=7); Brazilian Journal of Biology (JIF: 1.266; n=1); Brazilian Journal of Cardiovascular Surgery (JIF: 1.053; n=8); Brazilian Journal of Medical and Biological Research (JIF: 2.023; n=1); British Dental Journal (JIF: 1.306; n=1); British Journal of Cancer (JIF: 5.791; n=9); British Journal of Clinical Pharmacology (JIF: 3.74; n=27); British Journal of Dermatology (JIF: 7; n=34); British Journal of Educational Psychology (JIF: 2.506; n=1); British Journal of General Practice (JIF: 4.19; n=4); British Journal of Haematology (JIF: 5.518; n=15); British Journal of Hospital Medicine (JIF: 0.429; n=1); British Journal of Neurosurgery (JIF: 1.29; n=6); British Journal of Ophthalmology (JIF: 3.611; n=12); British Journal of Pharmacology (JIF: 7.73; n=27); British Journal of Surgery (JIF: 5.676; n=62); British Medical Bulletin (JIF: 2.835; n=1); Burns & Trauma (JIF: 3.088; n=2); Canadian Association of Radiologists Journal (JIF: 1.726; n=3); Canadian Journal of Microbiology (JIF: 1.793; n=1); Canadian Journal of Neurological Sciences (JIF: 1.714; n=7); Canadian Journal of Physiology and Pharmacology (JIF: 1.946; n=3); Canadian Journal of Psychiatry (JIF: 3.313; n=3); Canadian Water Resources Journal (JIF: 1.902; n=1); Cancer (JIF: 5.742; n=15); Cancer Biology & Therapy (JIF: 3.659; n=4); Cancer Biology and Medicine (JIF: 5.432; n=3); Cancer Biomarkers (JIF: 3.436; n=2); Cancer Chemotherapy and Pharmacology (JIF: 2.967; n=2); Cancer Control (JIF: 3.103; n=8); Cancer Cytopathology (JIF: 3.703; n=1); Cancer Discovery (JIF: 29.497; n=1); Cancer Immunology, Immunotherapy (JIF: 5.442; n=1); Cancer Investigation (JIF: 2.398; n=5); Cancer Medicine (JIF: 3.491; n=48); Cancer Research (JIF: 9.727; n=5); Cancer Science (JIF: 4.966; n=29); Cancers (JIF: 6.126; n=2); Carbon Management (JIF: 1.667; n=1); Cardiovascular Diagnosis and Therapy (JIF: 2.615; n=2); Cardiovascular Research (JIF: 8.168; n=12); Cardiovascular Therapeutics (JIF: 2.538; n=3); Cartilage (JIF: 3.857; n=4); Catheterization and Cardiovascular Interventions (JIF: 2.044; n=17); Cell & Bioscience (JIF: 5.026; n=1); Cell Biochemistry and Function (JIF: 2.632; n=1); Cell Biology and Toxicology (JIF: 6.284; n=1); Cell Biology International (JIF: 2.571; n=8); Cell Cycle (JIF: 3.699; n=5); Cell Death and Disease (JIF: 6.304; n=11); Cell Death Discovery (JIF: 4.114; n=1); Cell Discovery (JIF: 6.255; n=1); Cell Proliferation (JIF: 5.753; n=7); Cell Reports (JIF: 8.109; n=1); Cell Systems (JIF: 8.673; n=1); Cell Transplantation (JIF: 3.341; n=6); Cells (JIF: 4.366; n=4); Cellular Microbiology (JIF: 3.43; n=2); Cephalalgia (JIF: 4.868; n=11); Cerebral Cortex (JIF: 5.043; n=1); Channels (JIF: 2.311; n=1); Chembiochem (JIF: 2.576; n=3); Chemical Biology & Drug Design (JIF: 2.548; n=13); Chemical Research in Toxicology (JIF: 3.184; n=2); Chemical Senses (JIF: 2.261; n=1); Chemico-Biological Interactions (JIF: 3.723; n=1); Chemistry and Biodiversity (JIF: 2.039; n=6); Chemistry and Ecology (JIF: 1.4; n=3); ChemMedChem (JIF: 3.124; n=1); Chemosphere (JIF: 5.778; n=1); Chest (JIF: 8.308; n=21); Child Neuropsychology (JIF: 2.405; n=1); Child: Care, Health and Development (JIF: 1.828; n=4); Childhood Obesity (JIF: 2.548; n=1); Chinese Journal of Cancer Research (JIF: 4.135; n=1); Chinese Medical Journal (JIF: 1.585; n=4); Chronobiology International (JIF: 2.486; n=9); Circulation (JIF: 23.603; n=4); Circulation: Cardiovascular Imaging (JIF: 5.691; n=3); Circulation: Cardiovascular Interventions (JIF: 5.493; n=1); Circulation: Cardiovascular Quality and Outcomes (JIF: 5.071; n=1); Circulation: Genomic and Precision Medicine (JIF: 4.063; n=1); Circulation: Heart Failure (JIF: 6.033; n=3); Clean - Soil, Air, Water (JIF: 1.603; n=1); Clean Technologies and Environmental Policy (JIF: 2.429; n=1); Climacteric (JIF: 2.566; n=3); Clinica Y Salud (JIF: 0.452; n=1); Clinical & Experimental Allergy (JIF: 4.217; n=16); Clinical & Experimental Immunology (JIF: 3.532; n=6); Clinical & Experimental Ophthalmology (JIF: 2.832; n=3); Clinical & Translational Immunology (JIF: 6.464; n=4); Clinical Anatomy (JIF: 1.967; n=9); Clinical and Applied Thrombosis/Hemostasis (JIF: 1.374; n=6); Clinical and Experimental Dermatology (JIF: 1.977; n=16); Clinical and Experimental Hypertension (JIF: 1.789; n=1); Clinical and Experimental Optometry (JIF: 1.918; n=5); Clinical and Experimental Pharmacology and Physiology (JIF: 2.456; n=12); Clinical and Translational Allergy (JIF: 5.129; n=1); Clinical and Translational Oncology (JIF: 2.737; n=1); Clinical and Translational Science (JIF: 3.373; n=1); Clinical and Vaccine Immunology (JIF: 3.233; n=3); Clinical Biochemistry (JIF: 2.573; n=1); Clinical Biomechanics (JIF: 1.624; n=1); Clinical Cancer Research (JIF: 10.107; n=1); Clinical Cardiology (JIF: 2.248; n=4); Clinical Child Psychology and Psychiatry (JIF: 1.647; n=1); Clinical EEG and Neuroscience (JIF: 1.765; n=2); Clinical Endocrinology (JIF: 3.38; n=18); Clinical Gastroenterology and Hepatology (JIF: 8.549; n=1); Clinical Genetics (JIF: 3.578; n=22); Clinical Implant Dentistry and Related Research (JIF: 3.396; n=6); Clinical Infectious Diseases (JIF: 8.313; n=2); Clinical Interventions in Aging (JIF: 3.023; n=1); Clinical Journal of Sport Medicine (JIF: 3.165; n=1); Clinical Journal of the American Society of Nephrology (JIF: 6.628; n=8); Clinical Kidney Journal (JIF: 3.388; n=5); Clinical Medicine (JIF: 1.883; n=2); Clinical Microbiology and Infection (JIF: 7.117; n=1); Clinical Microbiology Reviews (JIF: 22.556; n=4); Clinical Nursing Research (JIF: 1.538; n=7); Clinical Nutrition (JIF: 6.36; n=1); Clinical Oral Implants Research (JIF: 3.723; n=14); Clinical Otolaryngology (JIF: 2.197; n=14); Clinical Pediatrics (JIF: 1.026; n=10); Clinical Pharmacology & Therapeutics (JIF: 6.565; n=2); Clinical Physiology and Functional Imaging (JIF: 1.704; n=1); Clinical Psychologist (JIF: 1.333; n=1); Clinical Psychology & Psychotherapy (JIF: 2.477; n=5); Clinical Rehabilitation (JIF: 2.599; n=2); Clinical Rheumatology (JIF: 2.394; n=1); Clinical Science (JIF: 5.223; n=2); Clinical Toxicology (JIF: 3.659; n=12); Clinical Transplantation (JIF: 1.665; n=23); Clinical Trials (JIF: 2.462; n=2); Clinics (JIF: 1.435; n=4); CNS and Neurological Disorders - Drug Targets (JIF: 2.761; n=1); CNS Drugs (JIF: 4.786; n=1); CNS Neuroscience & Therapeutics (JIF: 4.074; n=12); Cochrane Database of Systematic Reviews (JIF: 7.89; n=1); Cognition & Emotion (JIF: 2.473; n=10); Cognition, Technology and Work (JIF: 1.206; n=1); Cognitive Behaviour Therapy (JIF: 4.413; n=3); Cognitive Neuroscience (JIF: 3; n=2); Colorectal Disease (JIF: 2.769; n=20); Community Dentistry and Oral Epidemiology (JIF: 2.135; n=6); Comparative Biochemistry and Physiology Part D: Genomics and Proteomics (JIF: 3.011; n=1); Complementary Medicine Research (JIF: 1.089; n=1); Computers in Human Behavior (JIF: 5.003; n=1); Congenital Anomalies (JIF: 1.761; n=7); Congenital Heart Disease (JIF: 1.663; n=3); Conservation Biology (JIF: 5.405; n=1); Conservation Letters (JIF: 6.766; n=1);

Contact Dermatitis (JIF: 3.952; n=7); COPD: Journal of Chronic Obstructive Pulmonary Disease (JIF: 2.5; n=4); Cornea (JIF: 2.215; n=7); Cortex (JIF: 4.009; n=1); Cranio: The Journal of Craniomandibular & Sleep Practice (JIF: 1.173; n=1); Creativity Research Journal (JIF: 1.289; n=1); Criminal Behaviour and Mental Health (JIF: 1.221; n=1); Criminal Justice and Behavior (JIF: 1.523; n=5); Critical Care Medicine (JIF: 7.414; n=18); Critical Reviews in Biochemistry and Molecular Biology (JIF: 7.634; n=1); Critical Reviews in Biotechnology (JIF: 8.108; n=1); Critical Reviews in Environmental Science and Technology (JIF: 8.302; n=2); Critical Reviews in Microbiology (JIF: 7.349; n=2); Critical Reviews in Toxicology (JIF: 4.257; n=1); Culture and Psychology (JIF: 1.71; n=1); Current Alzheimer Research (JIF: 3.047; n=1); Current Biology (JIF: 9.601; n=1); Current Directions in Psychological Science (JIF: 5.117; n=1); Current Drug Delivery (JIF: 1.582; n=2); Current Eye Research (JIF: 1.754; n=15); Current Gene Therapy (JIF: 2.431; n=1); Current Issues in Molecular Biology (JIF: 2.695; n=1); Current Medical Research and Opinion (JIF: 2.271; n=9); Current Medicinal Chemistry (JIF: 4.184; n=1); Current Pharmaceutical Analysis (JIF: 0.923; n=1); Current Pharmaceutical Design (JIF: 2.208; n=1); Cutaneous and Ocular Toxicology (JIF: 1.385; n=5); Cytokine (JIF: 2.952; n=1); Cytometry Part A (JIF: 3.124; n=5); Cytometry Part B: Clinical Cytometry (JIF: 2.07; n=3); Cytopathology (JIF: 1.493; n=5); Database (JIF: 2.593; n=1); Death Studies (JIF: 1.361; n=1); Dental Traumatology (JIF: 1.53; n=6); Dentomaxillofacial Radiology (JIF: 1.796; n=9); Depression and Anxiety (JIF: 4.702; n=1); Dermatitis (JIF: 3.988; n=2); Dermatologic Surgery (JIF: 2.567; n=9); Dermatologic Therapy (JIF: 2.327; n=51); Development and Psychopathology (JIF: 3.385; n=1); Development, Growth & Differentiation (JIF: 1.723; n=1); Developmental Biology (JIF: 2.895; n=1); Developmental Cell (JIF: 10.092; n=1); Developmental Cognitive Neuroscience (JIF: 4.966; n=1); Developmental Dynamics (JIF: 3.275; n=7); Developmental Medicine & Child Neurology (JIF: 4.406; n=6); Developmental Psychobiology (JIF: 2.021; n=1); Developmental Science (JIF: 3.722; n=6); Diabetes and Vascular Disease Research (JIF: 2.707; n=1); Diabetes Educator (JIF: 2.087; n=1); Diabetes, Metabolic Syndrome and Obesity: Targets and Therapy (JIF: 2.842; n=1); Diabetes, Obesity and Metabolism (JIF: 5.9; n=57); Diabetes/Metabolism Research and Reviews (JIF: 3.314; n=9); Diabetic Medicine (JIF: 3.083; n=16); Diagnostic Cytopathology (JIF: 1.229; n=3); Diagnostics (JIF: 3.11; n=1); Digestive Endoscopy (JIF: 4.774; n=14); Diseases of the Colon & Rectum (JIF: 3.991; n=25); Diseases of the Esophagus (JIF: 2.386; n=1); Diversity and Distributions (JIF: 3.993; n=21); DNA and Cell Biology (JIF: 3.191; n=1); Dose-Response (JIF: 2.438; n=1); Drug and Chemical Toxicology (JIF: 2.405; n=5); Drug Delivery (JIF: 4.902; n=8); Drug Delivery and Translational Research (JIF: 2.664; n=1); Drug Development and Industrial Pharmacy (JIF: 2.365; n=15); Drug Development Research (JIF: 1.902; n=5); Drug Metabolism and Disposition (JIF: 3.231; n=1); Drug Testing and Analysis (JIF: 2.903; n=6); Drugs of Today (JIF: 1.373; n=2); Ear and Hearing (JIF: 3.129; n=5); Ear, Nose and Throat Journal (JIF: 0.859; n=7); Early Intervention in Psychiatry (JIF: 2.257; n=3); Earth's Future (JIF: 6.141; n=1); EBioMedicine (JIF: 5.736; n=1); Echocardiography (JIF: 1.393; n=29); Ecography (JIF: 6.455; n=11); Ecohydrology (JIF: 2.767; n=8); Ecological Applications (JIF: 4.248; n=6); Ecological Engineering (JIF: 3.512; n=1); Ecological Indicators (JIF: 4.229; n=1); Ecological Management & Restoration (JIF: 1.086; n=1); Ecological Modelling (JIF: 2.497; n=1); Ecological Monographs (JIF: 7.722; n=4); Ecological Research (JIF: 1.58; n=3); Ecology (JIF: 4.7; n=19); Ecology and Evolution (JIF: 2.392; n=7); Ecology Letters (JIF: 8.665; n=42); Ecoscience (JIF: 1.25; n=3); Ecosphere (JIF: 2.878; n=11); Ecosystem Services (JIF: 6.33; n=2); Ecotoxicology and Environmental Safety (JIF: 4.872; n=2); Educational and Psychological Measurement (JIF: 1.941; n=3); Educational Psychology (JIF: 1.586; n=2); eLife (JIF: 7.08; n=55); EMBO Molecular Medicine (JIF: 8.821; n=1); EMBO Reports (JIF: 7.497; n=4); Emergency Medicine Australasia (JIF: 1.609; n=7); Emerging Microbes & Infections (JIF: 5.776; n=12); Emotion Review (JIF: 4.258; n=1); Endocrine Connections (JIF: 2.592; n=10); Endocrine Journal (JIF: 1.952; n=1); Endocrine-Related Cancer (JIF: 4.8; n=12); Endocrine, Metabolic & Immune Disorders-Drug Targets (JIF: 1.973; n=2); Endoscopy (JIF: 7.341; n=5); Engenharia Sanitaria E Ambiental (JIF: 0.325; n=1); Engineering in Life Sciences (JIF: 1.934; n=1); Environment International (JIF: 7.577; n=1); Environment, Development and Sustainability (JIF: 2.191; n=2); Environmental Chemistry (JIF: 1.91; n=4); Environmental Development (JIF: 2.4; n=1); Environmental Earth Sciences (JIF: 2.18; n=2); Environmental Geochemistry and Health (JIF: 3.472; n=1); Environmental Microbiology (JIF: 4.933; n=1); Environmental Microbiology Reports (JIF: 2.975; n=1); Environmental Research (JIF: 5.715; n=3); Environmental Research Letters (JIF: 6.096; n=55); Environmental Reviews (JIF: 4.176; n=2); Environmental Science & Technology (JIF: 7.864; n=1); Environmental Science and Pollution Research (JIF: 3.056; n=59); Environmental Science: Processes & Impacts (JIF: 3.238; n=1); Environmental Technology (JIF: 2.213; n=26); Environmental Toxicology and Chemistry (JIF: 3.152; n=11); Environmetrics (JIF: 1.039; n=1); Epidemiology and Psychiatric Sciences (JIF: 5.876; n=1); Epigenetics (JIF: 4.251; n=6); Epigenomics (JIF: 4.112; n=8); ESC Heart Failure (JIF: 3.902; n=18); Ethnicity & Health (JIF: 2.554; n=1); Eukaryotic Cell (JIF: 4.188; n=1); Europace (JIF: 4.045; n=7); European Archives of Oto-Rhino-Laryngology (JIF: 1.809; n=1); European Eating Disorders Review (JIF: 3.56; n=5); European Heart Journal - Cardiovascular Imaging (JIF: 4.841; n=2); European Heart Journal - Cardiovascular Pharmacotherapy (JIF: 6.696; n=6); European Heart Journal (JIF: 22.673; n=12); European Heart Journal: Acute Cardiovascular Care (JIF: 3.813; n=1); European Journal of Applied Physiology (JIF: 2.58; n=1); European Journal of Cancer Care (JIF: 2.161; n=11); European Journal of Cardio-thoracic Surgery (JIF: 3.486; n=9); European Journal of Cardiovascular Nursing (JIF: 2.296; n=3); European Journal of Clinical Investigation (JIF: 3.481; n=4); European Journal of Clinical Nutrition (JIF: 3.291; n=2); European Journal of Dental Education (JIF: 1.05; n=4); European Journal of Developmental Psychology (JIF: 2.063; n=4); European Journal of Emergency Medicine (JIF: 2.17; n=1); European Journal of Endocrinology (JIF: 5.308; n=18); European Journal of Haematology (JIF: 2.22; n=6); European Journal of Heart Failure (JIF: 11.627; n=6); European Journal of Human Genetics (JIF: 3.657; n=1); European Journal of Immunology (JIF: 4.404; n=11); European Journal of Inflammation (JIF: 0.267; n=7); European Journal of Neurology (JIF: 4.516; n=15); European Journal of Neuroscience (JIF: 3.115; n=24); European Journal of Ophthalmology (JIF: 1.642; n=35); European Journal of Oral Sciences (JIF: 2.22; n=3); European Journal of Pediatric Surgery (JIF: 1.703; n=6); European Journal of Pharmaceutical Sciences (JIF: 3.616; n=1); European Journal of Pharmacology (JIF: 3.263; n=15); European Journal of Preventive Cardiology (JIF: 5.864; n=12); European Journal of Psychotraumatology (JIF: 3.478; n=4); European Journal of Social Psychology (JIF: 2.415; n=10); European Journal of Sport Science (JIF: 2.781; n=11); European Journal of Surgical Oncology (EJSO) (JIF: 3.959; n=3); European Journal of Work and Organizational Psychology (JIF: 2.882; n=5); European Respiratory Journal (JIF: 12.339; n=14); European Respiratory Review (JIF: 6.22; n=3); Experimental and Clinical Endocrinology & Diabetes (JIF: 2.058; n=2); Experimental and Molecular Pathology (JIF: 2.28; n=1); Experimental Biology and Medicine (JIF: 3.139; n=2); Experimental Dermatology (JIF: 3.368; n=15); Experimental Lung Research (JIF: 1.702; n=1); Experimental Physiology (JIF: 2.431; n=8); Expert Opinion on Biological Therapy (JIF: 3.224; n=7); Expert Opinion on Drug Delivery (JIF: 4.838; n=3); Expert Opinion on Drug Discovery (JIF: 4.887; n=5); Expert Opinion on Drug Metabolism & Toxicology (JIF: 3.47; n=4); Expert Opinion on Drug Safety (JIF: 3.383; n=10); Expert Opinion on Emerging Drugs (JIF: 2.807; n=2); Expert Opinion on Investigational Drugs (JIF: 5.081; n=4); Expert Opinion on Pharmacotherapy (JIF: 2.878; n=12); Expert Opinion on Therapeutic Patents (JIF: 5.611; n=2); Expert Opinion on Therapeutic Targets (JIF: 5.473; n=2); Expert Review of Anti-Infective Therapy (JIF: 3.767; n=12); Expert Review of Anticancer Therapy (JIF: 3.573; n=5); Expert Review of Clinical Immunology (JIF: 3.792; n=11); Expert Review of Clinical Pharmacology (JIF: 3.481; n=2); Expert Review of Gastroenterology & Hepatology (JIF: 3.514; n=3); Expert Review of Hematology (JIF: 2.573; n=3); Expert Review of Medical Devices (JIF: 2.2; n=3); Expert Review of Molecular Diagnostics (JIF: 4.096; n=6); Expert Review of Neurotherapeutics (JIF: 3.743; n=7); Expert Review of Pharmacoeconomics & Outcomes Research (JIF: 2.032; n=4); Expert Review of Proteomics (JIF: 3.614; n=3); Expert Review of Respiratory Medicine (JIF: 2.725; n=4); Expert Review of Vaccines (JIF: 4.362; n=5); Eye (JIF: 2.455; n=2); Facial Plastic Surgery (JIF: 1.108; n=2); Family Process (JIF: 2.175; n=1); FEBS Letters (JIF: 3.057; n=9); FEBS Open Bio (JIF: 2.231; n=6); Female Pelvic Medicine & Reconstructive Surgery (JIF: 1.503; n=4); Feminism & Psychology (JIF: 1.882; n=2); FEMS Microbiology Ecology (JIF: 3.675; n=9); FEMS Microbiology Letters (JIF: 1.987; n=4); FEMS Microbiology Reviews (JIF: 13.92; n=1); Foot & Ankle International (JIF: 2.292; n=7); Fortschritte der Neurologie Psychiatrie (JIF: 0.642; n=2); Free Radical Research (JIF: 2.839; n=1); Frontiers in Aging Neuroscience (JIF: 4.362; n=5); Frontiers in Behavioral

Neuroscience (JIF: 2.512; n=2); Frontiers in Cell and Developmental Biology (JIF: 5.201; n=9); Frontiers in Cellular and Infection Microbiology (JIF: 4.123; n=7); Frontiers in Cellular Neuroscience (JIF: 3.921; n=2); Frontiers in Ecology and Evolution (JIF: 2.416; n=9); Frontiers in Endocrinology (JIF: 3.644; n=12); Frontiers in Genetics (JIF: 3.258; n=21); Frontiers in Human Neuroscience (JIF: 2.673; n=8); Frontiers in Immunology (JIF: 5.085; n=30); Frontiers in Integrative Neuroscience (JIF: 2.152; n=2); Frontiers in Medicine (JIF: 3.9; n=16); Frontiers in Microbiology (JIF: 4.235; n=35); Frontiers in Molecular Biosciences (JIF: 4.188; n=8); Frontiers in Molecular Neuroscience (JIF: 4.057; n=5); Frontiers in Neural Circuits (JIF: 3.156; n=1); Frontiers in Neuroanatomy (JIF: 3.292; n=2); Frontiers in Neuroinformatics (JIF: 2.649; n=1); Frontiers in Neurology (JIF: 2.889; n=21); Frontiers in Neuroscience (JIF: 3.707; n=17); Frontiers in Oncology (JIF: 4.848; n=26); Frontiers in Pediatrics (JIF: 2.634; n=5); Frontiers in Pharmacology (JIF: 4.225; n=22); Frontiers in Physiology (JIF: 3.367; n=15); Frontiers in Psychiatry (JIF: 2.849; n=15); Frontiers in Psychology (JIF: 2.067; n=45); Frontiers in Systems Neuroscience (JIF: 3.293; n=1); Frontiers of Environmental Science & Engineering (JIF: 4.053; n=3); Frontiers of Medicine (JIF: 3.421; n=1); Functional Ecology (JIF: 4.434; n=10); Future Microbiology (JIF: 2.907; n=5); Future Oncology (JIF: 2.66; n=10); Future Virology (JIF: 0.952; n=4); G3: Genes|Genomes|Genetics (JIF: 2.781; n=1); Gastroenterology Nursing (JIF: 0.557; n=1); Geburtshilfe und Frauenheilkunde (JIF: 2.382; n=2); Gene (JIF: 2.984; n=3); Genes (JIF: 3.759; n=1); Genes to Cells (JIF: 1.655; n=3); Genes, Brain and Behavior (JIF: 3.397; n=3); Genes, Chromosomes and Cancer (JIF: 3.444; n=3); Genesis: The Journal of Genetics and Development (JIF: 1.76; n=1); Genetic Epidemiology (JIF: 1.954; n=4); Genetica (JIF: 1.186; n=1); Genetics (JIF: 4.015; n=3); Genetics and Molecular Biology (JIF: 1.876; n=7); Genome (JIF: 2.037; n=6); Genome Biology (JIF: 10.806; n=1); Genome Research (JIF: 11.093; n=1); Genomics (JIF: 6.205; n=1); Geobiology (JIF: 4.385; n=3); Geomicrobiology Journal (JIF: 1.972; n=3); Geriatric Orthopaedic Surgery & Rehabilitation (JIF: 1.345; n=2); Geriatrics & Gerontology International (JIF: 2.022; n=20); Gerodontology (JIF: 1.339; n=1); Gland Surgery (JIF: 2.19; n=4); Glia (JIF: 5.984; n=6); Global Change Biology (JIF: 8.555; n=35); Global Ecology and Biogeography (JIF: 6.446; n=13); Glycobiology (JIF: 4.06; n=1); Graefe's Archive for Clinical and Experimental Ophthalmology (JIF: 2.396; n=5); Greenhouse Gases: Science and Technology (JIF: 1.979; n=5); Ground Water (JIF: 2.205; n=1); Group Processes and Intergroup Relations (JIF: 2.54; n=7); Gut Microbes (JIF: 7.74; n=6); Gynecologic Oncology (JIF: 4.623; n=1); Gynecological Endocrinology (JIF: 1.571; n=12); Haemophilia (JIF: 2.99; n=10); Hamostaseologie (JIF: 0.873; n=2); Handchirurgie · Mikrochirurgie · Plastische Chirurgie (JIF: 0.84; n=1); Head & Face Medicine (JIF: 1.882; n=1); Head & Neck (JIF: 2.538; n=23); Headache (JIF: 4.041; n=15); Health Informatics Journal (JIF: 2.932; n=7); Health Physics (JIF: 0.853; n=1); Health Psychology Review (JIF: 3.705; n=1); Heart (JIF: 5.213; n=2); Heart Surgery Forum (JIF: 0.404; n=4); Helicobacter (JIF: 4; n=3); Hematological Oncology (JIF: 2.832; n=4); Hematology (JIF: 1.65; n=1); Hepatitis Monthly (JIF: 0.705; n=6); Hepatobiliary & Pancreatic Diseases International (JIF: 2.428; n=1); Hepatobiliary Surgery and Nutrition (JIF: 5.296; n=1); Hepatology (JIF: 14.679; n=38); Heredity (JIF: 3.436; n=1); HIP International (JIF: 1.349; n=2); Hippocampus (JIF: 3.404; n=1); Histopathology (JIF: 3.626; n=9); Historical Biology (JIF: 2.023; n=4); HIV Medicine (JIF: 3.556; n=7); HLA (JIF: 2.955; n=3); Hong Kong Journal of Emergency Medicine (JIF: 0.214; n=2); Hormone and Metabolic Research (JIF: 2.562; n=8); Human & Experimental Toxicology (JIF: 2.067; n=43); Human and Ecological Risk Assessment (HERA) (JIF: 2.3; n=2); Human Brain Mapping (JIF: 4.421; n=51); Human Dimensions of Wildlife (JIF: 1.723; n=2); Human Factors (JIF: 3.165; n=6); Human Fertility (JIF: 2.161; n=1); Human Gene Therapy (JIF: 4.273; n=1); Human Mutation (JIF: 4.124; n=11); Human Pathology (JIF: 2.735; n=1); Human Performance (JIF: 1.172; n=1); Human Psychopharmacology: Clinical and Experimental (JIF: 2.112; n=2); Human Reproduction (JIF: 5.733; n=1); Human Vaccines & Immunotherapeutics (JIF: 2.619; n=6); Hydrological Processes (JIF: 3.256; n=21); Hydrological Sciences Journal (JIF: 2.186; n=4); Hypertension (JIF: 7.713; n=4); Hypertension in Pregnancy (JIF: 1.787; n=1); I-Perception (JIF: 1.535; n=1); IEEE Transactions on Medical Imaging (JIF: 6.685; n=30); IEEE Transactions on NanoBioscience (JIF: 2.791; n=5); IET Nanobiotechnology (JIF: 1.859; n=2); IET Systems Biology (JIF: 1; n=1); Immunological Investigations (JIF: 2.511; n=2); Immunology (JIF: 5.016; n=7); Immunology and Cell Biology (JIF: 3.745; n=2); Immunopharmacology and Immunotoxicology (JIF: 2.352; n=2); Immunotherapy (JIF: 2.964; n=3); Indoor and Built Environment (JIF: 1.9; n=3); Infant Mental Health Journal (JIF: 1.389; n=3); Infection and Drug Resistance (JIF: 2.984; n=1); Infection and Immunity (JIF: 3.201; n=3); Inflammatory Bowel Diseases (JIF: 4.261; n=3); Influenza and Other Respiratory Viruses (JIF: 3.288; n=2); Innate Immunity (JIF: 2.298; n=2); Inquiry: An Interdisciplinary Journal of Philosophy (JIF: 0.938; n=1); Integrated Environmental Assessment and Management (JIF: 3.44; n=4); Integrative Cancer Therapies (JIF: 2.379; n=3); Interactive Cardiovascular and Thoracic Surgery (JIF: 1.675; n=7); Interface Focus (JIF: 3.514; n=1); Internal Medicine Journal (JIF: 1.677; n=12); International Archives of Allergy and Immunology (JIF: 2.917; n=1); International Biodeterioration & Biodegradation (JIF: 4.074; n=1); International Braz J Urol (JIF: 1.342; n=4); International Dental Journal (JIF: 2.038; n=2); International Endodontic Journal (JIF: 3.801; n=18); International Forum of Allergy & Rhinology (JIF: 2.611; n=25); International Immunopharmacology (JIF: 3.943; n=2); International Journal of Audiology (JIF: 1.832; n=6); International Journal of Behavioral Development (JIF: 1.826; n=2); International Journal of Biological Macromolecules (JIF: 5.162; n=2); International Journal of Biometeorology (JIF: 2.68; n=1); International Journal of Cancer (JIF: 5.145; n=33); International Journal of Cardiology (JIF: 3.229; n=1); International Journal of Cardiovascular Imaging (JIF: 1.969; n=1); International Journal of Clinical Oncology (JIF: 2.879; n=1); International Journal of Clinical Practice (JIF: 2.444; n=41); International Journal of Computer Assisted Radiology and Surgery (JIF: 2.473; n=1); International Journal of Dental Hygiene (JIF: 1.229; n=8); International Journal of Dermatology (JIF: 2.067; n=58); International Journal of Developmental Neuroscience (JIF: 1.911; n=3); International Journal of Eating Disorders (JIF: 3.668; n=5); International Journal of Environmental Analytical Chemistry (JIF: 1.431; n=24); International Journal of Environmental Health Research (JIF: 1.916; n=2); International Journal of Environmental Research and Public Health (JIF: 2.849; n=16); International Journal of Environmental Science and Technology (JIF: 2.54; n=2); International Journal of Evidence-Based Healthcare (JIF: 1.93; n=3); International Journal of Experimental Pathology (JIF: 1.672; n=4); International Journal of Geriatric Psychiatry (JIF: 2.675; n=1); International Journal of Gynecology & Obstetrics (JIF: 2.216; n=3); International Journal of Human-Computer Interaction (JIF: 1.713; n=2); International Journal of Hyperthermia (JIF: 3.574; n=12); International Journal of Immunogenetics (JIF: 1.13; n=3); International Journal of Immunopathology and Pharmacology (JIF: 2.209; n=8); International Journal of Impotence Research (JIF: 1.388; n=5); International Journal of Laboratory Hematology (JIF: 2.141; n=12); International Journal of Medical Informatics (JIF: 3.025; n=1); International Journal of Mental Health Nursing (JIF: 2.383; n=7); International Journal of Methods in Psychiatric Research (JIF: 2.341; n=3); International Journal of Neuroscience (JIF: 2.107; n=14); International Journal of Nursing Knowledge (JIF: 1.131; n=4); International Journal of Nursing Practice (JIF: 1.133; n=1); International Journal of Obesity (JIF: 4.419; n=4); International Journal of Older People Nursing (JIF: 1.597; n=1); International Journal of Ophthalmology (JIF: 1.33; n=16); International Journal of Oral Science (JIF: 3.047; n=1); International Journal of Paediatric Dentistry (JIF: 1.993; n=6); International Journal of Pharmaceutics (JIF: 4.845; n=1); International Journal of Psychiatry in Clinical Practice (JIF: 2.144; n=4); International Journal of Psychology (JIF: 1.255; n=4); International Journal of Radiation Biology (JIF: 2.368; n=10); International Journal of Rheumatic Diseases (JIF: 1.98; n=12); International Journal of Sexual Health (JIF: 1.191; n=1); International Journal of Social Psychiatry (JIF: 1.439; n=1); International Journal of Sports Medicine (JIF: 2.556; n=14); International Journal of STD & AIDS (JIF: 1.406; n=5); International Journal of Stroke (JIF: 4.882; n=7); International Journal of Surgery (JIF: 3.357; n=1); International Journal of Surgical Pathology (JIF: 0.872; n=2); International Journal of Toxicology (JIF: 1.255; n=1); International Journal of Urology (JIF: 2.445; n=7); International Nursing Review (JIF: 2.034; n=6); International Ophthalmology (JIF: 1.314; n=1); International Psychogeriatrics (JIF: 2.94; n=5); International Review of Psychiatry (JIF: 2.63; n=1); International Review of Sport and Exercise Psychology (JIF: 14.333; n=1); Interventional Neuroradiology (JIF: 1.479; n=9); Investigacion Clinica (JIF: 0.25; n=2); Investigative and Clinical Urology (JIF: 1.75; n=2); Investigative Ophthalmology & Visual Science (JIF: 3.47; n=1); Iranian Journal of Pediatrics (JIF: 0.37; n=4); Iranian Journal of Radiology (JIF: 0.21; n=1); Iranian Red Crescent Medical Journal (JIF: 0.427; n=4); Irish Journal of Medical Science (JIF: 1.1; n=1); Italian Journal of Pediatrics (JIF: 2.185;

n=1); IUBMB Life (JIF: 3.244; n=10); JACC: Cardiovascular Imaging (JIF: 12.74; n=2); JACC: Cardiovascular Interventions (JIF: 8.432; n=1); JACC: Heart Failure (JIF: 8.75; n=1); JACC: Journal of the American College of Cardiology (JIF: 20.589; n=2); JAIDS Journal of Acquired Immune Deficiency Syndromes (JIF: 3.475; n=4); JAMA (JIF: 45.54; n=2); JAMA Facial Plastic Surgery (JIF: 3.787; n=1); JAMA Internal Medicine (JIF: 18.652; n=1); JAMA Neurology (JIF: 13.608; n=1); JAMA Oncology (JIF: 24.799; n=2); JAMA Ophthalmology (JIF: 6.198; n=1); JAMA Pediatrics (JIF: 13.946; n=1); JAMA Psychiatry (JIF: 17.471; n=2); Japan Journal of Nursing Science (JIF: 0.806; n=4); Japanese Psychological Research (JIF: 1.035; n=1); JCI Insight (JIF: 6.205; n=1); JDDG: Journal der Deutschen Dermatologischen Gesellschaft (JIF: 3.664; n=8); JMIR MHealth and UHealth (JIF: 4.313; n=1); Journal of Addiction Medicine (JIF: 3.014; n=2); Journal of Adolescent and Young Adult Oncology (JIF: 1.465; n=3); Journal of Advanced Nursing (JIF: 2.561; n=36); Journal of Aggression, Maltreatment and Trauma (JIF: 1.03; n=2); Journal of Anatomy (JIF: 2.013; n=17); Journal of Antimicrobial Chemotherapy (JIF: 5.439; n=23); Journal of Applied Behavior Analysis (JIF: 1.54; n=12); Journal of Applied Clinical Medical Physics (JIF: 1.679; n=5); Journal of Applied Ecology (JIF: 5.84; n=18); Journal of Applied Gerontology (JIF: 2.144; n=9); Journal of Applied Microbiology (JIF: 3.066; n=4); Journal of Applied Oral Science (JIF: 1.797; n=6); Journal of Applied Physiology (JIF: 3.044; n=5); Journal of Applied Research in Intellectual Disabilities (JIF: 1.909; n=6); Journal of Applied Social Psychology (JIF: 1.537; n=2); Journal of Applied Toxicology (JIF: 2.997; n=3); Journal of Asthma (JIF: 1.899; n=14); Journal of Athletic Training (JIF: 2.416; n=1); Journal of Attention Disorders (JIF: 2.826; n=5); Journal of Autism and Developmental Disorders (JIF: 3.047; n=6); Journal of Bacteriology (JIF: 3.004; n=7); Journal of Basic Microbiology (JIF: 1.909; n=7); Journal of Behavioral Decision Making (JIF: 1.715; n=2); Journal of Biogeography (JIF: 3.723; n=23); Journal of Biological Chemistry (JIF: 4.238; n=6); Journal of Biological Rhythms (JIF: 3.122; n=3); Journal of Biomaterials Applications (JIF: 2.22; n=11); Journal of Biomechanics (JIF: 2.32; n=1); Journal of Biomedical Nanotechnology (JIF: 4.483; n=1); Journal of Biomedical Optics (JIF: 2.785; n=3); Journal of Biomolecular Structure and Dynamics (JIF: 3.31; n=37); Journal of Biophotonics (JIF: 3.032; n=10); Journal of Black Psychology (JIF: 1.246; n=1); Journal of Bone & Joint Surgery (JIF: 4.578; n=9); Journal of Bone and Mineral Research (JIF: 5.854; n=31); Journal of Breath Research (JIF: 2.929; n=6); Journal of Burn Care & Research (JIF: 1.533; n=2); Journal of Cachexia, Sarcopenia and Muscle (JIF: 9.802; n=6); Journal of Cardiac Surgery (JIF: 1.49; n=32); Journal of Cardiothoracic and Vascular Anesthesia (JIF: 2.258; n=1); Journal of Cardiovascular Electrophysiology (JIF: 2.424; n=24); Journal of Cardiovascular Pharmacology and Therapeutics (JIF: 2.322; n=4); Journal of Cellular and Molecular Medicine (JIF: 4.486; n=20); Journal of Cellular Biochemistry (JIF: 4.237; n=22); Journal of Cellular Physiology (JIF: 5.546; n=37); Journal of Cerebral Blood Flow & Metabolism (JIF: 5.681; n=10); Journal of Chemical Neuroanatomy (JIF: 2.353; n=1); Journal of Chemotherapy (JIF: 1.661; n=1); Journal of Child Health Care (JIF: 1.368; n=6); Journal of Child Neurology (JIF: 1.713; n=5); Journal of Child Psychology and Psychiatry (JIF: 7.035; n=16); Journal of Child Sexual Abuse (JIF: 0.929; n=2); Journal of Clinical and Experimental Neuropsychology (JIF: 1.958; n=4); Journal of Clinical Child & Adolescent Psychology (JIF: 3.656; n=1); Journal of Clinical Investigation (JIF: 11.864; n=3); Journal of Clinical Laboratory Analysis (JIF: 1.54; n=22); Journal of Clinical Microbiology (JIF: 5.897; n=13); Journal of Clinical Nursing (JIF: 1.972; n=38); Journal of Clinical Oncology (JIF: 32.956; n=8); Journal of Clinical Periodontology (JIF: 5.241; n=22); Journal of Clinical Pharmacy & Therapeutics (JIF: 1.833; n=6); Journal of Clinical Psychiatry (JIF: 4.204; n=1); Journal of Clinical Psychology (JIF: 2.138; n=1); Journal of Clinical Psychopharmacology (JIF: 2.7; n=2); Journal of Clinical Ultrasound: Sonography and Other Imaging Techniques (JIF: 0.764; n=1); Journal of Cognitive Neuroscience (JIF: 3.105; n=1); Journal of Cognitive Psychology (JIF: 0.993; n=1); Journal of Community Psychology (JIF: 1.106; n=2); Journal of Comparative Effectiveness Research (JIF: 1.468; n=1); Journal of Comparative Neurology (JIF: 2.801; n=7); Journal of Computer Assisted Tomography (JIF: 1.285; n=2); Journal of Controlled Release (JIF: 7.727; n=1); Journal of Cosmetic and Laser Therapy (JIF: 1.266; n=1); Journal of Cosmetic Dermatology (JIF: 1.621; n=26); Journal of Craniofacial Surgery (JIF: 0.953; n=9); Journal of Cross-Cultural Psychology (JIF: 2.012; n=4); Journal of Cutaneous Medicine and Surgery (JIF: 1.909; n=1); Journal of Cutaneous Pathology (JIF: 1.373; n=6); Journal of Cystic Fibrosis (JIF: 4.759; n=2); Journal of Dental Education (JIF: 1.322; n=7); Journal of Dentistry (JIF: 3.242; n=1); Journal of Dermatological Treatment (JIF: 2.156; n=3); Journal of Diabetes (JIF: 3.28; n=7); Journal of Diabetes Investigation (JIF: 3.761; n=8); Journal of Digestive Diseases (JIF: 1.736; n=2); Journal of Drug Targeting (JIF: 3.38; n=1); Journal of Dual Diagnosis (JIF: 1.231; n=2); Journal of Early Adolescence (JIF: 1.924; n=6); Journal of Ecology (JIF: 5.762; n=22); Journal of Endocrinological Investigation (JIF: 3.397; n=1); Journal of Endocrinology (JIF: 4.041; n=15); Journal of Endourology (JIF: 2.322; n=1); Journal of Endovascular Therapy (JIF: 3.102; n=5); Journal of Environmental Management (JIF: 5.647; n=6); Journal of Environmental Quality (JIF: 2.142; n=1); Journal of Environmental Radioactivity (JIF: 2.161; n=1); Journal of Environmental Science and Health, Part A (JIF: 1.724; n=1); Journal of Environmental Science and Health, Part B (JIF: 1.697; n=1); Journal of Enzyme Inhibition and Medicinal Chemistry (JIF: 4.673; n=5); Journal of Esthetic and Restorative Dentistry (JIF: 1.786; n=9); Journal of Ethnobiology and Ethnomedicine (JIF: 2.264; n=2); Journal of Ethnopharmacology (JIF: 3.69; n=1); Journal of Evaluation in Clinical Practice (JIF: 1.681; n=5); Journal of Experimental Psychology: General (JIF: 3.169; n=1); Journal of Experimental Psychopathology (JIF: 1.34; n=1); Journal of Experimental Social Psychology (JIF: 3.254; n=1); Journal of Extracellular Vesicles (JIF: 14.976; n=1); Journal of Family Nursing (JIF: 1.889; n=1); Journal of Flood Risk Management (JIF: 3.066; n=7); Journal of Forensic Sciences (JIF: 1.441; n=5); Journal of Gastroenterology and Hepatology (JIF: 3.437; n=25); Journal of Gastrointestinal Oncology (JIF: 2.536; n=1); Journal of Gene Medicine (JIF: 3.258; n=4); Journal of General Psychology (JIF: 0.409; n=2); Journal of Genetics and Genomics (JIF: 5.065; n=1); Journal of Geriatric Psychiatry and Neurology (JIF: 2.125; n=4); Journal of Hand Surgery (European Volume) (JIF: 2.29; n=9); Journal of Health Psychology (JIF: 2.503; n=9); Journal of Hepato-biliary-pancreatic Sciences (JIF: 4.16; n=5); Journal of Heredity (JIF: 2.809; n=1); Journal of Hospital Infection (JIF: 3.271; n=2); Journal of Human Genetics (JIF: 2.831; n=1); Journal of Human Hypertension (JIF: 2.26; n=1); Journal of Human Lactation (JIF: 2.205; n=7); Journal of Human Nutrition and Dietetics (JIF: 3.146; n=10); Journal of Humanistic Psychology (JIF: 1.902; n=4); Journal of Hypertension (JIF: 4.171; n=15); Journal of Industrial Ecology (JIF: 6.539; n=14); Journal of Infection in Developing Countries (JIF: 0.703; n=1); Journal of Intellectual & Developmental Disability (JIF: 1.148; n=1); Journal of Intensive Care Medicine (JIF: 3.142; n=8); Journal of Internal Medicine (JIF: 6.871; n=5); Journal of International Medical Research (JIF: 1.287; n=31); Journal of Interventional Cardiology (JIF: 1.758; n=11); Journal of Investigative Dermatology (JIF: 7.143; n=9); Journal of Investigative Psychology and Offender Profiling (JIF: 0.783; n=2); Journal of Investigative Surgery (JIF: 1.685; n=13); Journal of Knee Surgery (JIF: 1.986; n=6); Journal of Korean Medical Science (JIF: 1.705; n=2); Journal of Land Use Science (JIF: 1.106; n=1); Journal of Language and Social Psychology (JIF: 1.162; n=1); Journal of Laparoendoscopic and Advanced Surgical Techniques (JIF: 1.31; n=2); Journal of Leukocyte Biology (JIF: 3.757; n=1); Journal of Liposome Research (JIF: 2.455; n=1); Journal of Lower Genital Tract Disease (JIF: 1.306; n=1); Journal of Magnetic Resonance Imaging (JIF: 3.954; n=38); Journal of Managerial Psychology (JIF: 1.38; n=5); Journal of Medical Imaging and Radiation Oncology (JIF: 1.283; n=12); Journal of Medical Internet Research (JIF: 5.034; n=1); Journal of Medical Virology (JIF: 2.021; n=8); Journal of Memory and Language (JIF: 3.893; n=1); Journal of Mental Health (JIF: 2.703; n=4); Journal of Microencapsulation (JIF: 2.287; n=1); Journal of Microscopy (JIF: 1.575; n=6); Journal of Midwifery & Women's Health (JIF: 1.742; n=2); Journal of Minimal Access Surgery (JIF: 1.168; n=1); Journal of Molecular Endocrinology (JIF: 3.562; n=8); Journal of Molecular Recognition (JIF: 2.214; n=1); Journal of Morphology (JIF: 1.563; n=4); Journal of Motor Behavior (JIF: 1.279; n=3); Journal of Nanobiotechnology (JIF: 6.518; n=1); Journal of Natural History (JIF: 1.032; n=4); Journal of Neural Engineering (JIF: 4.141; n=29); Journal of Neurochemistry (JIF: 4.066; n=13); Journal of Neuroendocrinology (JIF: 2.886; n=6); Journal of NeuroEngineering and Rehabilitation (JIF: 3.519; n=1); Journal of Neurological Surgery Part A: Central European Neurosurgery (JIF: 0.905; n=3); Journal of Neurological Surgery Part B: Skull Base (JIF: 1.397; n=3); Journal of Neurophysiology (JIF: 2.225; n=8); Journal of Neuropsychology (JIF: 2.333; n=1); Journal of Neuroscience Research (JIF: 4.699; n=11); Journal of Neurosurgery (JIF: 3.968; n=2); Journal of Neurotrauma (JIF: 3.793; n=3); Journal of Nursing Administration (JIF: 1.274; n=1); Journal of Nursing Management (JIF: 2.243; n=12); Journal of Obstetrics and

Gynaecology (JIF: 0.807; n=11); Journal of Obstetrics and Gynaecology Research (JIF: 1.392; n=19); Journal of Occupational Health Psychology (JIF: 7.365; n=1); Journal of Oceanology and Limnology (JIF: 0.617; n=1); Journal of Oncology Pharmacy Practice (JIF: 1.85; n=4); Journal of Oncology Practice (JIF: 3.551; n=2); Journal of Ophthalmology (JIF: 1.447; n=1); Journal of Oral Microbiology (JIF: 3.939; n=2); Journal of Oral Pathology and Medicine (JIF: 2.495; n=10); Journal of Oral Rehabilitation (JIF: 2.304; n=17); Journal of Organizational Behavior (JIF: 5.026; n=8); Journal of Orthopaedic Research (JIF: 2.728; n=5); Journal of Orthopaedic Surgery (JIF: 1.095; n=23); Journal of Paediatrics and Child Health (JIF: 1.71; n=13); Journal of Palliative Medicine (JIF: 2.085; n=1); Journal of Parenteral and Enteral Nutrition (JIF: 2.853; n=1); Journal of Pediatric Hematology/Oncology Nursing (JIF: 1.442; n=2); Journal of Pediatric Infectious Diseases (JIF: 0.227; n=2); Journal of Peptide Science (JIF: 1.877; n=5); Journal of Perinatology (JIF: 1.967; n=1); Journal of Periodontal Research (JIF: 2.926; n=15); Journal of Periodontology (JIF: 3.742; n=9); Journal of Personality (JIF: 3.667; n=6); Journal of Personality Assessment (JIF: 2.185; n=1); Journal of Pharmaceutical and Biomedical Analysis (JIF: 3.209; n=1); Journal of Pharmaceutical Sciences (JIF: 2.997; n=1); Journal of Pharmacological and Toxicological Methods (JIF: 2.252; n=1); Journal of Pharmacology and Experimental Therapeutics (JIF: 3.561; n=3); Journal of Pharmacy and Pharmacology (JIF: 2.571; n=22); Journal of Pineal Research (JIF: 14.528; n=6); Journal of Plastic Surgery and Hand Surgery (JIF: 1.235; n=3); Journal of Prosthodontics (JIF: 2.187; n=11); Journal of Psychiatric and Mental Health Nursing (JIF: 1.947; n=9); Journal of Psychiatric Research (JIF: 3.745; n=2); Journal of Psychoactive Drugs (JIF: 1.859; n=1); Journal of Psychoeducational Assessment (JIF: 1.256; n=4); Journal of Psychology: Interdisciplinary and Applied (JIF: 1.548; n=3); Journal of Psychopharmacology (JIF: 3.121; n=9); Journal of Psychosomatic Obstetrics & Gynecology (JIF: 2; n=2); Journal of Radiological Protection (JIF: 1.261; n=6); Journal of Receptors and Signal Transduction (JIF: 1.466; n=1); Journal of Reconstructive Microsurgery (JIF: 1.841; n=5); Journal of Renal Care (JIF: 0.961; n=2); Journal of Reproductive and Infant Psychology (JIF: 1.188; n=2); Journal of Research on Adolescence (JIF: 1.905; n=3); Journal of School Nursing (JIF: 1.694; n=1); Journal of Sex & Marital Therapy (JIF: 1.775; n=2); Journal of Sleep Research (JIF: 3.623; n=12); Journal of Spinal Cord Medicine (JIF: 1.816; n=3); Journal of Sport & Exercise Psychology (JIF: 2.239; n=1); Journal of Sport and Health Science (JIF: 5.2; n=1); Journal of Sports Science and Medicine (JIF: 1.806; n=1); Journal of Sports Sciences (JIF: 2.597; n=7); Journal of Strength and Conditioning Research (JIF: 2.973; n=1); Journal of Surgical Oncology (JIF: 2.771; n=12); Journal of Sustainable Forestry (JIF: 1.272; n=5); Journal of Telemedicine and Telecare (JIF: 2.616; n=3); Journal of the American Academy of Audiology (JIF: 1.657; n=1); Journal of the American Academy of Dermatology (JIF: 8.277; n=1); Journal of the American Academy of Orthopaedic Surgeons (JIF: 2.286; n=4); Journal of the American Heart Association (JIF: 4.605; n=8); Journal of the American Nutrition Association (JIF: 2.297; n=2); Journal of the American Podiatric Medical Association (JIF: 0.649; n=1); Journal of the American Psychiatric Nurses Association (JIF: 1.652; n=2); Journal of the American Society of Nephrology (JIF: 9.274; n=5); Journal of the American Water Resources Association (JIF: 2.472; n=4); Journal of the European Academy of Dermatology and Venereology (JIF: 5.248; n=23); Journal of the Experimental Analysis of Behavior (JIF: 2.146; n=3); Journal of the International Neuropsychological Society (JIF: 2.576; n=2); Journal of the Peripheral Nervous System (JIF: 2.466; n=2); Journal of the Royal Society of Medicine (JIF: 5.238; n=1); Journal of Theoretical Biology (JIF: 2.327; n=2); Journal of Thoracic Disease (JIF: 2.046; n=7); Journal of Thrombosis and Haemostasis (JIF: 4.157; n=29); Journal of Tissue Engineering (JIF: 5.352; n=3); Journal of Tissue Engineering and Regenerative Medicine (JIF: 3.078; n=2); Journal of Toxicology and Environmental Health - Part A: Current Issues (JIF: 2.653; n=3); Journal of Toxicology and Environmental Health - Part B: Critical Reviews (JIF: 6.105; n=1); Journal of Transcultural Nursing (JIF: 1.441; n=7); Journal of Trauma and Dissociation (JIF: 1.859; n=3); Journal of Traumatic Stress (JIF: 1.926; n=2); Journal of Ultrasound in Medicine (JIF: 1.759; n=14); Journal of Urology (JIF: 5.925; n=1); Journal of Viral Hepatitis (JIF: 3.561; n=7); Journal of Virology (JIF: 4.501; n=19); Journal of Vision (JIF: 2.145; n=2); Journal of Vocational Behavior (JIF: 4.075; n=1); Journal of Wound Care (JIF: 1.798; n=2); Jundishapur Journal of Microbiology (JIF: 0.593; n=1); Kardiologia Polska (JIF: 1.874; n=3); Klinische Monatsblätter für Augenheilkunde (JIF: 0.605; n=2); Klinische Pädiatrie (JIF: 0.882; n=1); Korean Journal of Radiology (JIF: 3.179; n=3); La Medicina del Lavoro (JIF: 0.978; n=1); Laboratory Investigation (JIF: 4.197; n=1); Land Degradation & Development (JIF: 3.775; n=1); Language Learning and Development (JIF: 1.651; n=1); Language, Cognition and Neuroscience (JIF: 1.925; n=3); Laryngo-Rhino-Otologie (JIF: 0.972; n=1); Lasers in Surgery and Medicine (JIF: 3.02; n=9); Laterality: Asymmetries of Body, Brain and Cognition (JIF: 0.938; n=1); Letters in Drug Design & Discovery (JIF: 1.169; n=1); Leukemia (JIF: 8.665; n=2); Leukemia & Lymphoma (JIF: 2.969; n=15); Life Sciences (JIF: 3.647; n=4); Limnology and Oceanography (JIF: 3.778; n=9); Limnology and Oceanography: Methods (JIF: 2.458; n=7); Lipids in Health and Disease (JIF: 2.906; n=2); Liver Cancer (JIF: 9.72; n=1); Liver International (JIF: 5.175; n=24); Liver Transplantation (JIF: 4.57; n=17); Local Environment (JIF: 1.856; n=3); Logopedics Phoniatrics Vocology (JIF: 0.714; n=1); Luminescence: the Journal of Biological and Chemical Luminescence (JIF: 1.855; n=3); Lupus (JIF: 2.251; n=9); LUTS: Lower Urinary Tract Symptoms (JIF: 1.205; n=4); MAbs (JIF: 4.634; n=1); Magnetic Resonance in Medicine (JIF: 3.635; n=40); Malaria Journal (JIF: 2.631; n=1); Malawi Medical Journal (JIF: 0.598; n=1); Marine Biodiversity (JIF: 1.487; n=1); Marine Drugs (JIF: 4.073; n=1); Marine Ecology (JIF: 1.359; n=1); Marine Ecology Progress Series (JIF: 2.326; n=2); Marine Environmental Research (JIF: 2.727; n=1); Mayo Clinic Proceedings (JIF: 6.942; n=5); mBio (JIF: 6.784; n=6); Measurement and Evaluation in Counseling and Development (JIF: 1.851; n=1); Media Psychology (JIF: 2.397; n=1); Medical Journal of Australia (JIF: 6.112; n=3); Medical Physics (JIF: 3.317; n=17); Medical Science Monitor (JIF: 1.918; n=10); Medical Teacher (JIF: 2.654; n=8); Medicinal Research Reviews (JIF: 9.3; n=1); Medicine (JIF: 1.552; n=14); Medicine & Science in Sports & Exercise (JIF: 4.029; n=1); Memory (JIF: 1.895; n=3); Mental Health and Physical Activity (JIF: 1.727; n=1); Metabolites (JIF: 4.097; n=2); Metabolomics (JIF: 2.881; n=1); Methods in Ecology and Evolution (JIF: 6.511; n=22); Methods of Information in Medicine (JIF: 1.574; n=3); Microbial Cell Factories (JIF: 4.187; n=1); Microbiological Research (JIF: 3.97; n=1); Microbiology and Immunology (JIF: 1.566; n=2); Microbiology Spectrum (JIF: 5.465; n=1); MicrobiologyOpen (JIF: 3.142; n=5); Microcirculation (JIF: 2.11; n=3); Microorganisms (JIF: 4.152; n=1); Microscopy Research and Technique (JIF: 2.117; n=6); Microsurgery (JIF: 1.996; n=1); Minimally Invasive Therapy and Allied Technologies (JIF: 1.028; n=3); Mitochondrial DNA Part A (JIF: 1.073; n=1); Mitochondrial DNA Part B (JIF: 0.885; n=11); Mobile DNA (JIF: 3.161; n=1); Modern Pathology (JIF: 5.988; n=1); Modern Rheumatology (JIF: 2.113; n=7); Molecular & Cellular Proteomics (JIF: 4.87; n=2); Molecular and Cellular Biochemistry (JIF: 2.795; n=2); Molecular and Cellular Biology (JIF: 3.611; n=2); Molecular Biology and Evolution (JIF: 11.062; n=1); Molecular Biology of the Cell (JIF: 3.791; n=3); Molecular Biology Reports (JIF: 1.402; n=2); Molecular Brain (JIF: 4.686; n=1); Molecular Cancer (JIF: 15.302; n=1); Molecular Cancer Research (JIF: 4.63; n=2); Molecular Carcinogenesis (JIF: 3.825; n=13); Molecular Cytogenetics (JIF: 1.233; n=2); Molecular Ecology (JIF: 5.163; n=60); Molecular Ecology Resources (JIF: 6.286; n=21); Molecular Genetics & Genomic Medicine (JIF: 1.995; n=3); Molecular Genetics and Genomics (JIF: 2.797; n=1); Molecular Human Reproduction (JIF: 3.636; n=4); Molecular Immunology (JIF: 3.641; n=1); Molecular Medicine Reports (JIF: 2.1; n=1); Molecular Microbiology (JIF: 3.418; n=13); Molecular Neurodegeneration (JIF: 9.599; n=1); Molecular Omics (JIF: 2.273; n=1); Molecular Oncology (JIF: 6.574; n=8); Molecular Oral Microbiology (JIF: 2.905; n=1); Molecular Pain (JIF: 2.696; n=2); Molecular Phylogenetics and Evolution (JIF: 3.496; n=2); Molecular Psychiatry (JIF: 12.384; n=3); Molecular Reproduction & Development (JIF: 2.823; n=1); Molecular Systems Biology (JIF: 8.991; n=2); Molecular Therapy - Nucleic Acids (JIF: 7.032; n=1); Molecular Therapy (JIF: 8.986; n=2); Molecular Vision (JIF: 2.202; n=1); mSphere (JIF: 4.282; n=4); mSystems (JIF: 6.633; n=1); Multiple Sclerosis Journal (JIF: 5.412; n=13); Multivariate Behavioral Research (JIF: 2.75; n=1); Muscle and Nerve (JIF: 2.505; n=6); Mycobiology (JIF: 1.416; n=2); Nanomedicine (JIF: 4.3; n=5); Nanotoxicology (JIF: 4.925; n=3); Natural Product Communications (JIF: 0.468; n=6); Natural Product Research (JIF: 2.158; n=34); Natural Resource Modeling (JIF: 0.915; n=2); Nature Biotechnology (JIF: 36.558; n=1); Nature Cell Biology (JIF: 20.042; n=1); Nature Climate Change (JIF: 20.893; n=1); Nature Ecology & Evolution (JIF: 12.541; n=4); Nature Human Behaviour (JIF: 12.282; n=4); Nature Immunology (JIF: 20.479; n=1); Nature Methods (JIF: 30.822; n=5); Nature Microbiology (JIF: 15.54; n=1); Nature Protocols (JIF: 10.419; n=2); Nature Reviews Cancer (JIF: 53.03; n=1); Nature Reviews Genetics (JIF: 33.133;

n=1); Nature Reviews Microbiology (JIF: 34.209; n=1); Neoplasma (JIF: 1.721; n=1); Nephrology (JIF: 1.749; n=12); Nephrology Dialysis Transplantation (JIF: 4.531; n=18); Neural Plasticity (JIF: 3.093; n=1); Neurocase (JIF: 0.83; n=3); Neurogastroenterology and Motility (JIF: 2.946; n=9); NeuroImage (JIF: 5.902; n=4); NeuroImage: Clinical (JIF: 4.35; n=3); Neurological Research (JIF: 2.401; n=7); Neuropathology (JIF: 1.758; n=2); Neuropathology and Applied Neurobiology (JIF: 7.5; n=3); Neuropediatrics (JIF: 1.549; n=2); Neuropeptides (JIF: 2.411; n=1); Neuropsychologia (JIF: 2.652; n=1); Neuropsychological Rehabilitation (JIF: 2.556; n=3); Neuropsychopharmacology (JIF: 6.751; n=4); Neuroradiology (JIF: 2.238; n=1); Neurorehabilitation and Neural Repair (JIF: 3.982; n=8); Neuroscience Research (JIF: 2.645; n=1); Neurotoxicity Research (JIF: 2.992; n=1); New Directions for Child and Adolescent Development (JIF: 1.333; n=3); New England Journal of Medicine (JIF: 74.699; n=1); NMR in Biomedicine (JIF: 3.221; n=8); Nordic Journal of Psychiatry (JIF: 1.78; n=1); Nordic Psychology (JIF: 1.462; n=1); npj Primary Care Respiratory Medicine (JIF: 3.231; n=1); npj Vaccines (JIF: 5.699; n=2); Nuclear Medicine Communications (JIF: 1.334; n=3); Nucleic Acids Research (JIF: 11.501; n=86); Nuklearmedizin (JIF: 1.085; n=1); Nurse Education Today (JIF: 2.49; n=1); Nurse Educator (JIF: 1.454; n=1); Nursing & Health Sciences (JIF: 1.269; n=6); Nursing Ethics (JIF: 2.597; n=1); Nursing in Critical Care (JIF: 2.205; n=2); Nursing Inquiry (JIF: 1.894; n=2); Nursing Research (JIF: 1.881; n=1); Nutrition and Cancer (JIF: 2.363; n=4); Nutrition Research (JIF: 2.767; n=1); Nutrition, Metabolism and Cardiovascular Diseases (JIF: 3.7; n=1); Nutritional Neuroscience (JIF: 4.028; n=2); Obesity (JIF: 3.742; n=5); Obesity Reviews (JIF: 7.31; n=8); Obesity Surgery (JIF: 3.412; n=4); Obstetrics & Gynecology (JIF: 5.524; n=12); Ocean and Coastal Management (JIF: 2.482; n=1); Ocular Immunology and Inflammation (JIF: 2.112; n=14); Oecologia (JIF: 2.654; n=5); Oikos (JIF: 3.37; n=26); OMEGA - Journal of Death and Dying (JIF: 1.347; n=8); Oncogene (JIF: 7.971; n=5); Oncogenesis (JIF: 6.119; n=3); OncoImmunology (JIF: 5.869; n=6); Oncotarget (JIF: 5.168; n=17); Open Biology (JIF: 4.931; n=1); Ophthalmic and Physiological Optics (JIF: 2.624; n=3); Ophthalmic Epidemiology (JIF: 1.5; n=3); Ophthalmic Genetics (JIF: 1.308; n=4); Oral Diseases (JIF: 2.613; n=23); Orthodontics and Craniofacial Research (JIF: 1.455; n=2); Orthopaedic Surgery (JIF: 1.718; n=1); Osteoarthritis and Cartilage (JIF: 4.793; n=1); Osteoporosis International (JIF: 3.864; n=1); OTJR: Occupation, Participation and Health (JIF: 1.22; n=2); Otolaryngology - Head and Neck Surgery (JIF: 2.341; n=8); Oxidative Medicine and Cellular Longevity (JIF: 5.076; n=1); Ozone: Science and Engineering (JIF: 2.082; n=2); Pacing and Clinical Electrophysiology (JIF: 1.303; n=16); Paediatric & Perinatal Epidemiology (JIF: 2.917; n=4); PAIN (JIF: 5.483; n=8); Pain Medicine (JIF: 2.513; n=10); Pain Practice (JIF: 2.258; n=8); Pancreatology (JIF: 3.629; n=1); Parasite (JIF: 2.05; n=2); Parasite Immunology (JIF: 2.054; n=1); Parasites & Vectors (JIF: 2.824; n=3); Parasitology (JIF: 2.783; n=3); Parenting: Science and Practice (JIF: 1.156; n=1); Pathogens and Disease (JIF: 2.166; n=2); Pathogens and Global Health (JIF: 2.42; n=2); Pathology International (JIF: 2.11; n=11); Pathology Research and Practice (JIF: 2.05; n=1); Pediatric Allergy and Immunology (JIF: 4.699; n=24); Pediatric and Developmental Pathology (JIF: 0.885; n=3); Pediatric Anesthesia (JIF: 2.311; n=5); Pediatric Blood and Cancer (JIF: 2.355; n=41); Pediatric Critical Care Medicine (JIF: 2.854; n=1); Pediatric Dermatology (JIF: 1.164; n=17); Pediatric Diabetes (JIF: 3.052; n=8); Pediatric Hematology and Oncology (JIF: 1.232; n=2); Pediatric Infectious Disease Journal (JIF: 2.126; n=1); Pediatric Obesity (JIF: 3.429; n=3); Pediatric Pulmonology (JIF: 2.534; n=32); Pediatric Transplantation (JIF: 1.425; n=15); Pediatrics International (JIF: 1.139; n=19); Peptide Science (JIF: 1.865; n=1); Perception (JIF: 1.217; n=1); Perceptual and Motor Skills (JIF: 1.245; n=6); Perfusion (JIF: 1.234; n=11); Peritoneal Dialysis International (JIF: 1.768; n=8); Personality and Individual Differences (JIF: 2.311; n=1); Personality and Mental Health (JIF: 1.929; n=1); Personality and Social Psychology Bulletin (JIF: 2.97; n=6); Personalized Medicine (JIF: 2.318; n=1); Personnel Psychology (JIF: 6.571; n=2); Personnel Review (JIF: 2.074; n=2); Perspectives in Psychiatric Care (JIF: 1.273; n=10); Perspectives on Psychological Science (JIF: 8.275; n=3); Pharmaceutical Biology (JIF: 2.971; n=5); Pharmaceutical Development and Technology (JIF: 2.169; n=9); Pharmaceutical Statistics (JIF: 1.374; n=1); Pharmaceutics (JIF: 4.421; n=2); Pharmacoepidemiology and Drug Safety (JIF: 2.918; n=2); Pharmacogenomics (JIF: 2.339; n=3); Pharmacological Reports (JIF: 2.754; n=1); Pharmacological Research (JIF: 5.893; n=2); Pharmacopsychiatry (JIF: 4.34; n=3); Pharmacotherapy: The Journal of Human Pharmacology and Drug Therapy (JIF: 3.473; n=10); Philosophical Psychology (JIF: 0.785; n=1); Philosophical Transactions of the Royal Society B: Biological Sciences (JIF: 5.68; n=10); Phlebology (JIF: 1.914; n=5); Photochemistry & Photobiology (JIF: 2.721; n=2); Photodermatology, Photoimmunology & Photomedicine (JIF: 2.387; n=3); Physical Biology (JIF: 2; n=4); Physics in Medicine and Biology (JIF: 2.883; n=64); Physikalische Medizin, Rehabilitationsmedizin, Kurortmedizin (JIF: 0.414; n=2); Physiological Genomics (JIF: 2.749; n=1); Physiological Measurement (JIF: 2.309; n=24); Physiotherapy Theory and Practice (JIF: 1.624; n=5); Phytotherapy Research (JIF: 4.087; n=29); Pigment Cell & Melanoma Research: The Official Journal of the International Federation of Pigment Cell Societies and the Society for Melanoma Research (JIF: 3.683; n=7); Placenta (JIF: 3.177; n=1); Planta Medica (JIF: 2.687; n=11); Plastic and Reconstructive Surgery (JIF: 4.209; n=65); Platelets (JIF: 3.378; n=5); PLoS Biology (JIF: 7.076; n=16); PLOS Computational Biology (JIF: 4.7; n=17); PLOS Genetics (JIF: 5.174; n=8); PLOS Medicine (JIF: 10.5; n=4); PLoS Neglected Tropical Diseases (JIF: 3.885; n=8); PLOS Pathogens (JIF: 6.218; n=3); PM&R: The Journal of Injury, Function and Rehabilitation (JIF: 1.821; n=9); Polish Archives of Internal Medicine (JIF: 3.007; n=5); Political Psychology (JIF: 3.265; n=3); Population Ecology (JIF: 1.297; n=3); Postgraduate Medicine (JIF: 2.464; n=10); Prehospital Emergency Care (JIF: 2.29; n=2); Prenatal Diagnosis (JIF: 2.425; n=20); Preparative Biochemistry and Biotechnology (JIF: 1.415; n=6); Primary Health Care Research & Development (JIF: 1.11; n=2); Prion (JIF: 1.952; n=2); Process Biochemistry (JIF: 2.952; n=1); Process Safety and Environmental Protection (JIF: 4.966; n=1); Progress in Transplantation (JIF: 0.81; n=2); Prostate (JIF: 3.279; n=7); Prosthetics and Orthotics International (JIF: 1.363; n=1); Protein & Cell (JIF: 10.164; n=2); Protein Engineering Design and Selection (JIF: 1.774; n=1); Protein Science (JIF: 3.876; n=2); Proteins: Structure, Function, and Bioinformatics (JIF: 2.828; n=16); Proteomics – Clinical Applications (JIF: 3.567; n=1); Proteomics (JIF: 3.254; n=1); Psicothema (JIF: 2.632; n=1); PsyCh Journal (JIF: 0.785; n=3); Psychiatric Services (JIF: 2.539; n=4); Psychiatry, Psychology and Law (JIF: 0.664; n=3); Psycho-Oncology (JIF: 3.006; n=5); Psychogeriatrics (JIF: 1.75; n=4); Psychological Medicine (JIF: 5.813; n=11); Psychological Reports (JIF: 1.535; n=7); Psychological Research (JIF: 2.419; n=1); Psychological Science (JIF: 5.389; n=4); Psychology & Marketing (JIF: 2.37; n=4); Psychology and Health (JIF: 2.534; n=11); Psychology and Sexuality (JIF: 1.292; n=1); Psychology in the Schools (JIF: 1.134; n=8); Psychology of Music (JIF: 1.712; n=2); Psychology of Women Quarterly (JIF: 2.444; n=4); Psychology, Crime and Law (JIF: 1.193; n=2); Psychophysiology (JIF: 3.692; n=5); Psychosis (JIF: 1.085; n=3); Psychosomatic Medicine (JIF: 3.702; n=2); Psychotherapy Research (JIF: 2.984; n=6); Public Health Nursing (JIF: 1.13; n=10); Pulmonary Circulation (JIF: 2.205; n=2); Quantitative Imaging in Medicine and Surgery (JIF: 3.226; n=7); Quarterly Journal of Experimental Psychology (JIF: 2.077; n=15); Redox Report (JIF: 2.753; n=1); Regenerative Medicine (JIF: 2.598; n=1); Regional Anesthesia and Pain Medicine (JIF: 7.015; n=1); Regulatory Toxicology and Pharmacology (JIF: 2.652; n=1); Rejuvenation Research (JIF: 3.811; n=1); Renal Failure (JIF: 1.985; n=14); Reproduction (JIF: 3.206; n=24); Reproductive Biology and Endocrinology (JIF: 3.235; n=1); Reproductive Sciences (JIF: 2.616; n=2); Research Quarterly for Exercise and Sport (JIF: 1.883; n=1); Resources, Conservation and Recycling (JIF: 8.086; n=1); Respiratory Care (JIF: 2.066; n=5); Respiratory Physiology and Neurobiology (JIF: 1.591; n=1); Respirology (JIF: 4.88; n=3); Restoration Ecology (JIF: 2.721; n=12); Retrovirology (JIF: 4.183; n=1); Review of General Psychology (JIF: 2.364; n=1); Reviews in Medical Virology (JIF: 4.221; n=4); Revista Brasileira de Psiquiatria (JIF: 1.904; n=2); Revista Clínica Española (JIF: 1.304; n=1); Revista da Associacao Medica Brasileira (JIF: 0.915; n=5); Revista da Escola de Enfermagem da USP (JIF: 0.798; n=1); Revista da Sociedade Brasileira de Medicina Tropical (JIF: 1.358; n=6); Revista de Psiquiatria Clinica (JIF: 0.586; n=1); Rheumatology (JIF: 5.606; n=33); Rheumatology International (JIF: 1.984; n=1); River Research and Applications (JIF: 1.916; n=9); RNA Biology (JIF: 5.35; n=5); RöFo - Fortschritte Auf Dem Gebiet der Röntgenstrahlen und der Bildgebenden Verfahren (JIF: 1.867; n=4); Saudi Medical Journal (JIF: 1.195; n=1); Scandinavian Journal of Caring Sciences (JIF: 1.525; n=3); Scandinavian Journal of Clinical and Laboratory Investigation (JIF: 1.475; n=3); Scandinavian Journal of Gastroenterology (JIF: 2.13; n=13); Scandinavian Journal of Immunology (JIF: 2.717; n=8); Scandinavian Journal of Medicine & Science in Sports (JIF: 3.255; n=24); Scandinavian Journal of Occupational Therapy (JIF: 1.347;

n=3); Scandinavian Journal of Psychology (JIF: 1.57; n=4); Scandinavian Journal of Rheumatology (JIF: 3.025; n=1); Scandinavian Journal of Surgery (JIF: 1.95; n=5); Schizophrenia Research (JIF: 3.759; n=1); Science China Life Sciences (JIF: 4.611; n=1); Science of the Total Environment (JIF: 6.551; n=14); Scottish Medical Journal (JIF: 0.537; n=2); Self and Identity (JIF: 1.797; n=1); Seminars in Liver Disease (JIF: 4.457; n=1); Seminars in Ophthalmology (JIF: 1.205; n=4); Sexual and Relationship Therapy (JIF: 1.125; n=1); Sexual Health (JIF: 1.584; n=2); Sexually Transmitted Diseases (JIF: 2.74; n=3); Sexually Transmitted Infections (JIF: 3.396; n=1); Signal Transduction and Targeted Therapy (JIF: 13.493; n=3); SLAS Discovery (JIF: 2.297; n=4); Sleep (JIF: 4.805; n=2); Sleep Medicine (JIF: 3.038; n=1); Small Group Research (JIF: 1.745; n=1); Social Development (JIF: 1.552; n=6); Social Neuroscience (JIF: 2.248; n=3); Social Psychiatry and Psychiatric Epidemiology (JIF: 3.335; n=1); Social Psychological and Personality Science (JIF: 4.385; n=7); Social Psychology of Education (JIF: 1.619; n=1); Soil and Sediment Contamination (JIF: 1.25; n=5); South African Journal of Psychology (JIF: 0.898; n=1); Spatial Cognition and Computation (JIF: 1.767; n=1); Spinal Cord (JIF: 1.773; n=3); Spine (JIF: 2.646; n=1); Sports Biomechanics (JIF: 2.023; n=8); Stem Cell Research (JIF: 4.489; n=1); Stem Cell Research & Therapy (JIF: 5.116; n=3); Stem Cells (JIF: 6.022; n=6); Stem Cells and Development (JIF: 3.082; n=2); Stem Cells Translational Medicine (JIF: 6.429; n=2); Stress and Health (JIF: 2.35; n=8); Stroke (JIF: 7.19; n=8); Suicide and Life-Threatening Behavior (JIF: 3.867; n=1); Surgical and Radiologic Anatomy (JIF: 1.092; n=1); Surgical Endoscopy (JIF: 3.149; n=1); Surgical Innovation (JIF: 1.458; n=3); Sustainability (JIF: 2.576; n=16); Synapse (JIF: 2.318; n=3); Systematic Biology (JIF: 10.408; n=1); Systems Biology in Reproductive Medicine (JIF: 1.946; n=1); Teaching of Psychology (JIF: 1.053; n=1); Technology in Cancer Research & Treatment (JIF: 2.074; n=21); The American Journal of Dermatopathology (JIF: 1.102; n=1); The American Journal of Gastroenterology (JIF: 10.171; n=1); The American Journal of Tropical Medicine and Hygiene (JIF: 2.126; n=1); The Anatomical Record (JIF: 1.634; n=8); The Annals of the Royal College of Surgeons of England (JIF: 1.22; n=7); The Australian Journal of Rural Health (JIF: 1.464; n=4); The Bone & Joint Journal (JIF: 4.306; n=11); The Breast Journal (JIF: 1.991; n=4); The British Journal of Psychiatry (JIF: 7.85; n=5); The British Journal of Radiology (JIF: 2.196; n=29); The Cleft Palate-Craniofacial Journal (JIF: 1.347; n=8); The Clinical Neuropsychologist (JIF: 2.232; n=6); The Clinical Respiratory Journal (JIF: 1.514; n=8); The EMBO Journal (JIF: 9.889; n=3); The FASEB Journal (JIF: 4.966; n=3); The FEBS Journal (JIF: 4.392; n=22); The International Journal of Aerospace Psychology (JIF: 1.111; n=1); The International Journal of Artificial Organs (JIF: 1.337; n=3); The International Journal of Biochemistry & Cell Biology (JIF: 3.673; n=1); The International Journal of Lower Extremity Wounds (JIF: 1.38; n=6); The International Journal of Medical Robotics and Computer Assisted Surgery (JIF: 2.015; n=1); The International Journal of Psychiatry in Medicine (JIF: 0.724; n=1); The ISME Journal (JIF: 9.18; n=1); The Journal of Antibiotics (JIF: 2.668; n=1); The Journal of Biochemistry (JIF: 2.476; n=1); The Journal of Cell Biology (JIF: 8.811; n=1); The Journal of Clinical Hypertension (JIF: 2.719; n=18); The Journal of Dermatology (JIF: 3.072; n=29); The Journal of Forensic Psychiatry & Psychology (JIF: 1.061; n=1); The Journal of Immunology (JIF: 4.886; n=2); The Journal of Maternal-Fetal & Neonatal Medicine (JIF: 1.737; n=22); The Journal of Nervous and Mental Disease (JIF: 1.647; n=1); The Journal of Neuropsychiatry and Clinical Neurosciences (JIF: 2.192; n=1); The Journal of Neuroscience (JIF: 5.673; n=11); The Journal of Nutritional Biochemistry (JIF: 4.873; n=1); The Journal of Pathology (JIF: 5.979; n=6); The Journal of Physiology (JIF: 4.547; n=8); The Journal of Positive Psychology (JIF: 3.828; n=3); The Journal of Rheumatology (JIF: 3.35; n=9); The Journal of Sex Research (JIF: 3.683; n=7); The Journal of Sexual Medicine (JIF: 3.293; n=12); The Journal of Social Psychology (JIF: 1.241; n=1); The Journal of Steroid Biochemistry and Molecular Biology (JIF: 3.813; n=1); The Journal of Vascular Access (JIF: 1.223; n=7); The Lancet (JIF: 60.392; n=1); The Lancet Respiratory Medicine (JIF: 25.094; n=1); The Laryngoscope (JIF: 2.465; n=60); The Neuroscientist (JIF: 6.5; n=4); The Oncologist (JIF: 5.025; n=1); The Physician and Sportsmedicine (JIF: 1.662; n=3); The Protein Journal (JIF: 1.317; n=1); The Spanish Journal of Psychology (JIF: 0.972; n=1); The Thoracic and Cardiovascular Surgeon (JIF: 1.209; n=10); The Turkish Journal of Gastroenterology (JIF: 1.111; n=7); The World Journal of Biological Psychiatry (JIF: 4.164; n=4); Theory & Psychology (JIF: 1.309; n=1); Therapeutic Advances in Chronic Disease (JIF: 4.257; n=7); Therapeutic Advances in Gastroenterology (JIF: 3.52; n=3); Therapeutic Advances in Hematology (JIF: 3.456; n=3); Therapeutic Advances in Medical Oncology (JIF: 6.852; n=9); Therapeutic Advances in Musculoskeletal Disease (JIF: 5.043; n=3); Therapeutic Advances in Neurological Disorders (JIF: 5; n=6); Therapeutic Advances in Psychopharmacology (JIF: 3; n=1); Therapeutic Advances in Respiratory Disease (JIF: 3.095; n=4); Therapeutic Advances in Urology (JIF: 1.918; n=6); Therapeutic Apheresis and Dialysis (JIF: 1.041; n=9); Therapeutic Innovation & Regulatory Science (JIF: 0.92; n=2); Therapeutics and Clinical Risk Management (JIF: 1.888; n=1); Thinking and Reasoning (JIF: 2.125; n=1); Thoracic Cancer (JIF: 2.61; n=16); Thrombosis and Haemostasis (JIF: 4.379; n=19); Ticks and Tick-borne Diseases (JIF: 2.749; n=1); Tissue Engineering Part C: Methods (JIF: 2.608; n=1); Toxicological and Environmental Chemistry (JIF: 1.05; n=1); Toxicology and Industrial Health (JIF: 1.708; n=3); Toxicology Mechanisms and Methods (JIF: 2.295; n=6); Traffic (JIF: 4.038; n=2); Transfusion (JIF: 2.8; n=31); Transfusion Medicine (JIF: 2.159; n=5); Translational Andrology and Urology (JIF: 2.445; n=2); Translational Cancer Research (JIF: 0.986; n=4); Translational Lung Cancer Research (JIF: 5.132; n=5); Translational Oncology (JIF: 3.558; n=2); Translational Psychiatry (JIF: 5.28; n=3); Translational Research (JIF: 5.411; n=1); Translational Vision Science & Technology (JIF: 2.112; n=1); Transplant Infectious Disease (JIF: 2.071; n=13); Transplant International (JIF: 3.177; n=28); Transplantation (JIF: 4.264; n=28); Trends in Ecology and Evolution (JIF: 14.764; n=1); Trends in Hearing (JIF: 2.244; n=1); Trends in Immunology (JIF: 13.422; n=1); Trials (JIF: 1.883; n=1); Tropical Conservation Science (JIF: 1.613; n=2); Ultraschall in der Medizin - European Journal of Ultrasound (JIF: 4.966; n=3); Ultrasonic Imaging (JIF: 1.571; n=1); Ultrasound in Obstetrics & Gynecology (JIF: 5.571; n=17); United European Gastroenterology Journal (JIF: 3.549; n=3); Urban Water Journal (JIF: 1.888; n=3); Urologic Oncology: Seminars and Original Investigations (JIF: 2.882; n=1); Vaccine (JIF: 3.143; n=2); Vadose Zone Journal (JIF: 2.504; n=1); Value in Health (JIF: 4.748; n=1); Vascular (JIF: 1.315; n=6); Vascular and Endovascular Surgery (JIF: 0.872; n=12); Vascular Medicine (JIF: 2.509; n=2); Vascular Pharmacology (JIF: 4.152; n=1); Virologica Sinica (JIF: 3.242; n=6); Visual Cognition (JIF: 1.102; n=2); Vox Sanguinis (JIF: 2.347; n=6); Waste and Biomass Valorization (JIF: 2.851; n=1); Water (JIF: 2.544; n=7); Water and Environment Journal (JIF: 1.426; n=9); Water Environment Research (JIF: 1.151; n=9); Water Resources Management (JIF: 2.924; n=2); Water Resources Research (JIF: 4.309; n=15); Water Science and Technology (JIF: 1.638; n=2); Water Supply (JIF: 0.9; n=1); Western Journal of Nursing Research (JIF: 1.602; n=4); Wildlife Society Bulletin (JIF: 1.359; n=1); Wiley Interdisciplinary Reviews: Nanomedicine and Nanobiotechnology (JIF: 7.689; n=1); Wiley Interdisciplinary Reviews: RNA (JIF: 6.913; n=1); World Journal of Clinical Cases (JIF: 1.013; n=1); World Journal of Emergency Surgery (JIF: 4.1; n=2); World Journal of Gastroenterology (JIF: 3.665; n=4); World Journal of Pediatrics (JIF: 1.437; n=1); World Journal of Surgical Oncology (JIF: 1.963; n=3); Wound Repair and Regeneration (JIF: 2.471; n=5); Xenobiotica (JIF: 1.902; n=2); Xenotransplantation (JIF: 3.484; n=5); Zeitschrift für Geburtshilfe und Neonatologie (JIF: 0.667; n=1); Zeitschrift für Orthopädie und Unfallchirurgie (JIF: 0.798; n=2); Zentralblatt für Chirurgie (JIF: 0.73; n=1)

Figure S1.1: The number of reviews by JIF group and discipline.
Note: The vertical axis shows the absolute number of reviews per JIF group. The numbers above each bar show the percentage of reviews in a JIF Group across all reviews from the discipline. The data underlying this figure can be found in S6 Data.

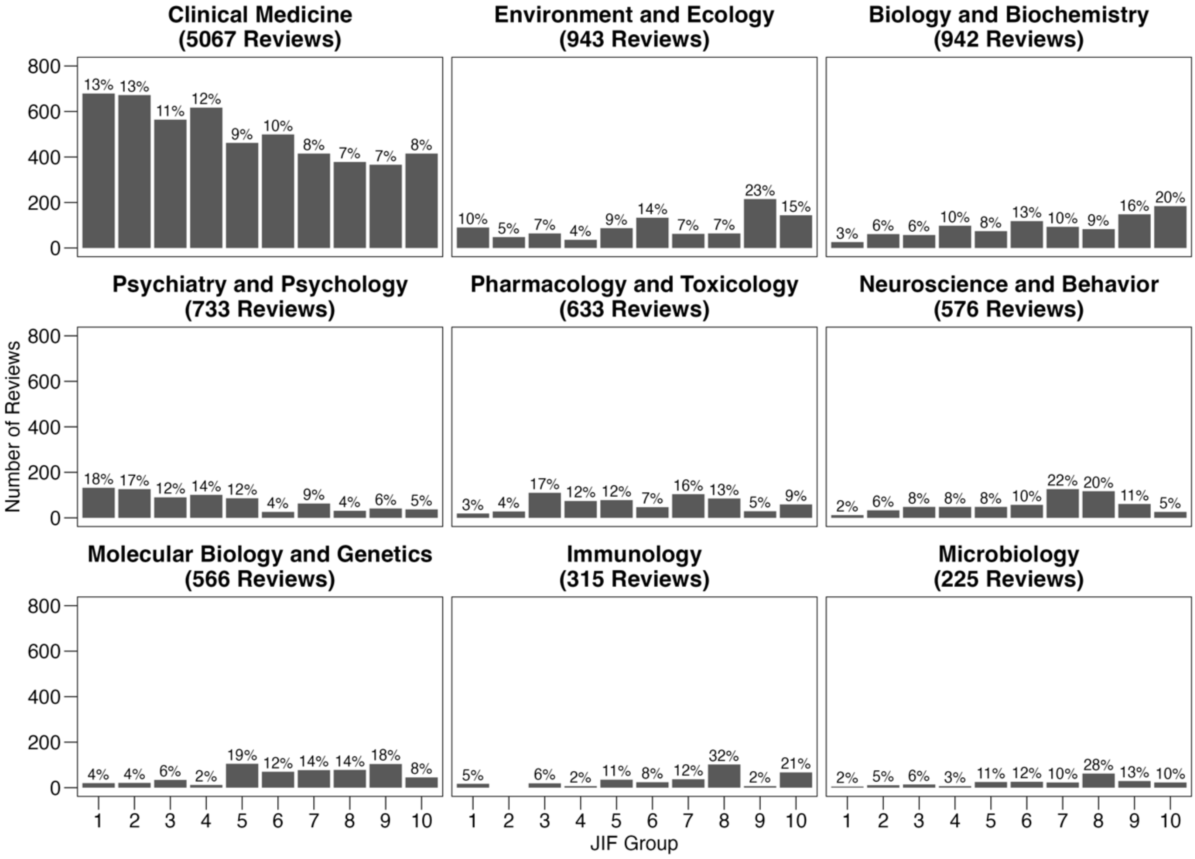

Supplement: S1 File — The 10 journals from each journal impact factor group that provided the largest number of peer review reports and all 1,664 journals included in the analysis listed in alphabetical order. The numbers in parentheses represent the JIF and the number of reviews included in the sample. (PDF) [file pbio.3002238.s001.pdf]
