# Supplementary material for: Relationship between journal impact factor and the thoroughness and helpfulness of peer reviews
Source: PLoS Biol. 2023 Aug 29;21(8):e3002238. doi: 10.1371/journal.pbio.3002238 (PMC10464996; doi:10.1371/journal.pbio.3002238)
Supplement: S4 File — Coding instructions and examples for each of the 8 characteristics of peer review reports. (PDF) [file pbio.3002238.s004.pdf]

Supporting information

Severin et al.: Relationship between Journal Impact Factor and the Thoroughness and Helpfulness of Peer Reviews

S4 File. Codebook and instructions

Below we show the coding instructions and provides examples for each of the eight characteristics of peer review reports.

All categories are to be rated on a binary scale: 1 for yes, 0 for no. One sentence can be coded 1 on multiple items.

Category: Thoroughness

Content type: Materials and Methods

- *Did the* reviewer *discuss the methods of the manuscript?*
    - This relates to whether a reviewer discusses the materials and methods used in the research. This can include strengths and weaknesses of the study design, data collection and data analysis.
    - Example: "The study design was appropriate and a good number of prison inmates (2084) were selected".
    - Do not code comments on reporting or presentation on methods under this category (e.g., "The statistical methods are incompletely described").

Content type: Presentation and Reporting

- *Did the reviewer comment on the presentation and reporting of the paper?*
    - This relates to whether a reviewer discusses presentation- and reporting- related aspects of a manuscripts: writing style, language, organization of the manuscript, tables, figures, or audio- and video files of the manuscript.
    - Example: "The manuscript is well written and readable, but some figures lack explanation"
    - Do not code general, broad statements (e.g., "The results are incompletely described").

Content type: Results and Discussion

- *Did the reviewer comment on the results and their interpretation?*
    - This relates to whether a reviewer discussed the results of a manuscript, including the results as such as well as their interpretation and discussion
    - Example: "The author's conclusions are justified by the data"
    - Do not code comments on reporting or presentation of results (e.g., "The results are incompletely described").

Content type: Importance and Relevance

- *Did the reviewer discuss the importance or relevance of the manuscript?*
  - This relates to whether the research addresses a knowledge gap in the literature, whether findings can be applied in practice or whether additional research questions should have been / could be asked.
  - Example: "Overall, this will be a useful resource for people looking to use R/Bioconductor for cytometry data analysis"

Category: Helpfulness

Content type: Suggestion and Solution

- *Did the reviewer provide suggestions for improvement or solutions?*
  - Example: "The work could benefit from more recent citations.", "The article needs language editing"

Content type: Example

- *Did the reviewer give examples to substantiate his or her comments?*
  - Example: "Some of the figure captions could be explained in more detail, for example the numbers and colouring of the entries of the heatmap in Figure 4 are presumably the median marker intensities, but this should be explicitly stated."
  - Do not code: Simple literature reference given in the reviewer comment

Content type: Praise

- *Did the reviewer identify strengths in the manuscript?*
  - Example: "The manuscript reads very well.", "The study is well-executed."

Content type: Criticism

- *Did the reviewer identify problems in the manuscript?*
  - Example: "The work lacks a qualitative approach.".
