# Supplementary material for: Relationship between journal impact factor and the thoroughness and helpfulness of peer reviews
Source: PLoS Biol. 2023 Aug 29;21(8):e3002238. doi: 10.1371/journal.pbio.3002238 (PMC10464996; doi:10.1371/journal.pbio.3002238)
Supplement: Attachment — Submitted filename: Authors response.docx [file pbio.3002238.s025.docx]

Authors’ response to reviewer comments:
Severin et al. Journal Impact Factor and Peer Review Thoroughness and Helpfulness: A Supervised Machine Learning Study

We are grateful to the reviewers for their constructive comments which have greatly helped improve our paper. Below we address these comments and document the changes made to the manuscript.

# Reviewer #1:

[identifies himself as Ludo Waltman]

Please find my review at <https://ludowaltman.pubpub.org/pub/review-jif-pr/release/1>

[the editor has here pasted the text of the review from that location]

This paper presents a large-scale analysis of the content of peer review reports, focusing on different types of comments provided in review reports and the association with the impact factors of journals. The scale of the analysis is impressive. Studies of the content of such a large number of review reports are exceptional. I enjoyed reading the paper, even though I did not find the results presented in the paper to be particularly surprising.

Feedback and suggestions for improvements are provided below.

1. The methods used by the authors would benefit from a significantly more detailed explanation:

“Scholars can submit their reviews for other journals by either forwarding the review confirmation emails from the journals to Publons or by sending a screenshot of the review from the peer review submission system.”: This sentence is unclear. Review confirmation emails often do not include the review itself, only a brief ‘thank you’ message, so it is not clear to me how a review can be obtained from such a confirmation email. I also do not understand how a review can be obtained from a screenshot. A screenshot may show only part of the review, not the entire review, and there would be a significant technical challenge in converting the screenshot, which is an image, to machine-readable text.

Response: Thank you. We acknowledge that this was not very clear. The sentence describes how reviews may be verified on Publons. However, the actual content will either have been uploaded by the reviewer or have been received from journals that partner with Publons after a reviewer has opted in to get credit on Publons and include the content of the review. We have now clarified this in the revised version of the paper:

*Reviews can be submitted to Publons in different ways. When scholars review for journals partnering with Publons and wish recognition, Publons receives the review and some meta-data directly from the journal. For other journals, scholars can upload the review and verify it by forwarding the confirmation email from the journal to Publons or by sending a screenshot from the peer review submission system.*

2. I would like to know whether all reviews are in English or whether there are also reviews in other languages.

Response: We thank the reviewer for this question. Reviews in all languages can be submitted to Publons. For our study, we excluded reviews in other languages than English as the algorithm was trained using English language reviews. We added this information to the Methods section:

*Publons randomly selected English-language peer review reports for the training from a broad spectrum of journals covering all Clarivate's Essential Science Indicator (ESI) fields [37] except Physics, Space Science and Mathematics. […] In the next step, a stratified random sample of 10,000 verified pre-publication reviews written in English was drawn.*

3. Impact factors change over time. New impact factors are calculated each year. The authors need to explain which impact factors they used. There are many journals that do not have an impact factor. The authors need to explain how these journals were handled.

Response: Thank you. The 2019 impact factors were obtained from the Journal Citation Reports published by Clarivate. Reviews for journals without impact factor were excluded from the analyses. We clarified this in the Methods section as follows:

*After validating the classification performance using out-of-sample predictions, the association between the 2019 journal impact factors* [36] *and the prevalence of relevant sentences in peer review was examined.*

4. The authors also need to discuss how reviewers were linked to publication profiles. This is a non-trivial step that needs to be taken to determine the number of publications of a reviewer and the start and end year of the publications of a reviewer. The authors do not explain how this step was taken in their analysis. It is important to provide this information.

Response: Publication profiles of reviewers were based on the information provided by reviewers themselves for their Publons profiles. Publons allow users to claim authorships of their publications using various sources, including Web of Science. The completeness and accuracy of publication profiles is therefore reliant on users’ input.

5. “We used a Naïve Bayes algorithm to train the classifier and predict the absence or presence of the eight characteristics in each sentence of the peer review report.”: The machine learning approach used by the authors is explained in just one sentence. A more elaborate explanation is needed. There are lots of machine learning approaches. The authors need to explain why they use Naïve Bayes. They also need to briefly discuss how Naïve Bayes performs the classification task.

Response: We thank the reviewer for this comment. We now specify in detail which classifiers and specifications we used. As mentioned in our response to Reviewer 3 below, for the revised version of the paper, we used the transformer-based model DistilBERT to classify sentences for the eight content categories. We chose a transformer-based classifier for the revision of this paper since these models outperform other machine learning models (see, for example, Minaee S et al. ACM Comput Surv. 2022 Apr;54(3):62). This approach is computationally more demanding but it greatly improved the classification performance. See the Figure included in the response to Reviewer 3 comparing the F1 scores of the DistilBERT classifier with the Naive Bayes approach. Of note, the substantive results are very similar despite the much better F1 scores for DistilBERT. In other words, while we improved the classification performance, the conclusions remain the same. Table 2 now lists the 50 most “predictive” terms for each content category (based on a keyness analysis which we explain in Section Typical words in content categories). This analysis provides further support for the face validity of our new approach. The new section on Classification and validation now reads as follows:

*We used the transformer based-classifier DistilBERT to predict the absence or presence of the eight characteristics in each sentence of the peer review reports [45]. For validation, data were split randomly into a training set of 1,600 sentences and an held-out test set of 400 sentences. Eight DistilBERT models (one for each content categories) were fine-tuned on the set of 1,600 sentences and predicted the categories in the remaining 400 sentences. Performance measures, including precision (i.e. the positive predictive value), recall (i.e. sensitivity), and the F1 score were calculated. The F1 score is a harmonic mean of precision and recall and an overall measure of accuracy. The F1 score can range between 0 and 1, with higher values indicating better classification performance [46].*

*Overall, the classification performance of the DistilBERT approach was high. The average F1 score for the presence of a characteristic was 0.75, ranging from 0.68 (Praise) to 0.88 (Suggestion and Solution). The average F1 score for the absence of a characterstic was 0.93, ranging from 0.83 (Materials and Methods) to 0.97 (Praise, Importance and Relevance). For most categories, precision and recall were similar, indicating the absence of systematic measurement error. Importance and Relevance and Results and Discussion were the exceptions, with lower recall for characteristics being present. Balanced accuracy (the arithmetic mean of sensitivity and specificity) was also high, ranging from 0.78 to 0.91 (with a mean of 0.83 across the eight categories). S2 File gives further details.*

*We compared the percentages of sentences addressing each category between the human annotation dataset and the output from the machine learning model. For the test set of 400 sentences, the percentage of sentences that fall into each of the eight categories were calculated, separately for the human codings and the DistilBERT predictions. There was a close match between the two: DistilBERT overestimated Importance and Relevance by 3.0 percentage points and underestimated Materials and Methods by 2.3 percentage points). For all other content categories, smaller differences were observed. Having assessed the validity of the classification, the DistilBERT classifiers were fine-tuned using all 2,000 labelled sentences, and the eight classifiers were used to predict the presence or absence of content in the full text corpus consisting of 188,106 sentences.*

6. Likewise, I would like to see a proper discussion of the statistical model used by the authors. The authors informally explain their statistical approach. I would find it helpful to see a more formal description (in mathematical notation) of the statistical model used by the authors.

Response: The revised version includes a more detailed description of the statistical approach and mathematical notation. The revised manuscript now reads:

*The association between peer review characteristics and journal impact factor groups was examined using a series of linear mixed-effects models to account for the clustered and hierarchical nature of the data [48]. The main regression model takes the form,*

$$Y_{i}=\alpha_{j\left[ i \right],k\left[ i \right]}+\sum_{m=2}^{10} \beta_{m}\mathbb{\cdot I}\left( JIF_{i}=m \right)+\gamma\cdot Length_{i}+\epsilon_{i}$$

*with*

$\alpha_{j}\sim N\left( \mu_{\alpha_{j}},\sigma_{\alpha_{j}}^{2} \right)$*, for journal* $j=1,\ldots,J$

$\alpha_{k}\sim N\left( \mu_{\alpha_{k}},\sigma_{\alpha_{k}}^{2} \right)$*, for reviewer* $k=1,\ldots,K$

$$\epsilon_{i}\sim N\left( 0,\sigma^{2} \right)$$

*where* $Y_{i}$ *is the proportion of a content category in a review report* $i$*,* $\beta_{m}$ *are the coefficients for the* $m=2,\ldots,10$ *categories of the categorical variable of Journal Impact Factor (with* $m=1$ *as the reference category),* $\gamma$ *is the coefficient for review length and* $\epsilon_{i}$ *is the unobserved error term. The model includes varying intercepts* $\alpha_{j\left[ i \right],k\left[ i \right]}$ *for* $J$ *journals and* $K$ *reviewers.* $\mathbb{I}\left( \cdot\right)$ *denotes the indicator function.*

*All regression analyses were done in R (version 4.2.1). The fine-tuning of the classifier and sentence-level predictions were done in Python (version 3.8.13). The libraries used for data preparation, text analysis, supervised classification, and regression models, were transformers (version 4.20.1) [49], quanteda (version 3.2.3) and quanteda.textstats (version 0.95) [50], lme4 (version 1.1.30) [51], tidyverse (version 1.3.2) [52], and texreg (version 1.38.6) [53].*

7. “Most distributions were skewed right, with a peak at 0% showing the number of reviews that did not address the content category (Fig 1).”: I do not understand how the peaks at 0% can be explained. Could this be due to problems in the data (e.g., missing or empty review reports)? The authors need to explain this.

Response: There were no missing or empty review reports. However, some of the reviews were very brief. For this reason, many reviews did not address some of the categories, resulting in the spikes at 0%. Since review length could influence our findings, we control for the number of words in all regression models. Further, we performed we performed two sensitivity analyses that are relevant to this issue. We mention these in the main text as follows and provide further details in the S3 File:

*We performed several sensitivity analyses to assess the robustness of findings. In the first, we removed reviews with a prevalence of 0% in the respective content category, resulting in similar regression coefficients. In the second, the sample was limited to reviews longer than 200 words. The median review length increased from 282 (IQR 138–501) to 436 (IQR 298–667) words. Again, the analysis showed that short reviews do not drive associations. In a third sensitivity analysis, the regression models adjusted for additional variables (discipline, career stage of reviewers, and logged number of reviews submitted). The addition of these variables strengthened relationships between content categories and journal impact factor. The fourth sensitivity analysis revealed that results were generally similar for male and female reviewers. The fifth showed that the results change little when replacing the journal impact factor groups with the raw journal impact factor (S3 File).*

8. “the prevalence of content related to thoroughness and helpfulness varied widely even between journals with similar journal impact factor”: I am not sure whether the word ‘between’ is correct in this sentence. My understanding is that the authors did not distinguish between variation between journals and variation within journals.

Response: Thank you for this comment. This statement in the Discussion refers to Figure 2, which shows the estimated prevalence of sentences addressing the eight content categories across the ten journal impact factor groups. It is clear from this graph that within the ten groups with similar impact factors, there is a wide variation in coverage of the different content categories. This variation will most likely to be due to both between and within journal variation: each group included about 100 to 250 journals and 1000 peer review reports. We have revised the sentence to read:

*Similar to the distribution of citations in a journal, the prevalence of content related to thoroughness and helpfulness varied widely, within journals and between journals with similar journal impact factor. In other words, the journal impact factor is a poor proxy measure for the thoroughness or helpfulness of peer review authors may expect when submitting their manuscripts.*

“Some journals now publish peer reviews and authors' responses with the articles”: Consider citing the following paper: <https://doi.org/10.1007/s11192-020-03488-4>. I also recently published a blog post on this topic: <https://www.leidenmadtrics.nl/articles/the-growth-of-open-peer-review>.

9. “Bibliographic databases have also started to publish reviews.”: In addition to Web of Science, I think the work done by Europe PMC needs to be acknowledged as well. See for instance this poster presented at the recent OASPA conference: <https://oaspa.org/wp-content/uploads/2022/09/Melissa-Harrison_COASP-2022-poster_V2.pdf>.

Response: We thank the reviewer for the literature suggestions. We now additionally cite the excellent article by Wolfram et al. on open peer review., We do not mention Europe PMC because, to the best of our knowledge, it does not publish peer review reports (see Ferguson et al., PMID: 33180112).

10. “peer review in journals with higher impact factors tends to be more thorough in addressing study methods but less helpful in suggesting solutions or providing examples”: I wonder whether this conclusion is justified. Relatively speaking sentences in reviews for higher impact factor journals are indeed more likely to address methods and less likely to suggest solutions or to provide examples. However, as shown by the authors, reviews for higher impact factor journals tend to be substantially longer than reviews for lower impact factor journals. Therefore it seems that the total number of sentences (as opposed to the proportion of sentences) suggesting solutions or providing examples may be higher in reviews for higher impact factor journals than in reviews for lower impact factor journals. If that is indeed the case, it seems to me the conclusion should be that peer review in higher impact factor journals is both more thorough and more helpful.

Response: We believe the statement is justified. Please note that our analyses controlled for the length of peer review controls. Also, they were robust to excluding very short reports (200 words or shorter – see above).

11. Finally, I think it needs to be acknowledged that quality assurance processes of journals consist not only of the work done by peer reviewers but also of the work done the editorial staff of journals. This seems important in particular for more prestigious journals, which presumably make more significant investments in editorial quality assurance processes. The results presented in the paper offer valuable insights into peer review processes, but they provide only a partial picture of the overall quality assurance processes of journals.

Response. Thank you, we very much agree with this comment and now make this point in the Discussion section of the revised paper:

*We only examined peer review reports and could not consider the often extensive contributions made by journal editors and editorial staff to improve articles. In other words, although our results provide valuable insights into the peer review process, they give an incomplete picture of the general quality assurance processes of journals.*

# Reviewer #2:

[identifies himself as Bernd Pulverer]

Severin et al. add to their previous work (ref 23) on analyzing attributes of scholarly referee reports. Peer review is generally regarded as a pivotal component of the scholarly process and as such quantitative analysis is to be welcomed.

The study is based on a large set of about 10,000 referee report across a broad set if biomedical disciplines and uses human annotation to train machine learning based extraction according to 8 pre-identified categories. The study limits itself to analyzing how the 8 referee report attributes compare across 10 Journal Impact Factor (JIF) bins. Regression modelling is applied. Several of the attributes exhibit no trends, others at best very weak trends. That in itself is notable, as for example the minor negative trend of comments on 'Importance and Relevance' vs. JIF is surprising as higher JIF journals tend to instruct referees to comment specifically on these attributes, which for a core part of the selection criteria of such journals. Stronger correlations are reported for the categories 'Materials and Methods' (positive), 'Presentation and Reporting' and 'Suggestion and Solution' (both negative). The authors conclude that referee reports for higher JIF journals may be more 'thorough' but less 'helpful in suggesting solutions and providing examples'. These trends are notable and not predictable - they are also somewhat difficult to rationalize and it is to the authors credit that they don't overinterpret these numbers beyond the conclusions that 'JIF is a bad predictor of peer review' and in fact end the paper with a balanced strength/weakness analysis.

Response: We thank the reviewer for the positive assessment of our paper.

The data and approach as reported in detail, but source data for fig 1-3 should be added.

Response: All analyses are based on our set of 10,000 reviews. We clarify the data source in Figures 1–3 and specify the unit of analysis (sentences vs reviews).

This is an important area of analysis of general interest and the study is thorough. The conclusions are somewhat limited by restricting the analysis to one variable, the JIF, and by limiting the referee report attributes to 8 categories (see below). With the heavy lifting of the human curated training set in hand, it is a pity that the study was not developed beyond the JIF correlations. As such, this specific analysis appears novel and it is based on a large dataset, albeit form a single source.

Major comments:

1)	The dataset is large, but limited to one database (Publons). This may well add biases the data, as the authors note themselves. It would have been helpful to expand the analysis to other databases hosting referee reports, such as ORCID, as well as to journals that publish referee reports alongside their papers, such as BMJ, EMBO, eLife and some Nature branded journals. Minimally to test if the reported trends hold up.

Response: We thank the reviewer for this suggestion. To the best of our knowledge, ORCID does not include the actual peer review reports but provides only a record of the review activity. Publons was identified as the most suitable database for the study as it covers a wide range of different publishers and journals while allowing to extract reviews and meta-data systematically. Many of the mentioned publishers and journals partner with Publons and have many thousands of reviews on Publons (e.g. BMJ, BMJ Open, eLife, The EMBO Journal etc). While using Publons as the database for this study reduces certain biases related to restricting the analyses to individual publishers or journals, we acknowledge that it might introduce other biases to the data, e.g. self-selection biases in submitting reviews to Publons. We examine these biases in the Discussion section.

2)	It would also have been useful to test for another potential bias: open reports vs. closed reports (still the majority): a collaboration with journals that do not publish their reports (and filtering out referees who posted on Publons or ORCID) would have led to an interesting comparison if the trends are identical when peer review is confidential. Since only aggregate data are reported a journal/publisher collaboration should be feasible.

Response: Thank you. We agree that it would have been interesting to explore differences between open and closed peer review reports. Due to limitations in the available data, we were not able to explore this question. We now included this limitation in the Discussion section as follows:

*We could not examine any differences between open (signed) and anonymous peer review reports. Similarly, we could not distinguish between reviews of original research articles and other article types, for example, narrative review articles.*

3)	The study is based on 8 categories. It is unclear how these categories were chosen and the detail of how the annotators defined them is limited. More importantly, other important attributes are missing, for example number of experimental requests made vs. number of textual requests made, or % of a referee report dedicated to specific points vs. general discussion/subjective points. Expanding the set would add value. As a minor point, it is noted that one category, 'Importance and Relevance' is explicitly excluded from a number of major journals, such as PLOSONE. This could be a confounding factor. I realize that 'multidisciplinary' journals have already been excluded, and maybe this covers all such journals, but please comment.

Response: We agree with the reviewer that there may be additional attributes of interest. The eight categories used in this study build on the Review Quality Instrument (RQI) by van Rooyen, Black & Godlee (1999) and other previous work ( e.g. Superchi et al. 2019). We refined the categories in a step-by-step manner in the manual coding process taking into account the ease of operationalizing categories and their intercoder reliability. We now describe the process in greater detail as follows:

*Two authors (ASE, MS) were trained in coding sentences. After piloting and refining coding and establishing intercoder reliability, the reviewers labelled 2,000 sentences (1,000 sentences each). They allocated sentences to none, one, or several of eight content categories. We selected the eight categories based on prior work, including the Review Quality Instrument and other scales and checklists [38], and previous studies using text analysis or machine learning to assess student and peer review reports [39–43]. In the manual coding process, the categories were refined, taking into account the ease of operationalizing categories and their intercoder reliability. Based on the pilot data, Krippendorff's α, a measure of reliability in content analysis, was calculated [44].*

We also agree that it would be desirable to identify all journals that explicitly exclude the category ‘Importance and Relevance’ from the assessment criteria. Unfortunately, it was not feasible to examine this issue. The Publons database does not currently include the article type, however, most articles will have been original contributions. We now acknowledge this limitation in the Discussion section as follows:

*Some journals do not consider importance and relevance when assessing submissions, and these journals may have influenced results for this category. We lacked the resources to identify these journals among the over 1600 outlets included in our study to examine their influence.*

4)	It is unclear if only research papers were analyzed. It is recommended that other peer reviewed papers such as reviews are excluded.

Response: Thank you. Again, due to the limitations of the Publons database, we could not assess the type of article reviewed, but most reviews will have been of original articles. We now acknowledge this limitation in the Discussion section of the revised paper (see response to your point 2 above).

5)	The study shows that the JIF does not predict many of the attributes. With the same dataset other variables could be assessed, such as 'subject area' (already defined in the study as ESI research field, in particular clinical research vs. 'basic research'). This is in particular important as baseline JIF is rather different between such categories, which may be a confounding factor in this analysis, but it may also lead to stronger correlations than JIF. Other variables could be category of paper (short report vs. full research paper) or length of paper (report correlation between paper and referee report length). Other interesting areas would be journal name, journal editorial process, referee age or experience, referee gender, referee affiliation. The authors note that referee age could not be analyzed and discuss other variables noting 'adjusting for additional variables strengthened relationships'. It is recommended that this section is expanded and the data added.

Reponse: We agree with the reviewer that it would have been interesting to address additional questions and analyze further variables. However, the paper is already quite dense and some of the reviewer’s suggestions cannot be implemented with the current dataset, for example regarding the type of paper.

Please note that we controlled for the ESI disciplines in our third sensitivity analysis and obtained similar results (see response to question 8 of Reviewer #1 above). In the Discussion we added a sentence making the point that future work should investigate differences between disciplines.

The referee geography as a function of JIF is reported in Table 1: it would be interesting to correlate this with that of the corresponding authors of the paper refereed, if that is feasible.

Response: We thank the reviewer for this interesting suggestion, but again this was not the focus of our paper. We may look into this in future analyses.

6)	The 'trends' seen for 'Importance and Relevance' and 'Example' (fig 3) are

reported as statistically significant, but they are very small and arguable hard to interpret on the background of complex confounding factors. 'Criticism' shows arguable a similar range of variation and yet is classed as 'no effect'. I would recommend not to emphasize these.

Response: Thank you. We agree with this assessment and have rephrased this section accordingly.

Minor Comments:

1)	The very first sentence of the abstract states that JIF is used as a proxy for journal quality and thus peer review quality. First of all, JIF claims to measure 'impact' not quality and this is a distortion, although both may correlate. Also, as stated it is implied that referees select what is published, which is not the case (editors select assisted by referee input). Thus, even assuming editors select for JIF maximization, the JIF to peer review connection is indirect at best.

Response: Thank you. Yes, we meant to say that the JIF is used as a proxy measure for journal quality. We did not mean to imply that the referees (rather than the editors) select the papers to be published. We have rephrased this section as follows:

*The* impact factor *was originally developed to help libraries make indexing and purchasing decisions for their collections. It is a journal-based metric calculated each year by dividing the number of citations received in that year for papers published in the two preceding years by the number of “citable items” published during the two preceding years [7]. The reputation of a journal, its impact factor, and the perceived quality of peer review are among the most common criteria authors use to select journals to publish their work [8–10]. Assuming that citation frequency reflects a journal’s importance in the field, the impact factor is often used as a proxy for journal quality [11]. It is also used in academic promotion, hiring decisions, and research funding allocation, leading scholars to seek publication in journals with high impact factors [12].*

2)	Please explain why the regression analyses were controlled for review length. 'since longer texts …address more categories' seems tenuous as multiple categories can be assigned to each sentence.

Response: Shorter reviews will tend to have a lower probability of addressing a given category. This is why we controlled for the review length. Please note that we present both crude and adjusted estimates. As expected, when removing the length of reviews, the relationships for our main categories of interest become slightly stronger.

3)	Discussion, second paragraph: a key outcome of studies such as this is to develop 'referee credit' systems. Processes such as the referee report analysis applied here can be applied to individual referee reports and referees to aid such a system.

Response: We agree and make this point in the Discussion. Please note that Publons is indeed a ‘referee credit’ system.

4)	The 'Typical words' section could be removed as it is covered in S4.

Response: We have expanded this section in the text (and removed it from S4 File), presenting typical words, based on our revised keyness analysis of the entire text corpus. We believe this additional analysis strengthens the validity of the classification and informs readers about which words appear very frequently in a certain content category, relative to sentences not classified into the category. See the new Table 2 in the main paper and the response to point 4 of reviewer 3.

5)	Table 2 is of limited value and could be removed or added as a supplementary figure.

Response: We agree and moved it to the online supplement.

Text suggestions:

1)	Abstract , line 3: add also no. papers analyzed here.

2)	Abstract, line 9: state whole range to avoid confusion: 0.21-74.70, median 1.2-8.0

3)	Introduction, lines 4-9: I suggest to remove the claim that peer review is 'particularly critical for the medical sciences'. This is debatable, but the paper is not restricted to the medical sciences (in fact, as noted above, a comparison between medical and biological sciences in this dataset would add considerably).

Response: We have followed all of the reviewer’s suggestions. We rephrased the sentence referring to the medical sciences as follows:

*In medicine, practitioners rely on sound evidence from clinical research to make a diagnosis or prognosis and choose a therapy.*

4)	Introduction, second para: the claim 'in the absence of evidence on the quality of peer review ….proxy measures like JIF…' is tenuous at best. JIF is used as a proxy for 'impact' maybe even 'quality' and peer review is a key part of quality assurance but does not in itself define journal selection. In fact, one could highlight two functions: aiding journal selection; improving paper. Please adapt.

Response: Thank you. We have rephrased this paragraph as follows and cite additional literature:

*The impact factor was originally developed to help libraries make indexing and purchasing decisions for their collections. It is a journal-based metric calculated each year by dividing the number of citations received in that year for papers published in the two preceding years by the number of “citable items” published during the two preceding years [7]. The reputation of a journal, its impact factor, and the perceived quality of peer review are among the most common criteria authors use to select journals to publish their work [8–10]. Assuming that citation frequency reflects a journal’s importance in the field, the impact factor is often used as a proxy for journal quality [11]. It is also used in academic promotion, hiring decisions, and research funding allocation, leading scholars to seek publication in journals with high impact factors [12].*

*Despite using the journal impact factor as a proxy for a journal’s quality, empirical research on the impact factor as a measure of journal quality is scarce [11]. In particular, it is unclear how the peer review characteristics for a journal relate to this metric. We combined human coding of peer review reports and quantitative text analysis to examine the association between peer review characteristics and journal impact factor in the medical and life sciences, based on a sample of 10,000 peer review reports.*

5)	Introduction, second para: change 'articles published' to 'articles classed as citable (by ISI-Clarivate)'

Response: Done, many thanks. See above.

6)	Discussion, line 14: I am not sure the data definitively show high JIF reports are 'more thorough'.

Response: We say that they may be more thorough than those submitted to lower impact journals. We believe this statement is in order based on our results.

7)	Discussion, 3rd para: I disagree with the hypothesis that 'junior referees might be less able to comment on methodology'. All the evidence point the other way, and this is not surprising since ECRs are practitioners. It is fine to pose a hypothesis of course and then to cite evidence again, but this section could also be deleted as it is - unfortunately - not tested here.

Response: We agree with the reviewer and have shortened this section, removing the sentence on the junior referees. It now reads as follows:

*The increase in the length of peer review reports with increasing journal impact factor might be explained by the fact that reviewers from Europe and North America and reviewers with English as their first language tend to write longer reports, and to review for higher impact journals [20]. Further, high impact factor journals may be more prestigious to review for and can thus afford to recruit more senior scholars. Of note, there is evidence suggesting that the quality of reports decreases with age or years of reviewing [21,22].*

8)	Discussion 4th para: Ref 19, 20 are cited in support of transparent/open peer review. Nature was actually rather late in adopting this and others, like BMJ group, EmboPress, BMC series and eLife, could be cited.

Response: We agree and now cite other publishers as well.

9)	Discussion 5th para: ORCID should be discussed here

Response: ORCID does not provide the actual peer review reports. It partners with databases such as Publons, which host the reports. Among those databases Publons is - to the best of our knowledge - the largest one, which is not restricted to individual publishers/ journals.

10)	Methods: Publons has been part of Clarivate for years not 'now'.

Response: Revised to “Publons (part of Web of Science)”, thank you.

This referee I not an expert in machine learning or statistical analysis and did therefore not assess these aspects of the work in detail.

# Reviewer #3:

While this is a highly interesting study, there are several major questions and issues that preclude a favorable assessment at this point.

1) Introduction

It´s relatively well known, at least in my circles, that the impact factor (IF) is misused to assess journal quality and even single paper quality. However, I do not necessarily agree with the notion that this included an overestimation of peer review quality as well. Curiously, the manuscript also does not provide a single reference to back this extension ("re used to assess the quality of journals and, by extension, the quality of peer review."). This is problematic since the premise of the introduction lies upon this idea.

The authors should provide evidence for the notion that IF and peer review quality are linked or have been perceived as linked.

Response: We thank the reviewer for this comment. We have rephrased this paragraph - see our response to Reviewer #2, point 4, above.

2) Methods

General comment on the methods: The machine learning pipeline is not very well described, also with the added supplement. A supplement should contain no information that is absolutely necessary to understand the methodology.

I strongly suggest a general revision for clarity using standard machine learning terminology and phrasings, and review what is in the main manuscript and the supplement.

One example:

"We divided the sample into five equally sized subsets and ran the cross-validation five times."

This is how cross-validation was explained. While this explanation _could_ in theory mean cross-validation, this definition could also support other data splits which are not cross-validation.

Response: We are grateful for this comment. It has motivated us to move to a more advanced and reliable classification approach. Instead of Naive Bayes or other bag-of-words classifiers, we now conduct our analysis using DistilBERT (Sanh et al. 2019, cited in revised manuscript), a transformer-based classifier. DistilBERT is a more efficient version of BERT, which at the time was considered a major breakthrough by the Machine Learning community. Generally speaking, transformer models are a type of neural network specifically designed to work with natural language data. They use a technique called self-attention to process the input text, which allows them to consider the context of the words in the input when generating output. This makes transformer models well-suited for tasks like text classification because they can effectively capture the relationships between words in the input (Tunstall et al. 2022, cited in revised manuscript).

Transformers have been shown to outperform previous approaches for text classification. Moreover, a relatively small training set is often sufficient for fine-tuning the transformer for a specific task (in our case: identifying characteristics of peer review reports). Of course classic machine classifiers also have advantages. They are often much less computationally demanding, take less time to run, and the classification process is more interpretable (for a discussion of this trade-off see, Ash and Hansen, Forthcoming in Annual Review of Economics, available from [Text Algorithms in Economics∗ - Research Collection (ethz.ch)](https://www.research-collection.ethz.ch/handle/20.500.11850/593179)). However, we believe transformer models are the superior choice for this paper. DistilBERT vastly outperforms the bag-of-words approach presented in the previous version of the manuscript and provides a very good classification performance. It is important to keep in mind that our classification task is difficult: we try to uncover often latent and ambiguous characteristics of texts written by academics. To put our overall classification performance into perspective: Bonikowski et al (2022, cited in revised manuscript) recently used RoBERTa, an extension of BERT, to classify concepts such as nationalism, authoritarianism, and trust. Precision ranges from 0.63 to 0.85; recall ranges from 0.55 to 0.78. Precision and recall for Populism are 0.66 and 0.68, respectively. The performance of our classifiers is comparable or better.

We do not claim to have identified an ideal solution and best classifier for identifying peer review characteristics. Instead, we use transfer learning to scale up human coding while trying to reduce systematic measurement error. The classifiers then allow us to address a substantively important research question. We hope that our revisions and extensions have achieved this goal. See the revised manuscript and our response to point 5 of Reviewer 1 above for the new section on Classification and validation.

3) Methods / Classification and Validation

In the section that describes the categories, I noted that some points were labeled as "did the reviewers _discuss_" a certain topic, whereas in others the label was "did the reviewers _comment on_" a topic.

To comment on something or to discuss something is a clear qualitative difference. Is there a reason why these phrasings were used?

Response: Thank you. We have harmonized the descriptions – we did not make a difference between ‘comment’ and ‘discuss’.

4) Methods / metrics

The method sections reads as if the authors only calculated PPV, sensitivity, and the F1 score. However, the supplement S2 also describes and shows the accuracy. I assume that the authors calculated an even bigger set of metrics. So please justify why these particular 3 (or 4) metrics were chosen to be presented in the paper (and why others were not).

Response: We thank the reviewer for this suggestion. Precision, Recall, and the F1 score are among the most commonly used metrics and allow for a straightforward comparison of our measures with existing work. In the revised version, we report Precision, Recall, and the F1 score as our main measures. An advantage of the F1 score is that it considers class imbalances. In the revised validation analysis, we report all scores for both classes (characteristic absent vs characteristic present) for each category, rather than using the average. The new paragraphs now read as follows:

*We used the transformer based-classifier DistilBERT to predict the absence or presence of the eight characteristics in each sentence of the peer review reports [45]. For validation, data were split randomly into a training set of 1,600 sentences and an held-out test set of 400 sentences. Eight DistilBERT models (one for each content categories) were fine-tuned on the set of 1,600 sentences and predicted the categories in the remaining 400 sentences. Performance measures, including precision (i.e. the positive predictive value), recall (i.e. sensitivity), and the F1 score were calculated. The F1 score is a harmonic mean of precision and recall and an overall measure of accuracy. The F1 score can range between 0 and 1, with higher values indicating better classification performance [46].*

*Overall, the classification performance of the DistilBERT approach was high. The average F1 score for the presence of a characteristic was 0.75, ranging from 0.68 (Praise) to 0.88 (Suggestion and Solution). The average F1 score for the absence of a characterstic was 0.93, ranging from 0.83 (Materials and Methods) to 0.97 (Praise, Importance and Relevance). For most categories, precision and recall were similar, indicating the absence of systematic measurement error. Importance and Relevance and Results and Discussion were the exceptions, with lower recall for characteristics being present. Balanced accuracy (the arithmetic mean of sensitivity and specificity) was also high, ranging from 0.78 to 0.91 (with a mean of 0.83 across the eight categories). S2 File gives further details.*

*We compared the percentages of sentences addressing each category between the human annotation dataset and the output from the machine learning model. For the test set of 400 sentences, the percentage of sentences that fall into each of the eight categories were calculated, separately for the human codings and the DistilBERT predictions. There was a close match between the two: DistilBERT overestimated Importance and Relevance by 3.0 percentage points and underestimated Materials and Methods by 2.3 percentage points). For all other content categories, smaller differences were observed. Having assessed the validity of the classification, the DistilBERT classifiers were fine-tuned using all 2,000 labelled sentences, and the eight classifiers were used to predict the presence or absence of content in the full text corpus consisting of 188,106 sentences.*

We also extended our keyness analysis. Instead of analysing terms in the annotated set, we expand it to the full corpus of 188,106 sentences. For each content category, we report the 100 features (words, numbers) with the highest chi2 values. These terms appear much more often in sentences classified into the respective category, relative to the sentences not mentioning this content category (according to the DistilBERT classifier). The extended keyness analysis provides further validity for our results and exemplifies which terms are “typical” for each content category. The new paragraph now reads:

*A keyness analysis [15] extracts typical words for each content category across the full corpus of the 188,106 sentences. The analysis is based on χ2 tests comparing the frequencies of each word in sentences assigned to a content category and other sentences. Table 2 reports the 50 words appearing more frequently in sentences assigned to the respective content category than in other sentences (according to the DistilBERT classification). The table supports the validity of the classification. Common terms in the thoroughness categories were 'data', 'analysis', 'method' (Materials and Methods); 'please', 'text', 'sentence', 'line, figure' (Presentation and Reporting); 'results', 'discussion', 'findings' (Results and Discussion); and 'interesting', 'important', 'topic' (Importance and Relevance). For helpfulness, common unique words included 'please', 'need', 'include' (Suggestion and Solution); 'line', 'page', 'figure' (Examples); 'interesting', 'good', 'well' (Praise); and 'however', '(un)clear', 'mistakes' (Criticism).*

See also Table 2 in the revised manuscript.

5) Methods / Results

Generally, the performance of the models is pretty bad. Even the top three that were chosen for further analysis perform pretty badly. Also, the authors have compared only NB and an SVM. If this was a data science project in a bootcamp, the authors would fail it as they kind of stopped after 40% of the work. Especially boosted trees would have been worth exploring as they consistently rank highest in the literature and in competition compared to simpler algorithms. Together with point 6 (below) and proper hyperparameter tuning it is likely that a boosted tree model would lead to better results.

Response: Thank you. Our entirely new classification (see explanation above) vastly improved the performance of our models, which do not seem to suffer from substantial systematic classification errors. As mentioned above, DistilBERT is computationally more demanding than bag-of-words classifiers but results in much better performance. However, the substantive conclusions are the same. We detect differences for *Materials and Methods*, *Presentation and Reporting*, and *Suggestion and Solution* based on the Naive Bayes approach and the DistilBERT classification. In other words, our substantive contribution to the literature does not seem to depend on a single classifier.

The Figure below shows the substantial improvement of the performance with DistilBERT compared to Naïve Bayes.

6) Methods

Looking at S2, it becomes pretty clear that there is an imbalance problem, definitely present for the 5 least common categories. I did not find any mention that the authors adjusted their k-fold crossvalidation for imbalanced data. In this case stratified sampling for the k-fold cross-validation is the right method which would likely lead to better and more stable results. To assess this the authors should also report the standard deviation of the cross-validation. I would generally also suggest 10 instead of 5 folds when dealing with such a more complicated setup.

Response: Thank you. Our revised approach does not allow for estimating the standard deviation of the cross-validation since we decided to compare our classifiers across the same test and training set. For transformer-based classifiers, k-fold cross-validation is not widely used since the fine-tuning of the transformers for each fold can take several hours and because the performance is often superior to other approaches. For example, the recent textbook on transformers for classification does not even mention k-fold cross-validation (Tunstall et al. 2022). However, we report a wide set of performance metrics that account for the class imbalance, and report metrics separately for both classes (characteristic absent and present) rather than reporting the binary average across the two classes. See also S2 File.

7) Results

How the paper is written, it is very suggestive that the authors believe that the correlation found between high-impact journals and peer-review focusing more on methods is also causal. While the authors to not claim causality (that per definition cannot be shown by ML alone), the phrasings are still very suggestive, e.g. from the abstract: "In conclusion, peer review in journals with higher journal impact factors tends to be more thorough in discussing the methods used...". There is, however, also the mention of a confounding factor. The authors say that reviewers for high impact journals tended to come from a certain geographic (Europe/NA). So, maybe researchers in Europe/NA are trained to be more focused on methods? Given this confounder and the fact that ML is based on correlation, any conclusions drawn from this study should be phrased much more carefully than currently.

Response: Thank you, point well taken. We never wanted to imply that the association is causal. The journal impact factor cannot directly influence the characteristics of peer review. We argue that the association is not easily explained by any other variable, and, therefore, may be real. We have revised the wording throughout the paper, avoiding causal language.

8) General comment

Given how the study was based on available peer reviews the categories for very high impact of course contain those high-impact journals but very well known highest-impact journals like new england journal of medicine are not in the top ten. Are such flagship journals even present in the sample? If not that´s a shortcoming and a limitation. The authors should comment on this.

Response: S1 File lists all 1664 journals and the number of papers included in the sample. Flagship journals are included in the sample: The New England Journal of Medicine, the Lancet, the BMJ, twelve Nature journals, six PloS journals (other than PloS ONE) are all included, although not with many peer reviews. We now clarify in the paper that Table 1 includes the ten journals with the most reviews per JIF group, rather than the journals with the highest impact factors per group. Since the reviews were drawn randomly (and reviews for some journals appear more often in the Publons database), we do not have any control over the number of reviews. We will consider analyzing these journals in greater detail in a future study.

Overall, the methodological shortcomings of the study make it hard for me find the results trustworthy. The ML modelling should be performed completely and according to state-of-the-art and conclusions should only be drawn on data generated by those final models.

Response: We believe the extensive revisions have made the results more convincing and robust. The correlations between human codings and the machine learning approach is much higher than before, and the classification performance is very good, given the ambiguity of some of the sentences. While the performance is much better, the substantive conclusions remain the same, indicating that our findings do not depend on a specific classifier.
