# Supplementary material for: Relationship between journal impact factor and the thoroughness and helpfulness of peer reviews
Source: PLoS Biol. 2023 Aug 29;21(8):e3002238. doi: 10.1371/journal.pbio.3002238 (PMC10464996; doi:10.1371/journal.pbio.3002238)
Supplement: Attachment — Submitted filename: Authors response R3.docx [file pbio.3002238.s026.docx]

Authors’ response to editorial and reviewer comments (R3):

Severin et al. Relationship between Journal Impact Factor and the Thoroughness and Helpfulness of Peer Reviews

Thank you for your patience while we considered your revised manuscript "Journal Impact Factor and Peer Review Thoroughness and Helpfulness: A Supervised Machine Learning Study" for consideration as a Meta-Research Article at PLOS Biology. Your revised study has now been evaluated by the PLOS Biology editors, the Academic Editor, and the original reviewers. In light of the reviews, which you will find at the end of this email, we are pleased to offer you the opportunity to address the [comments/remaining points] from the reviewers in a revision that we anticipate should not take you very long. We will then assess your revised manuscript and your response to the reviewers' comments with our Academic Editor aiming to avoid further rounds of peer-review, although might need to consult with the reviewers, depending on the nature of the revisions.

# We are grateful to the editors and reviewers for their constructive comments which have greatly helped improve our paper.

# a) Reviewer #1 raises a potentially important point about your decision to normalise by length, and indeed all three reviewers mention the way that review length was treated in this study. Please address these and the other concerns raised by the reviewers.

# We have addressed this comment by adding an additional analysis of the number of sentences in a review addressing the different dimensions of thoroughness and helpfulness – in addition to the percentage of sentences addressing these dimensions (Figures 1, 2, 4 and SI File 3). See below for more details.

# b) Please could you change the Title to "Relationship between Journal Impact Factor and the thoroughness and helpfulness of peer reviews"? Normally we would ask you to incorporate the specific finding(s) in the title, but these are somewhat complex and nuanced, and may change in response to the reviewers' comments.

# Done, thank you.

# c) Please ensure that you comply with our Data Policy; specifically, we need you to supply the numerical values underlying Figs 1, 2, 3, S1.1, S2.1, S2.2, S2.3, S2.4, and the 4 Figs in “S3 File”, either as a supplementary data file or as a permanent DOI’d deposition. We note that you have plans to deposit the data and R code in the Harvard Metaverse; however, I will need to see this before accepting the paper for publication, and we will also need you to make a permanent DOI’d version (e.g. in Zenodo).

# Done, thank you.

# Please cite the location of the data clearly in all relevant main and supplementary Figure legends, e.g. “The data underlying this Figure can be found in S1 Data” or “The data underlying this Figure can be found in https://doi.org/10.5281/zenodo.XXXXX”

# Done, thank you.

# **Reviewer #1:**

# [identifies himself as Ludo Waltman]

# I am pleased to see the significant improvements made by the authors to their paper. I have one remaining comment.

# Thank you

# 1. The authors conclude that "this study of peer review characteristics indicates that peer review in journals with higher impact factors tends to be more thorough in addressing study methods but less helpful in suggesting solutions or providing examples". As pointed out in my previous review, I don't think this conclusion is warranted. It disregards the fact that review reports in journals with higher impact factors are much longer, on average, than review reports in journals with lower impact factors. The percentage of sentences in review reports that suggest solutions or provide examples is lower for higher impact factor journals than for lower impact factor journals, but the absolute number of sentences suggesting solutions or providing examples is higher, not lower. In my view, the conclusion therefore should be that journals with higher impact factors provide reviews that are more, not less, helpful in suggesting solutions or providing examples.

Thank you. We were interested in the emphasis of review reports and therefore analysed the percentage of sentences devoted the different domains of thoroughness and helpfulness. We have now added an analysis of the number of sentences addressing these domains. The last sentence in the Introduction now reads:

*Specifically, we examined the impact factor’s relationship with the absolute number and the proportion of sentences related to peer review thoroughness and helpfulness.*

In the Results we describe the findings as follows:

*The average number of sentences addressing each of the eight content categories in the set of 10,000 reviews ranged from 1.6 sentences on Importance and Relevance to 9.2 sentences on Materials and Methods (upper panel of Fig 1).*

*Fig 2 shows the estimated number of sentences addressing the eight content categories across the ten Journal Impact Factor groups. For all categories, the number of sentences increased from Journal Impact Factor groups 1 to 10. However, increases were modest on average, amounting to two or fewer additional sentences. The exception was Materials and Methods, where the difference between Journal Impact Factor groups 1 and 10 was 6.5 sentences on average.*

*The association between journal impact factor and the eight content categories was analysed in two regression analyses. The first predicted the number of sentences of each content category across the ten Journal Impact Factor groups; the second the changes in the percentage of sentences addressing content categories. All coefficients and standard errors are available from S3 File.*

*The predicted number of sentences are shown in Fig 4 with their 95% confidence intervals (CI). The results confirm those observed in the descriptive analyses. There was a substantial increase in the number of sentences addressing Materials & Methods from Journal Impact Factor group 1 (6.1 sentences; 95% CI 5.3 to 6.8) to group 10 (12.5 sentences; 95% CI ), for a difference of 6.4 sentences (95% CI for difference xx to xx). For the other categories only small increases were predicted, in line with the descriptive analyses.*

In the Discussion:

*We found that the impact factor was associated with the characteristics and content of peer review reports and reviewers. The length of reports increased with increasing Journal Impact Factor. The number of relevant sentences increased in all content categories, but in particular for materials and methods. Expressed as the percentage of sentences addressing a category (and thus standardizing for the different lengths of peer review reports), the prevalence of sentences providing suggestions and solutions, examples or addressing the reporting of the work declined with increasing Journal Impact Factor.*

In the description of the statistical analysis in the Methods section:

*The association between peer review characteristics and* Journal Impact Factor *groups was examined in two ways. The analysis of the number of sentences for each category used negative binomial regression models. The analysis of the proportion of sentences addressing content categories relied on linear mixed-effects models. To account for the clustered nature of the data, we include random intercepts for journals and reviewers [48]. The regression models take the form,*

$$Y_{i}=\alpha_{j\left[ i \right],k\left[ i \right]}+\sum_{m=2}^{10} \beta_{m}\mathbb{\cdot I}\left( JIF_{i}=m \right)+\epsilon_{i}$$

*with*

$\alpha_{j}\sim N\left( \mu_{\alpha_{j}},\sigma_{\alpha_{j}}^{2} \right)$*, for journal* $j=1,\ldots,J$

$\alpha_{k}\sim N\left( \mu_{\alpha_{k}},\sigma_{\alpha_{k}}^{2} \right)$*, for reviewer* $k=1,\ldots,K$

$$\epsilon_{i}\sim N\left( 0,\sigma^{2} \right)$$

*where* $Y_{i}$ *is the count of sentences addressing a content category (for the negative binomial regression models) or the percentages (for the linear-mixed effects models) while* $i$*,* $\beta_{m}$ *are the coefficients for the* $m=2,\ldots,10$ *categories of the categorical variable of Journal Impact Factor (with* $m=1$ *as the reference category), and* $\epsilon_{i}$ *is the unobserved error term. The model includes varying intercepts* $\alpha_{j\left[ i \right],k\left[ i \right]}$ *for* $J$ *journals and* $K$ *reviewers.* $\mathbb{I}\left( \cdot\right)$ *denotes the indicator function.*

*All regression analyses were done in R (version 4.2.1). The fine-tuning of the classifier and sentence-level predictions were done in Python (version 3.8.13). The libraries used for data preparation, text analysis, supervised classification, and regression models, were transformers (version 4.20.1) [49], quanteda (version 3.2.3) and quanteda.textstats (version 0.95) [50], lme4 (version 1.1.30) [51], glmmTMB (version 1.1.7) [52], ggeffects (version 1.1.5) [53], and tidyverse (version 1.3.2) [54].*

# In their response to my previous report, the authors point out that "our analyses controlled for the length of peer review". This is exactly the problem. All statistics presented in the paper are percentages rather than absolute numbers, so the authors indeed control for the length of a review report. However, length is a relevant factor that, I would argue, one should not necessarily control for. For instance, suppose we have two review reports. One has a length of 100 words, 50 of which are used to provide suggestions. The other has a length of 1000 words, 200 of which are used to provide suggestions. From a relative point of view, the former report is more helpful in providing suggestions (50% vs. 20% of the words are used to provide suggestions), but from an absolute point of view, the latter report is more helpful in providing suggestions (50 vs. 200 words are used to provide suggestions). In my view, the absolute perspective is more relevant. The latter report is the one that will be more helpful for authors to improve their work.

Thank you. Yes, by analyzing the percentage of sentences addressing a category we standardised for the different lengths of peer review reports. But we additionally adjusted for any differences in characteristics between shorter and longer peer review reports in our statistical analysis. The latter adjustment made little difference and in the revised version do no longer adjust for length. Rather, we present both the analysis of the number of sentences and the percentage addressing the eight dimensions of thoroughness and helpfulness. The absolute perspective shows that indeed the number of sentences increased for all categories, but increases were driven by additional text on Materials and Methods. This meant that from a relative perspective, the focus of the average review moved as the impact factor increased: away from comments on Presentation and Reporting and Suggestions and Solutions to the Material and Methods.

# More generally, the fact that review reports in the highest impact factor category are more than twice as long as review reports in the lowest impact factor category is of major importance and, in my view, needs to be emphasized more strongly. It indicates that higher impact factor journals tend to offer more in-depth peer review than lower impact factor journals. This is an important finding that I believe should be mentioned In the abstract and in the concluding section.

Thank you. This finding is now mentioned both in the abstract and the concluding paragraph.

In the Abstract:

*The median length of reviews increased with higher impact factor, from 185 words (group 1) to 387 words (group 10). … In conclusion, peer review in journals with higher impact factors tends to be more thorough, particularly in addressing study methods while giving relatively less emphasis to presentation or suggesting solutions. Differences were modest and variability high, indicating that the Journal Impact Factor is a bad predictor of the quality of peer review of an individual manuscript.*

Concluding paragraph:

*This study of peer review characteristics indicates that peer review in journals with higher impact factors tends to be more thorough, particularly in addressing the study’s methods while giving relatively less emphasis to presentation or suggesting solutions. Our findings may have been influenced by differences in reviewer characteristics, quality of submissions, and the attitude of reviewers towards the journals. Differences were modest, and the Journal Impact Factor is a bad predictor of the quality of peer review of an individual manuscript.*

# PS I published my previous review online. I had hoped to also publish my new review. However, it seems the authors haven't posted their revised paper on a preprint server. I therefore consider the revised paper to be confidential and I won't publish my review.

# Thank you.

# **Reviewer #2:**

# [identifies himself as Bernd Pulverer]

# The authors are to be commended for the thorough responses.

# Thank you.

# A number of comments:

# 1) I appreciate the point that many journals with open review processes share these on Publons (now part of Clarivate 'Web of science'). However, Journal with public but unsigned reports less so. Nonetheless, I agree that scraping the literature for non-Publons listed reports in the absence of standardize identifiers is not trivial. I do believe ORCID profiles can point to referee reports from the 'Review URL' field (cf. https://support.orcid.org/hc/en-us/articles/360006971333-Peer-Review). This study is based on a large dataset and it is certainly reasonable to restrict the study to this dataset as it is unclear if a broader set of input data would alter the conclusions significantly. These points could be discussed.

# Thank you. The reviewer made this point previously. We are glad that he now acknowledges that it is reasonable to restrict the study to this dataset. We discuss the limitations and potential biases in the Discussion section.

# 2) Thank you for pointing out that signed vs. unsigned reports and referee reports on research article vs. reviews was not assessed - that is fine, but I am unclear why signed reports could not be automatically identified and compared to unsigned reports. Note that I had suggested a third comparison between published and unpublished referee reports, but acknowledge that while very interesting, this would be a complex undertaking that can be discussed.

# Thank you. We apologize for not make this clear but there is no variable in the database that identifies signed reports. We have clarified this as follows:

*Due to the lack of information in the database, we could not analyze any differences between open (signed) and anonymous peer review reports.*

# 3) - 6) Thank you for the constructive comments and revision

# The minor points are addressed, apart from point 3): I would recommend to highlight more clearly that automated analysis of referee reports for quality attributes may inform a referee credit system that could be used objectively and at scale in research assessment by funders and research institutions.

Thank you. We agree and have added the following sentence in the Discussion section:

*Further, automated analysis of peer review reports could inform a reviewer credit system that could be used by funders and research institutions. for*

# I assessed the responses to ref #1 and #3 and, leaving aside the technical details on statistics and models, which I did not judge, I believe the responses are thorough and the revisions comprehensive leading to a more informative and balanced manuscript. In particular the causality point by ref 3 (no 7) is important and was addressed.

# It may be worth emphasizing the referee report length more, both as a correlation with JIF and in the context of the length control applied here (as discussed in ref #1, point 10; ref #2, minor point2).

Thank you. We now pay much more attention to the length of peer review reports – see responses to referee #1 above.

# **Reviewer #3:**

# The re-work of the manuscript was extensive, the authors have addressed all relevant shortcomings very well. I have only two minor comments, that should be addressed imo before publication.

Thank you.

# 1) discussion, p. 12

# "Our study shows that the peer reviews submitted to journals with higher Journal Impact Factor may be more thorough than those submitted to lower impact journals. Should, therefore, the Journal Impact Factor be rehabilitated, and used as a proxy measure for peer review quality? "

# In the following discussion of this question, and also at other parts of the manuscript, there is the implicit assumption that submitted peer reviews are independent of the impact factor and journal, i.e. the same effort is put into providing a review. But of course that is likely not true. That reviews tend to be shorter and less thorough w/r to methodology in lower impact journals can have two additional confounding factors:

# a) People are less thorough _because_ it is a low impact journal, believing they do not need to provide a review of as good quality as for a journal with a higher impact factor.

# b) People might also be less thorough, when only basing this on the length(!), because the quality does not warrant more text. Let me explain. If I am confronted with an applied AI in healthcare methodology, that is completely not up to the standards, I might just write exactly that and give some examples in bullet points, and suggest rejection. Confronted with a good methodology that has only _some_ major shortcomings, I will likely take the time (and words) to explain these few shortcomings. My point here is that longer -> "more thorough" does not necessary mean more useful or better. The former case does not _warrant_ more text (and it also does not warrant many suggestions. Is it true that I find the former more often in low impact journals? I do not know. I had one of my worst experiences in this regard in the flagship journal of my field (and the paper was accepted despite the fact that I suggested a reject as the only AI methodology expert). But I think this point could still be considered.

# I believe that the discussion would improve if these additional points were also discussed as potentially confounding factors and why this topic is very hard to assess.

Thank you. We have extended the corresponding paragraph in the Discussion section as follows:

*The increase in the length of peer review reports with increasing Journal Impact Factor might be explained by the fact that reviewers from Europe and North America and reviewers with English as their first language tend to write longer reports, and to review for higher impact journals [20]. Further, high impact factor journals may be more prestigious to review for and can thus afford to recruit more senior scholars. Of note, there is evidence suggesting that the quality of reports decreases with age or years of reviewing [21,22]. Interestingly, several medical journals with high impact factors have recently committed to improving diversity among their reviewers [23–25].‬‬‬‬‬‬‬‬‬‬‬‬ Unfortunately, due to incomplete data, we could not examine the importance of the level of seniority of reviewers.‬‬‬‬ Independently of seniority, reviewers may be brief reviewing for a journal with low impact factor, believing a more superficial review will suffice. On the other hand, brief reviews are not necessarily superficial: the review of a very poor paper may not warrant a long text.*

# 2) conclusion, p. 14

# "This study of peer review characteristics indicates that peer review in journals with higher impact factors tends to be more thorough in addressing study methods but less helpful in suggesting solutions or providing examples."

# I believe that this sentence should be followed by something like (authors should modify as they wish): "These differences may also be influenced by differences in geographical reviewer characteristics, quality of submissions, and the attitude of reviewers towards the journals".

# Otherwise the conclusion implies, at least to a degree, that higher impact may lead to more thorough reviews.

Thank you. We have rephrased the Conclusion section as follows:

*This study of peer review characteristics indicates that peer review in journals with higher impact factors tends to be more thorough, particularly in addressing the study’s methods while giving relatively less emphasis to presentation or suggesting solutions. Our findings may have been influenced by differences in reviewer characteristics, quality of submissions, and the attitude of reviewers towards the journals. Differences were modest, and the Journal Impact Factor is a bad predictor of the quality of peer review of an individual manuscript.*
